# Identifying contextual barriers and facilitators in implementing non-specialist interventions for mental health in Sri Lanka: A qualitative study with mental health workers and community members

**DOI:** 10.1017/gmh.2024.75

**Published:** 2024-10-08

**Authors:** Kalpani Wijekoon Wijekoon Mudiyanselage, Frederike Jörg, Murukkuvadura Sajani Dilhara Mendis, Daniela C. Fuhr, Heide Busse

**Affiliations:** 1 Faculty 11 Human and Health Sciences, University of Bremen, Bremen, Germany; 2 Department of Prevention and Evaluation, Leibniz Institute for Prevention Research and Epidemiology- BIPS, Bremen, Germany; 3 University of Groningen, University Medical Center Groningen, University Center for Psychiatry, Interdisciplinary Centre for Psychopathology and Emotion Regulation, Groningen, The Netherlands; 4 Faculty of Social Sciences, International College of Business and Technology, Colombo, Sri Lanka; 5 Cardiff School of Sport and Health Sciences, Cardiff Metropolitan University, Cardiff, UK; 6 Leibniz ScienceCampus Digital Public Health Bremen, Bremen, Germany

**Keywords:** barriers, developing countries, global mental health delivery, healthcare workers, mental health

## Abstract

Non-specialist mental health interventions serve as a potential solution to reduce the mental healthcare gap in low- and middle-income countries, such as Sri Lanka. However, contextual factors often influence their effective implementation, reflecting a research-to-practice gap. This study, using a qualitative, participatory approach with local mental health workers (n = 9) and potential service users (n = 11), identifies anticipated barriers and facilitators to implementing these interventions while also exploring alternative strategies for reducing the mental healthcare gap in this context. Perceived barriers include concerns about effectiveness, acceptance and feasibility in the implementation of non-specialist mental health interventions (theme 1). The participants’ overall perception that these interventions are a beneficial strategy for reducing the mental healthcare gap was identified as a facilitating factor for implementation (theme 2). Further facilitators relate to important non-specialist characteristics (theme 3), including desirable traits and occupational backgrounds that may aid in increasing the acceptance of this cadre. Other suggestions relate to facilitating the reach, intervention acceptance and feasibility (theme 4). This study offers valuable insights to enhance the implementation process of non-specialist mental health interventions in low-and middle-income countries such as Sri Lanka.

## Graphical abstract

**Figure:**
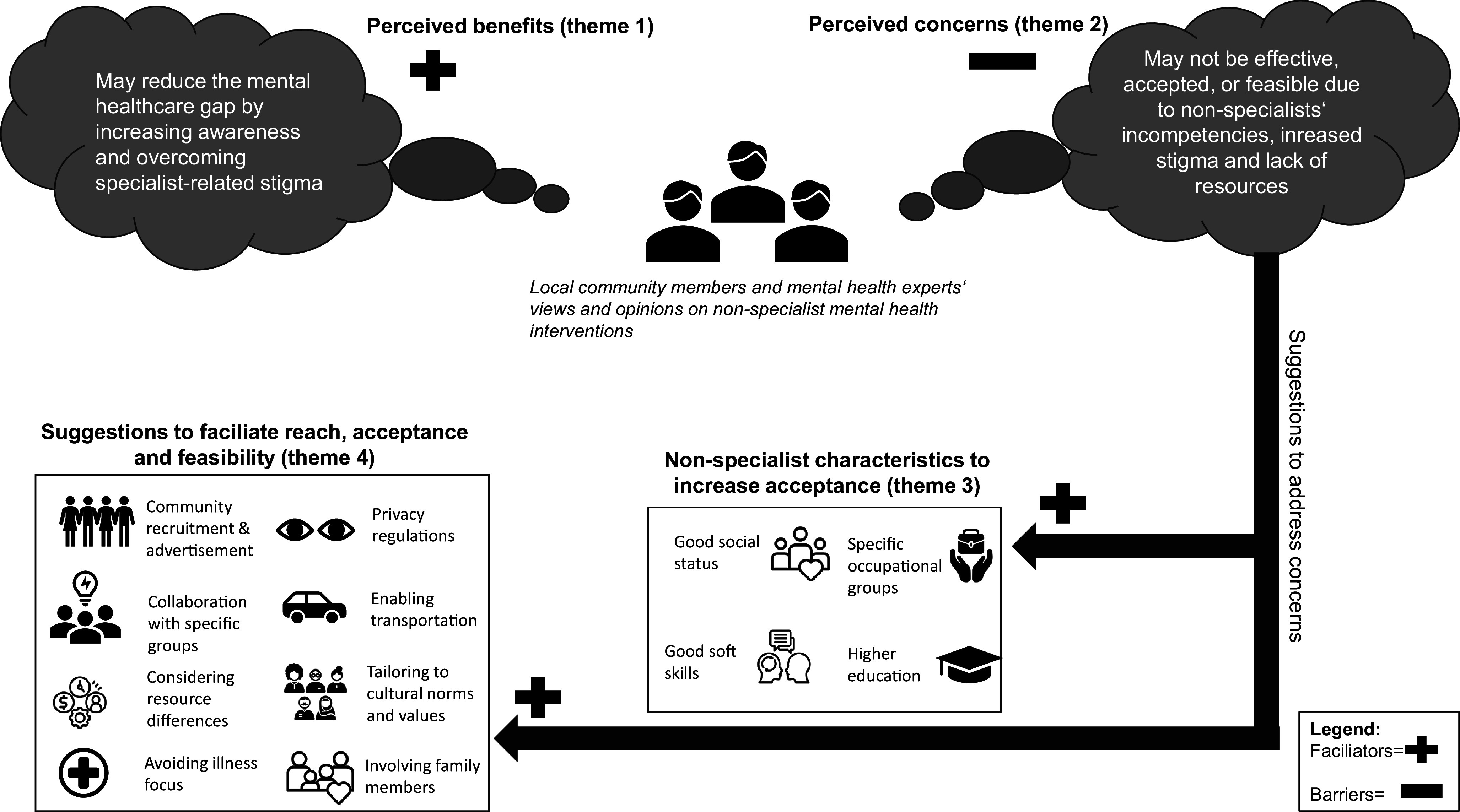

## Impact statement

A primary aim of the global mental health movement is to enhance overall mental well-being and achieve health equity worldwide. The significant challenge posed by the global mental healthcare gap has led to the recognition of non-specialist mental healthcare providers as a vital resource for expanding care capacity. However, the effectiveness of deploying evidence-based interventions in new settings depends on a thorough understanding of those specific local contexts to ensure interventions are successfully implemented. Understanding the local context is crucial as various local factors may either impede or facilitate the smooth integration and effectiveness of health interventions. Therefore, this research sheds light on factors anticipated to impact the implementation of non-specialist interventions, thereby attempting to bridge the research-practice gap in Sri Lanka and similar settings. By delving into the unique needs of potential service users and gathering insights from local experts, the study offers essential guidance for the effective implementation of evidence-based non-specialist mental health interventions. Our findings hold particular significance for researchers and policymakers interested in introducing non-specialist interventions in Sri Lanka, underlining the necessity of customising these strategies to fit the local context’s cultural, normative and resource-specific nuances to close the mental healthcare gap effectively.

## Introduction

Mental disorders accounted for 13% of global disability-adjusted life-years in 2019, paralleling the burden of cardiovascular diseases (Vigo et al., 2022). However, a significant gap exists between the need for and receipt of evidence-based mental healthcare (MHC), particularly in low- and middle-income countries (LMICs), which can be attributed to the lack of supply (Demyttenaere et al., 2004; Saxena et al., 2007; Barnett et al., 2019) and low demand for MHC (Barnett et al., 2019).

One example of an LMIC is Sri Lanka. Sri Lanka, an island nation in South Asia, has experienced significant impacts on its population’s mental health due to a 30-year-long civil war, the 2004 tsunami and economic and political crises (Minas et al., 2017; The Lancet Regional Health-Southeast Asia, 2022). A recent meta-analysis has shown that approximately one-fifth of the population suffers from depression, with the highest rates found among young people aged 10–24 years (Alwis et al., 2023). Sri Lanka’s healthcare system is characterised by a government-financed, decentralised public health system complemented by a robust private sector (Jenkins and Cooray, 2012) (details on Sri Lanka’s healthcare system can be found in Supplementary Material S1). Aligning with the key strategies addressed in the 1978 Alma Ata Declaration by the World Health Organisation (WHO), Sri Lanka has pursued a community-based mental health system that integrates MHC into primary healthcare, creating unique MHC roles to facilitate this endeavour (World Health Organization, 1978; Fernando et al., 2017). Examples are the Medical Officers with a Diploma in Psychiatry and Medical Officers of Mental Health (MOMH), who are medical graduates with either a one-year diploma or a 1-month certificate in psychiatry, respectively (Minas et al., 2017). Further details on measures to address Sri Lanka’s MHC gap are in Supplementary Material S1.

Sri Lanka has significantly improved its MHC situation, yet faces considerable challenges (Fernando et al., 2017). In 2020, it had only 5.46 mental health workers per 100,000 people, well below the ≥60 median of high-income countries (World Health Organization, 2021, 2022). The dominance of medical over psychological interventions underscores the need for broader mental health services (Fernando et al., 2017). High patient loads due to reliance on publicly funded healthcare strain medical staff, including primary care physicians and MOMHs (Jenkins and Cooray, 2012; Fernando and Samaranayake, 2019). Recent economic and COVID-19 crises have exacerbated this by increasing medical staff emigration (The Lancet Regional Health-Southeast Asia, 2022). Furthermore, stigma and low mental health literacy continue to expand the MHC gap (Fernando, 2010; Samarasekare et al., 2012; Knaak et al., 2017).

These insights indicate that further development of the MHC system, aligning with global mental health agendas such as the sustainable development goals (SDG), is necessary (Fernando et al., 2017; Patel et al., 2018). One of the goals outlined in the SDGs includes building human resource capacity by redefining who can provide mental health interventions (Patel et al., 2018). In the past decade, there has been a growing body of endeavours in which individuals who do not have a formal specialisation in mental health receive a brief training, enabling them to provide mental health promotion, prevention and low-intensity treatment interventions, mostly for people with non-severe mental disorders (van Ginneken et al., 2013; Barnett et al., 2019; Purgato et al., 2023). These individuals can be either workers from the medical field (i.e., general medical doctors) or lay workers outside the medical field (van Ginneken et al., 2013; Barnett et al., 2019; Keynejad et al., 2021). A wide range of terms have been used to describe this type of providers (van Ginneken et al., 2013; Barnett et al., 2019; van Ginneken et al., 2021). This paper utilises the term “non-specialist MHC workers”, a term frequently used in previous studies (van Ginneken et al., 2013; Chowdhary et al., 2014; Patel et al., 2018; Tareke et al., 2023), to underscore the inclusion of individuals from both the medical and non-medical fields who can provide promotional, preventive and treatment interventions.

Common non-specialist mental health approaches in LMICs include the outreach and task-sharing approaches (Barnett et al., 2019). Non-specialists in outreach approaches are responsible for bridging the gap between the community and the healthcare system by, for example, providing mental health education programmes and referrals to specialists (Barnett et al., 2019; Phoeun et al., 2019). Task-shifting refers to moving specific prevention or treatment tasks from highly qualified health workers to non-specialists with fewer qualifications through brief evidence-based training and constant specialist supervision (Barnett et al., 2019). These treatment interventions can be further distinguished into two types, depending on the involvement of the specialist in care provision: the stepped-care approach and the sole-provider approach. In stepped-care interventions, non-specialists initially offer preventive-level interventions while the specialist provides more intensive care. These interventions are typically characterised by a collaborative care approach in which a specialist MHC worker can be involved in the care process (Patel et al., 2010). In certain resource-poor settings, where no specialists are available, non-specialists are the sole providers of brief psychological interventions (Barnett et al., 2019, 2023). Hence, a key differentiator between these two interventions may lie in the collaborative care approach, with multiple possible care providers, as observed in stepped-care interventions (Patel et al., 2010) in contrast to the sole-provider care interventions (Sabir Ali et al., 2003). More recently, digital technologies have also been integrated to train, supervise and support non-specialists in intervention delivery, which could further address the shortcomings of available human workforces in LMIC contexts (Wijekoon Mudiyanselage et al., 2024).

Several randomised controlled trials and feasibility studies have demonstrated a reduction in mental health symptoms, user acceptability and delivery feasibility of brief psychosocial transdiagnostic MHC interventions mainly delivered by non-specialist community workers (Tol et al., 2020; Fine et al., 2021; Hamdani et al., 2021; Acarturk et al., 2022; Bryant et al., 2022; Brown et al., 2023; de Graaff et al., 2023; Schäfer et al., 2023). Examples include the Problem Management Plus (Hamdani et al., 2021; Acarturk et al., 2022; de Graaff et al., 2023), Early Adolescent Skills for Emotion (Fine et al., 2021; Bryant et al., 2022; Brown et al., 2023) and Self-help Plus interventions (Tol et al., 2020). Additionally, several structured training programmes for primary healthcare workers have shown benefits in improving patients’ well-being as well as knowledge and skills of these care providers (Sadik et al., 2011; Jenkins et al., 2013; Kauye et al., 2014).

With regard to Sri Lanka, a few non-specialist mental health interventions were tested and showed benefits. For instance, the “Train the Trainers” programme, developed with WHO support, involves MOMHs leading a concise 5-day training course for health workers. This initiative aims to equip non-specialist medical professionals with skills to assess, diagnose and treat common mental and neurological disorders (Jenkins and Cooray, 2012). In terms of non-specialist-delivered promotional and preventive interventions, only a limited number of non-specialist outreach interventions have been tested. These interventions have positively impacted the well-being and mental health of service users, while also highlighting the need to expand prevention and treatment interventions (Jordans et al., 2010; Tol et al., 2012; Chandrasiri et al., 2015).

Given Sri Lanka’s existing initiatives in community-based care and the concurrent pressing need for expansion and enhancement, further implementation of non-specialist outreach and task-sharing interventions represents a viable strategy. This approach could involve brief training programmes within the medical field, such as train-the-trainer programmes (Jenkins and Cooray, 2012), but should also extend to training individuals outside the medical field. These trainees could then deliver psychosocial treatment either as part of stepped-care approaches or as sole providers to make more efficient use of the existing resources. Additionally, the expansion of evidence-based promotional and preventive outreach interventions is warranted to bolster the existing MHC framework.

Implementing and translating scientifically validated interventions in real-world environments is complex and challenging. This difficulty arises from the research-to-practice gap, which is marked by notable differences between the efficacy of interventions in controlled settings and their effectiveness when applied in community settings (Nilsen and Bernhardsson, 2019). Previous studies indicate that the implementation of non-specialist interventions still faces substantial, context-specific obstacles (Faregh et al., 2019). Particularly, the type of task-sharing intervention that can be successfully implemented, or the integration of digital technologies in non-specialist interventions highly depends on the existing resources and population preferences (Singh et al., 2022; Barnett et al., 2023). These observations suggest the need for a systematic and scientific method to better understand the context in which these interventions are applied. Crucial to this process is the understanding of contextual implementation determinants, also called barriers and facilitators, which can significantly influence the implementation process of interventions (Le et al., 2022). Research, predominantly relying on user experiences, has extensively explored these implementation determinants, as depicted in a systematic review that specifically focussed on mental health task-sharing interventions in LMICs (Le et al., 2022). Among others, this review underscored the impact of societal and mental health system factors on successful implementation processes. Additionally, it showed how such factors can be broadly relevant to LMICs, yet they may also vary depending on the specific context of each country (Le et al., 2022). While this comprehensive review shows a broad overview of contextual implementation determinants for task-sharing interventions in different LMICs, notably, no trials have been detected in Sri Lanka (Le et al., 2022). This lack of specific data underscores the need for a thorough assessment of these factors within the Sri Lankan context. In implementation science, a proactive assessment of anticipated implementation determinants is vital for contexts lacking comprehensive evidence, as it allows for the direct integration of findings into the implementation strategy (Grol and Wensing, 2004; Proctor et al., 2013; Fernandez et al., 2019).

The general objective of this study is to better understand the Sri Lankan context in order to inform future implementation efforts for non-specialist mental health interventions in Sri Lanka and similar settings. Therefore, the primary aim of this study is to identify anticipated contextual barriers and facilitators to the implementation of common evidence-based non-specialist interventions in Sri Lanka. The secondary aim of this study is to provide insights into other mental health interventions or strategies that are perceived as crucial to bridge the MHC gap in Sri Lanka. To address these aims, this qualitative study employs a participatory approach, involving potential service users and local mental health experts. Its exploration intends to generate vital information for implementation strategies to enhance MHC in LMICs such as Sri Lanka.

## Methods

This study involved interviews with mental health workers, including psychologists, psychiatrists, MOMH’s, counsellors and researchers with tertiary education in mental health, as well as other community members outside the mental health field, such as religious leaders. All participants were over 18 and resided in either Badulla, a rural and economically poor district, or Colombo, an urban and economically affluent district of Sri Lanka (Gnanapragasam, 2021).

Participants were recruited using convenience sampling. Flyers distributed in universities, hospitals and temples by a research assistant (M.S.D.M.) complemented email invitations circulated in schools and peer networks. Direct emails were sent to specific mental health professionals by the main researcher (K.W.W.M.) and the research assistant. Additionally, snowball sampling was employed for further contact recommendations.

Interested individuals contacted the research team via email or phone, received study information and a consent form to sign and return prior to their interview. Interviews were scheduled based on their availability. Of the 42 potential participants approached, 22 opted out. Further details on the recruitment process and reasons for non-participation are available in Supplementary Material S2.

The primary researcher conducted semi-structured interviews online using Zoom from October 2022 to January 2023, with an interpreter (M.S.D.M.) present in five cases. Each interview, lasting about an hour on average (range: 29.06–63.46 min), was audiotaped with an encrypted recorder. At each interview’s start, the researcher outlined the study objectives, addressing participant queries. Tailored interview guides for each participant group (Supplementary Material S3), pilot-tested with a local physician and a community member, were used. Community members’ interviews began with case examples on depressive and psychosis symptoms, inviting solutions to explore their mental health beliefs and attitudes. For all participants, a general explanation of non-specialist mental health interventions was provided followed by a discussion on their knowledge and perceptions on existing interventions in Sri Lanka. Drawing on insights from previous research, the discussion proceeded on to various common intervention types that were tested in other LMICs: a school-based outreach programme (Phoeun et al., 2019), a collaborative stepped-care approach involving lay workers and medical doctors as non-specialists (Patel et al., 2010), and a sole-provider delivery model (Sabir Ali et al., 2003). Participants were prompted to share their general perceptions of these interventions and, in a unique approach, were asked to envision themselves as advisors to a research team aiming to implement these interventions in Sri Lanka. This allowed for the identification of anticipated barriers and facilitators to implementing any mental health interventions involving not only non-specialists but also specific intervention types. Previous research suggested that asking participants to imagine being in someone else’s position can generate similar thoughts (Davis et al., 2004). Finally, the participants were asked to unveil their thoughts about integrating any form of digital technologies in such interventions. We did not assess the mental health status of the participants. However, a previous meta-analysis suggests that the acceptability and preference of mental health interventions in a non-treatment-seeking population are similar to those of a treatment-seeking sample (McHugh et al., 2013).

Following all interviews, the main researcher prepared field notes and discussed the interviews with the research assistant. After conducting 18 interviews, no new initial codes related to the anticipated barriers and facilitators re-emerged. Consequently, two additional interviews were carried out to validate this observation. Following these interviews, the research team collectively determined that a high degree of inductive thematic and data saturation had been achieved (Saunders et al., 2018).

All participants received between 4,500 and 5,500 Sri Lankan rupees (equivalent to 15 Euros) per interview, depending on the varying exchange rates. More details about the characteristics of the research team members can be found in Supplementary Material S4.

**Figure 1.**
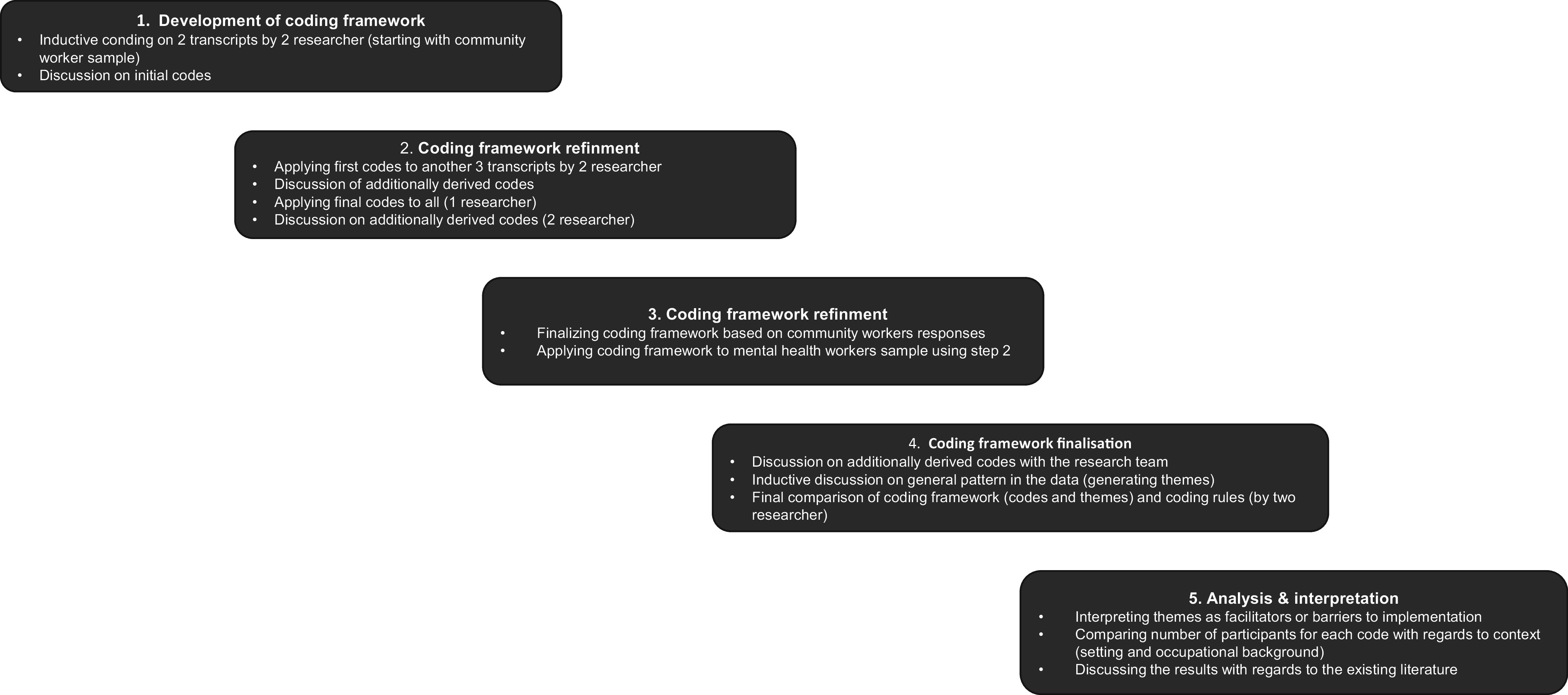
Coding strategy.

Professional transcription of audio recordings was done under a confidentiality agreement, followed by an anonymisation process of the transcripts by the primary researcher. The analysis was guided by Braun and Clarke’s thematic analysis, employing an inductive approach due to the study’s exploratory nature (Braun and Clarke, 2021). After familiarising with the data, a coding framework was developed, using both latent and semantic methods. Initially, our analysis began with coding the transcripts of community members, during which we developed the preliminary versions of the coding framework. We then applied this existing framework to the transcripts of mental health workers to determine whether the identified codes were applicable to this new group of participants or if additional codes emerged (see Figure 1). This process revealed that there were no major differences in the codes across participant groups. To discern more subtle variations between the population groups, in the final stage of our analysis, we categorised and quantified the origins of the codes. This involved distinguishing between individuals from Colombo and Badulla, as well as between mental health workers and other community members, as documented in Table 1. The first 30% of transcripts were coded by two researchers, with team discussions enhancing reflexivity and code interpretation. The primary author then grouped codes into themes, reviewed periodically with the team (Figure 1). MAXQDA software facilitated the analysis (VERBI Software, 2021), and the 32-item Consolidated Criteria for Reporting Qualitative studies checklist was applied for reporting (Supplementary Material S5) (Tong et al., 2007).

**Table 1.** Overview of themes and codes

Code (reference^1^)	Colombo (*N*)	Badulla (*N*)	MHC worker (*N*)	Other community members (*N*)	Total
Overarching theme: Anticipated barriers to implementation					
Theme 1: Concerns about effectiveness, acceptance and feasibility					
*Type: General non–specialist mental health interventions^2^*					
Concern about ineffectiveness (1.1.1)	4	1	1	4	5
Concern about increased stigma and discrimination (1.1.2)	0	1	0	1	1
Concern about privacy (1.1.3)	2	1	1	2	3
Concern about the current economic situation (1.1.4)	1	3	2	2	4
*Type: The collaborative stepped–care intervention type^3^*					
Concern about being too overwhelming (1.2.1)	2	1	0	3	3
Concern about the lack of human resources (1.2.2)	3	2	4	1	5
*Type: The sole–provider intervention type^4^*					
Concern about incompetency to deliver treatment alone (1.3.1)	2	1	1	2	3
*Type: The educational school–based outreach intervention type^5^*					
Concern about increasing discrimination in school settings (1.4.1)	2	2	3	1	4
Concerns about potential disagreement from parents (1.4.2)	5	0	2	3	5
Concerns about the strict education system (1.4.3)	1	1	1	1	2
Concerns about the high workload of teachers (1.4.4)	1	2	2	1	3
*Type: Non–specialist mental health interventions involving digital technologies^6^*					
Concerns about the poor digital infrastructure (1.5.1)	5	4	5	4	9
Theme 2: Perceived benefits in reducing the mental healthcare gap					
*Type: General non–specialist mental health interventions^2^*					
Benefit of increasing access to mental health interventions (2.1.1)	3	3	4	2	6
Beneficial because non–specialists are considered more comfortable to talk to in contrast to specialists (2.1.2)	4	1	2	3	5
*Type: The collaborative stepped–care intervention type^3^*					
Benefit of increasing trust in the effectiveness of treatment (2.2.1)	3	3	3	3	6
*Type: The sole–provider intervention type^4^*					
Beneficial for establishing a close bond with the care provider (2.3.1)	2	2	0	4	4
*Type: The educational school–based outreach intervention type^5^*					
Benefit of increasing the mental health literacy (2.4.1)	7	4	4	7	11
Beneficial for reducing the mental illness burden (2.4.2)	3	3	3	3	6
*Type: Non–specialist mental health interventions involving digital technologies^6^*					
Benefit in making the intervention more attractive (2.5.1)	4	3	3	4	7
Beneficial for facilitating access to services (2.5.2)	5	5	6	4	10
Beneficial for facilitating care coordination (2.5.3)	2	0	2	0	2
Beneficial for facilitating mental health awareness programmes (2.5.4)	3	0	2	1	3
Theme 3: Important non–specialist characteristics to increase acceptance					
*Type: General non–specialist mental health interventions^2^*					
Importance of good social recognition and status (3.1.1)	3	5	1	7	8
Suitable occupational groups (3.1.2)	9	9	7	11	18
Suitable personal characteristics (3.1.3)	4	1	1	4	5
Higher academic status (3.1.4)	2	1	1	2	3
Theme 4: Suggestions to facilitate reach, acceptance and feasibility					
*Type: General non–specialist mental health interventions^2^*					
Advertising interventions (4.1.1)	3	3	3	3	6
Community recruitment (4.1.2)	3	1	1	3	4
Ensuring privacy protection and gaining trust (4.1.3)	2	2	1	3	4
Payment and incentives (4.1.4)	6	3	6	3	9
Collaboration (4.1.5)	2	4	3	2	6
Tailoring to cultural norms (4.1.6)	6	1	4	3	7
Providing means of transportation (4.1.7)	2	0	1	1	2
*Type: The educational school–based outreach intervention type^5^*					
Involving parents (4.4.1)	4	1	4	1	5
Avoiding the illness angle (4.4.2)	2	0	2	0	2
Considering differences in schools (4.4.3)	4	1	3	2	5

Notes: Abbreviations: MHC = mental healthcare.

1 The code definitions and corresponding quotes can be found in the Additional File 9. Definitions of intervention types.

2 The general concept of involving non-specialists in any mental health interventions.

3 A collaborative care type involving three intervention providers. First a non-specialist provides low-level psychosocial preventive intervention (i.e., breathing exercises) and a non-specialist medical doctor is responsible for providing medication and additional support if required while both are supervised by a specialist.

4 One non-specialist provides low-intensity treatment alone, particularly in resource-poor areas where no specialists are available.

5 A school-based outreach intervention type in which teachers receive basic mentalhealth education to teach students on mental health and raise general mental health awareness and to be able to identify, refer and deal with children with mental health problems.

6 Any involvement of any type of digital technology in any non-specialist mental health interventions.

## Results

Out of 20 participants, 9 were mental health experts and 11 were community members, with most community participants being men from Badulla and most mental health expert participants being women from Colombo (Participant characteristics in Supplementary Material S6). The case vignettes were generally understood as states of emotional distress by community members. However, in some cases, participants distinguished between depressive and psychotic symptoms, often considering depressive symptoms as normal emotional states while viewing psychotic symptoms as more concerning. Effective solutions included seeking support from trusted individuals or specialist care, though stigma was a concern (Health beliefs among community members in the Supplementary Material S7). Most participants knew of existing non-specialist interventions in Sri Lanka, like school prevention programmes and care by trained or untrained non-specialists. Mental health workers expressed concerns regarding the regulation of these interventions, particularly noting that non-specialists might not receive adequate evidence-based training or proper supervision. Despite these concerns, they acknowledged the potential benefits of employing non-specialists to improve access to mental health services, provided that appropriate education and supervision are ensured (Knowledge and opinions on existing non-specialist interventions in the Supplementary Material S8).

### Anticipated barriers and facilitators

Four key themes emerged concerning perceptions on anticipated implementation determinants of non-specialist mental health interventions: *Perceived concerns about effectiveness, acceptance and feasibility* (theme 1), *perceived benefits in reducing the MHC gap* (theme 2), *important non-specialist characteristics to increase acceptance* (theme 3) and *suggestions to facilitate reach, acceptance and feasibility* (theme 4). These themes encompass factors that pertain to the general concept of employing non-specialists in mental health interventions and those specific to distinct intervention types (Table 1, Coding framework in the Supplementary Material S9).

#### Theme 1: Concerns about effectiveness, acceptance and feasibility

Concerns about effectiveness were mainly raised by community members and linked to the belief of non-specialists’ limited competencies and potential adverse effects, particularly in sole-provider treatment roles, contrasted with the involvement in collaborative care teams.

*“[the sole-provider intervention type] is not a very successful thing. I think that they don’t have much knowledge. […] If it is a group or if it is a community, that will be all right. But a person, I don’t think that they can handle the situation like this.” (Banker, man, Badulla).*

Yet other participants, also mainly other community members, perceived the collaborative care approach as ineffective because of the danger of being *“too overwhelming”* (Freelance writer, man, Colombo) and requiring a *“complex communication system”* (Midwife, woman, Badulla) given the potential involvement of many providers.

Furthermore, community members and mental health workers raised concerns about the lack of acceptance for non-specialist interventions, primarily due to the prevalent mental health stigma in Sri Lanka. This stigma would impact both non-specialists and specialists. For instance, a school principal from Badulla pointed out that providers themselves might be stigmatised as *“mad”*, potentially posing challenges for recruiting suitable non-specialists. Additionally, issues of stigma originating from providers, especially in educational settings, were highlighted, indicating complexities in implementing these interventions:

*“So even though a teacher identifies, ‘Okay, this child is struggling with concentration, is struggling with emotional regulation, I have to refer them to a psychologist.’ That may not change the way that they treat the child, but they might still be discriminatory towards a child within a classroom setting, even though they have known that there is something wrong because there is an internal bias that is impacting that.” (Mental health worker, woman, Colombo).*

Also, community members and mental health workers assumed that non-specialists handling sensitive data might misuse personal information, unlike specialists. This may also contribute to a general reluctance to seek this type of care.

*“The problem is the privacy. They are not going to keep the privacy of the person. It is like gossip. They are always not going to keep that a secret, it should be a secret because it is an illness.” (Mental health worker, man, Badulla).*

Moreover, participants from both groups expressed feasibility concerns, primarily due to resource constraints possibly intensified by the economic crisis. This could further extend the shortage of specialists and healthcare workers needed for on-site supervision and training of non-specialists, collaborative or school-based interventions and may also impact digital infrastructure, affecting its involvement in such interventions. Particularly mental health workers raised concerns about the feasibility of implementing collaborative stepped-care approaches in light of the lack of medical personnel.

#### Theme 2: Perceived benefits of non-specialist mental health interventions in reducing the MHC gap

Participants from both groups recognised that despite concerns, incorporating non-specialists in MHC is crucial for expanding the workforce, enhancing care accessibility and affordability of MHC, increasing awareness and reducing stigma linked to specialist care. They saw non-specialists as potentially more approachable and “*comfortable*” conversation partners (IT worker, woman, Colombo).

*“We don’t have much people [specialists]. And we do have to promote these non-specialised communities [groups], and it’ll help this kind of people to get rid of these problems.” (Banker, man, Badulla).*

Furthermore, mental health workers and community members saw digital technologies as crucial in making mental health programmes more engaging and accessible, especially in rural areas.

*“I’m sure like even the people in the rural areas, it would be a lot cooler if you had like a digital presentation for them. They’d be pretty amazed by it also” (Freelance writer, man, Colombo).*

Moreover, specifically mental health workers reported that technology was believed to effectively facilitate care coordination and thus overcome current organisational intervention challenges, which is depicted as “*complicated and a lot of writing. And a lot of, I will say, paperwork that needs to be done.” (Mental health worker, woman, Colombo).* Other intervention types were also valued for their unique qualities. For example, the stepped-care intervention type was specifically valued as effective due to the collaborative care team, particularly involving a general practitioner:

*“The second thing is, the non-specialised person with a doctor, that will be a great opportunity because the doctor will understand the health condition. And other person, she can talk to the person and get the details. And that combination will be great.” (Banker, man, Badulla).*

The sole-provider intervention type was regarded as valuable, especially by other community members, as they believed it would help create a close bond with the service deliverer due to a seemingly simplified conversation pathway that does not involve many providers.

*“[The sole-provider intervention type] as a more direct and simpler communication method. […] It will empower the grassroots person to actually provide their clients with more holistic and a more consistent set of answers and solutions to their problem, as opposed to this client going to have to go to like three different levels to get like their treatment in three different parts.” (Midwife, woman, Badulla).*

The school-based educational outreach intervention type was valued by both participant groups for its potential to increase the overall mental health literacy of both children and teachers and was believed to effectively reduce the existing mental illness burden in students, particularly as a consequence of the COVID-19 pandemic and high academic pressure.

#### Theme 3: Important non-specialist characteristics to increase acceptance

Participants offered key insights on selecting the ideal non-specialists for mental health interventions that could increase the acceptance of this cadre. Particularly community members stressed the importance of choosing individuals with strong social standing and recognition. Participants from both groups identified occupations like midwives, teachers, social workers, religious leaders and female police officers as ideal non-specialists due to their community rapport. Additionally, personal traits such as soft skills, coping abilities, experience with mental health issues, higher academic status and self-reflection were deemed crucial for non-specialists. For instance, a female mental health worker from Colombo highlighted such qualities by exemplifying the need for introspection and bias management in non-specialists:

*“I feel like [non-specialists] also need […] to be introspective and identify their personal biases and deal with them. […] [As psychologists] we have to deal with people with a lot of problems, and most of the time, we deal with people who are on the receiving end of problems, but also, we deal with the person who is the problem sometimes. And that’s very difficult because we have our own triggers, we have our own trauma, we have things that happen to us, and we have to put those aside and not have those biases against our client […]”.*

#### Theme 4: Suggestions to facilitate reach, acceptance and feasibility

Responding to identified concerns, participants from both groups highlighted key factors to consider when implementing any mental health interventions involving non-specialists or specifically for school-based outreach interventions in Sri Lanka.

Important considerations to facilitate the reach of such interventions included effective advertisement, particularly through *“word of mouth” (Mental health worker, woman, Colombo)* or through specific channels, such as social media, but also through “*places of religious importance” (Farmer, man, Badulla)*, or “mothers” *(Mental health worker, woman, Colombo)*, as these institutions or societal figures are respected by the community members. Another example that was highlighted, mainly by community members, was community recruitment, in which participants stressed the need for non-specialists to approach individuals in their communities actively: *“people who want the non-specialists, they are waiting for people to come to them and then they assist them but they don’t reach out by themselves.” (Police officer, men, Badulla).*

Other identified factors focused on enhancing trust and acceptance of non-specialists and addressing the reluctance to seek such interventions due to stigma-related concerns. Therefore, particularly community members emphasised that for people to accept such interventions, the privacy of the service user needs to be warranted. Specific suggestions included enforcing clear instructions to non-specialists on protecting private information or integrating care settings into general healthcare facilities. To reduce stigma-related concerns in school-based interventions, particularly MHC workers suggested avoiding a pure illness-related angle to overcome the associated mental health stigma.

*“I think, when we use the term mental health, it sounds like a more medicalised thing. Whereas I think, going back to things like your rights, and wellbeing, what are the signs to look for? It is mental health but breaking it down into more friendly or less medical-sounding topics. Because if you go to school, and the teachers run a programme about mental health, already, the children are coming with stigma, with taboo, with – because in Sri Lanka, a lot of the language around the colloquial language, around mental health in Sri Lanka is also very damaging.” (Mental health worker, women, Colombo).*

To facilitate the feasibility and uptake of such interventions, participants from both groups, but mostly mental health workers, noted that ensuring fair compensation for non-specialists and providing free or affordable mental health services for users is essential. Also, collaboration with specific organisations would serve as a means to ensure the feasibility of such interventions. Examples included collaborating with and obtaining approval from responsible governmental organisations or widely accepted non-governmental organisations, such as the “WHO” or religious institutions. Moreover, providing means of transportation was indicated as necessary due to the infrastructural barriers. One participant, therefore, suggested that *“In Sri Lanka, midwives are given scooter bikes, like small bikes for them to visit houses. […] if you could organise, then it’ll be very successful. Otherwise, here the transportation is [a] bit difficult in Sri Lanka. […]” (Banker, men, Colombo).* Another important aspect related to considering differences in resource availabilities across the country. In particular, the participants referred to considering differences in schools in the country. Participants explained that large differences may exist between (international) private and government schools or between *popular* and *unpopular* schools regarding the available resources and capacities to integrate such interventions, especially with regard to the workload of teachers.

*“So these popular schools are having children up to 50. […] So because of this high number in the so-called popular school, there may be problems in implementing this kind of programme by the teacher.” (Mental health worker, woman, Colombo).*

With regard to the cultural diversity in Sri Lanka, participants from both groups highlighted the necessity to consider and tailor the interventions to the specific regions in which they should be implemented. Therefore, it seemed essential to consider differences in religious norms (Islamic, Buddhist and Christian); languages (Sinhala, English and Tamil) and other social norms and structures. Another deeply rooted aspect in the Sri Lankan culture also seemed to be the role of the family in mental health. With regard to the implementation of the school-based educational outreach interventions, particularly mental health workers suggested actively engaging parents through, for example, workshops or seminars:

*“But parenting is a very big impact on that. We can’t do one aspect, we have to focus on everything. And the parent’s things and environment are the main major impact on the student’s life and their thinking and their behaviour.” (Mental health worker, woman, Colombo).*

## Ideas for other approaches to reduce the MHC gap

According to the participants, two primary alternative strategies for addressing the MHC gap in light of limited resources became evident: 1) providing outreach in work settings and 2) using media to raise awareness (Supplementary Material S10). Specifically, participants stressed that initiatives to raise awareness and provide education through outreach programmes should not solely target school children but should encompass the entire community. Consequently, the significance of delivering such interventions in workplaces and via various media outlets, including social media, newspapers and teledramas, was recognised.

*“[The participant] […] believes that this [the educational outreach intervention] will definitely help if we take it outside into other institutions [than schools] because there is a lot of mental health issues in Sri Lanka the way that he sees it. […] The implementation of a system like this will assist those people to do their jobs better as well and be healthy.” (Police officer, man, Badulla; translated by the interpreter).*

## Discussion

In this study, focusing on non-specialist mental health interventions in Sri Lanka, four themes emerged: concerns about effectiveness, acceptance and feasibility; perceived benefits of these interventions to bridge the MHC gap; important non-specialist characteristics to improve acceptance and methods to enhance reach, acceptance and feasibility. The interventions’ benefits were linked to increasing mental health intervention access and reducing stigma related to specialist care, though stigma remained a barrier alongside doubts about non-specialist competencies and limited resources. Participants recommended specific traits and occupational backgrounds for non-specialists to boost acceptance and made suggestions to facilitate the interventions’ reach and feasibility.

The general community’s awareness of mental illness and recognition of the MHC gap may aid the implementation of non-specialist strategies. This understanding reflects a sense of readiness, as participants acknowledge both the prevalence of mental illness and the shortcomings of current healthcare solutions. Prior research suggests that community recognition of a problem and the system’s inadequacies partly signify readiness for change and implementation of new approaches (Oetting et al., 1995).

Nevertheless, the fact that participants expressed doubts about non-specialist competencies and, thus, the effectiveness of such interventions may pose a barrier to the implementation, which was also identified in task-sharing interventions in other LMICs (Le et al., 2022). One potential explanation for these concerns among our participants could be their negative experiences with existing non-specialist mental health interventions (Supplementary Material S8). Particularly, the absence of regulations governing the activities of non-specialists raised concerns among our participants, highlighting the need for clear guidelines distinguishing between untrained non-specialists and those who have undergone evidence-based training while being supervised.

To enhance the acceptance of interventions, our participants stressed the careful selection of non-specialists as crucial, focusing on specific occupation groups but also attributes like soft skills and coping abilities. While competencies can be developed through non-specialist programmes like mhGAP (World Health Organization, 2019), personal traits are harder to change. Therefore, appointing non-specialists requires a thorough evaluation of these traits, possibly using tools like the social distance scale or depression attitudes questionnaire (Kohrt et al., 2018).

Moreover, our results indicated that the preferences for the type of task-shifting interventions varied. In rural Sri Lankan areas, collaborative task-sharing involving multiple healthcare workers was often seen as impractical due to workforce shortages, echoing previous research findings (Barnett et al., 2019). Although sole-provider interventions could address these severe scarcities, some participants still favoured a collaborative stepped-care approach, particularly for its specialist or medical doctor involvement. This preference aligns with the notion that trusted healthcare providers can enhance patient experience (Reist et al., 2022). In regions where collaborative approaches are challenging, introducing service users to non-specialists through initial meetings with a supervising specialist or doctor could build trust, with digital technologies aiding remote connections. Moreover, clear communication between non-specialists and users is essential, ensuring patients understand the intervention’s goals and the possibility of specialist referral in both stepped-care and sole-provider models.

Participants also raised concerns about increased stigma and discrimination, which was similarly identified as a significant barrier to implementing task-sharing interventions in other LMICs (Le et al., 2022). Generally, such entrenched stigmatising beliefs represent complex challenges demanding a cultural shift in attitudes, as noted in previous research (Knaak et al., 2017) and stressed by many participants, identifying the need to expand mental health awareness and education as a key factor in reducing the MHC gap. At the intervention level, the mental health worker respondents suggested a proactive approach to counteracting stigma by avoiding an illness-focused angle. They proposed leveraging positive psychology as the foundation of educational programmes, a method previously demonstrated to effectively enhance the well-being of university students (Hobbs et al., 2022). To tackle stigma-related issues further, participants emphasised the active involvement of key community members or family members, a method already proven beneficial in other LMICs (Le et al., 2022).

### Strengths and limitations

Several limitations warrant consideration. First, it needs to be acknowledged that 22 individuals declined to participate after initial contact with the research team. Notably, the political unrest in Sri Lanka could have likely reduced participation, with many potential participants withdrawing without explanation. Moreover, this unrest necessitated digital interviews instead of field data collection, potentially introducing a selection bias by excluding those without digital access and thus potentially affecting the socio-economic diversity in the sample. Additionally, reliance on convenience sampling might have influenced the results, though this was partly offset by using various recruitment methods, including distributing leaflets. Technical issues during interviews and the need for an interpreter might have caused information loss, although the interpreter’s expertise and the interviewer’s language skills minimised this risk. Moreover, it should be noted that our participants were recruited from only two districts in Sri Lanka, potentially limiting the generalisability of our results to the entire country. However, we sought to mitigate this limitation by deliberately choosing two districts with differing economic levels, and thus allowing for more heterogeneous insights. A notable limitation of our study is the gender imbalance within the participant groups: the majority of the MHC workers were female, whereas the majority of community members were male, based on the researchers’ classification using cisgender identities. This disparity may have influenced the perceptions of significant barriers and facilitators reported by each group. Finally, we were unable to conduct member validation with the final results.

Despite these challenges, the study has several strengths. First, it proceeded successfully amidst difficult conditions, indicating the topic’s relevance and public interest. Moreover, a significant strength of this study is that we conducted interviews with 20 participants, including MHC workers from various specialties (such as researchers and clinical psychologists) and diverse community members who spoke openly about a highly stigmatised topic. Furthermore, we ensured a high degree of data saturation by continuing data collection until no new insights emerged. Finally, this study is a pioneering effort in Sri Lanka to explore barriers and facilitators to non-specialist interventions, engaging mental health workers and community members.

## Conclusion

In summary, our study provides crucial insights for policymakers and researchers on implementing non-specialist mental health interventions in Sri Lanka, highlighting that while many factors may apply to other LMICs as well, some are uniquely pertinent to the Sri Lankan context. The findings indicate a generally positive perception among the Sri Lankan community, including mental health experts, about the role of non-specialists in reducing the MHC gap by providing more approachable and affordable care compared to specialists. However, potential challenges include the population’s scepticism about the effectiveness of non-specialists, specifically in treatment roles and competencies and concerns over resource availability for interventions involving multiple providers or digital technologies. Another significant challenge is the stigma associated with mental health, which could hinder both the delivery and acceptance of these interventions. A thorough selection of non-specialists from trusted groups, such as religious leaders, midwives, social workers, female police officers or teachers and ensuring suitable personal characteristics (i.e., through specific assessment tools) could enhance trust and acceptance. Introducing non-specialists through specialists or general practitioners, possibly remotely, may also build trust. Moreover, the involvement of key community figures in implementation, like using religious leaders or established organisations for promotion, as well as the active involvement of family members in such interventions, could aid the process. Additionally, clear privacy regulations and stigma reduction strategies, such as integrating mental health services into general practice and focusing on positive psychology in education, seem crucial. Furthermore, fair payment regulations and offering means of transportation seem imperative to enhance non-specialist retention and service user engagement in such interventions. Intervention implementers must also consider Sri Lanka’s multicultural context, ensuring interventions are culturally sensitive and tailored to specific regions regarding language, cultural norms and resources. While unforeseen factors related to specific interventions or contexts might arise, these recommendations are poised to proactively address many of the identified challenges. Finally, this study highlights that besides non-specialist mental health interventions, a key strategy to lessen the MHC gap in Sri Lanka involves enhancing mental health education and awareness through workplace programmes and (social) media channels.

## Supporting information

Wijekoon Mudiyanselage et al. supplementary material Wijekoon Mudiyanselage et al. supplementary material

## Data Availability

The data that support the findings of this study are available on request from the corresponding author (K.W.W.M.). The data are not publicly available due to the personal nature of these data (despite anonymisation) but are available from the corresponding author on reasonable request.

## Supplementary material

The supplementary material for this article can be found at http://doi.org/10.1017/gmh.2024.75.
