# Supplementary material for: Identifying contextual barriers and facilitators in implementing non-specialist interventions for mental health in Sri Lanka: A qualitative study with mental health workers and community members
Source: Glob Ment Health (Camb). 2024 Oct 8;11:e76. doi: 10.1017/gmh.2024.75 (PMC11504943; doi:10.1017/gmh.2024.75)
Supplement: Wijekoon Mudiyanselage et al. supplementary material [file S205442512400075Xsup001.zip › Additional file 1 Examples of decentralisation efforts in Sri Lanka.docx]

# Additional file 1: Summary of Sri Lankas healthcare system and examples on current decentralisation efforts in Sri Lanka

# Sri Lanka’s healthcare system

Sri Lanka’s healthcare system is regarded as exemplary among LMICs due to its pronounced focus on public health and prevention. This focus is evident in the division of healthcare workers into two primary tiers: one dedicated to prevention and the other to curative services (Jenkins and Cooray 2012; Minas et al. 2017). The majority of interventions for prevention and treatment, including mental health services, are provided free of charge at the point of delivery. Although the public sector delivers most health services, there is an expanding private sector. However, access to these private services is limited to a small segment of the population who can afford the associated costs (Rajapaksa L et al. 2021; World Health Organization 2022). Currently, Sri Lanka lacks a gatekeeping system, allowing unrestricted access to primary, secondary, and tertiary care centres. This absence of gatekeeping has resulted in an overload, particularly in specialised care settings, leading to extended waiting lists (Rajapaksa L et al. 2021).

Mental health services in Sri Lanka, encompassing both preventive and curative aspects, are provided by a combination of public and private institutions, as well as non-governmental organisations (NGOs). Despite this diverse delivery system, the government is the primary source of funding. However, the mental health budget is not allocated a distinct line or heading, highlighting an absence of dedicated funding within the overall health budget (Kitsiri 2015; World Health Organization 2022).

# Current efforts in decentralising the mental healthcare system

**Training for medical doctors:**

Medical Officers with a Diploma in Psychiatry (MOPsyc) and Medical Officers of Mental Health (MOMH), who are medical graduates with either a one-year diploma or a one-month certificate in psychiatry, respectively (Minas et al. 2017). These professionals are primarily positioned in district and base hospitals to provide outpatient care, domiciliary care, and mental health promotion in schools (Fernando et al. 2017). Apart from that, since 2018, psychiatry has been included as a final subject in the undergraduate medical curriculum, enabling general medical doctors to identify mental illnesses, provide referrals, and offer basic mental health treatment (Hapangama et al. 2023; Kathriarachchi et al. 2019).

**Medical nurses and social workers**

In 2010, a six-month specialisation program was introduced for medical nurses to become psychiatric nurses, focusing on community-based care, follow-up, and treatment retention during home visits (Fernando et al. 2017). Additionally, the cadre of psychiatric social workers was introduced whose main tasks include assessing and addressing the psychological and social needs of community members (Fernando et al. 2017).

# References

**Fernando N, Suveendran T and Silva de C** (2017) Decentralizing provision of mental health care in Sri Lanka. WHO South- East Asia J Public Health 2017;6:18-21. *WHO South-East Asia Journal of Public Health* **6**(1)**,** 18-21. <https://doi.org/10.4103/2224-3151.206159>.

**Hapangama A, Kuruppuarachchi K and Hanwella R** (2023) Achievements and challenges in psychiatric education and training in Sri Lanka. *British Journal of Psychiatry* **20**(1)**,** 2-4. <https://doi.org/10.1192/bji.2021.35>.

**Jenkins R and Cooray M** (2012) Integration of mental health into primary care in Sri Lanka. *Mental Health in Family Medicine* **9**(1)**,** 15-24, <https://www.ncbi.nlm.nih.gov/pmc/articles/PMC3487606/>.

**Kathriarachchi S, Seneviratne V and Amarakoon L** (2019) Development of mental health care in Sri Lanka: Lessons learned. *Taiwanese Journal of Psychiatry* **33,** 55-65. <https://doi.org/10.4103/tpsy.tpsy_15_19>.

**Kitsiri R** (2015) Policy Study Report - 2009 Financing Mental Health Care in Sri Lanka. Available at <https://www.researchgate.net/publication/284732419_Financing_Mental_Healthcare_in_Sri_Lanka> (accessed 23.05.2024).

**Minas H, Mendis J and Hall T** (2017) Mental Health System Development in Sri Lanka. In Minas H and Lewis M (eds), *Mental Health in Asia and the Pacific.Internatiuonal and cultural Psychology.* Boston, MA: Springer, 59-77.<https://link.springer.com/chapter/10.1007/978-1-4899-7999-5_4#citeas>

**Rajapaksa L, De Silva P, Abeykoon A, Somatunga L, Sathasivam S and S P** (2021) Sri Lanka health system review. New Delhi: World Health Organization Regional Office for South-East Asia. Available at <https://apo.who.int/publications/i/item/sri-lanka-health-system-review> (accessed 23.02.2024).

**World Health Organization** (2022) Mental Health Atlas 2020 Country Profile: Sri Lanka. Available at <https://www.who.int/publications/m/item/mental-health-atlas-lka-2020-country-profile> (accessed 19.03.2024).
