# Supplementary material for: Identifying contextual barriers and facilitators in implementing non-specialist interventions for mental health in Sri Lanka: A qualitative study with mental health workers and community members
Source: Glob Ment Health (Camb). 2024 Oct 8;11:e76. doi: 10.1017/gmh.2024.75 (PMC11504943; doi:10.1017/gmh.2024.75)
Supplement: Wijekoon Mudiyanselage et al. supplementary material [file S205442512400075Xsup001.zip › Additional file 3 Topic guides and case examples.docx]

# Additional file 3: Topic guides and case examples

## Topic guides

Introduction (5 min):

- Thank participant
- Introduce self
- Introduce topic:

Only for community members: The purpose of this interview is to understand what we could do to help people with health problems in Sri Lanka. In particular, we will discuss three different strategies used in other countries. We want to know from you what you think about these strategies and if they would work in the context of Sri Lanka or not. If you think these strategies are helpful, we would like to know what we need to consider when implementing this strategy in Sri Lanka.

Only for mental health workers: Non-specialists are laypeople who did not receive extensive mental health education. This definition excludes mental health workers, such as psychologists, psychiatrists etc. Non-specialists usually receive a short-term training to deliver a mental health intervention. This could include mental health promotion activities, illness prevention or treatment activities. We want to know what you think about this strategy and what factors we would need to consider when implementing this intervention in the Sri Lankan context. As a basis for discussion, we will use three existing non-specialist strategies.

- Before we start, a short reminder: this interview will be audio-recorded. As stated on the consent form, you have the right to stop the interview at any point, if you feel like it, without stating any reason.
- If you have any question, please let us know now, otherwise we will start.

**Q1.Thank you again xxx. At the start, I was wondering whether you could tell me how you have been living in xxx? What occupation do you work in right now? Thank you.**

**Only for community members: Now I will introduce you to two people, Aavi and Venuri.**

*(show graphic A)*

About Venuri:

Venuri is a 25-year-old woman from Colombo who studies at the university. She has not been looking good for many weeks now. She is always sad, feels miserable, and cannot cope with everyday situations. It is very hard to find joy in her life, and she has difficulties keeping up with her work. That is why she had to quit her studies. Also, she usually stays in bed for a very long time, and she cannot eat properly because she has no appetite. Sometimes she feels so hopeless that she thinks of ending her life.

About Aavi:

Aavi is 22-year-old men from Bandarawela. His friends and family have noticed that he started to act different lately. He always talks about seeing things, that are not actually there. Also, he has become extremely restless and frightened, although there is no reason for that. He reports hearing voices in his head, that no one else can hear. These voices command him to do dangerous things, like standing on the railway. Additionally, he has extreme mood swings, changing very quickly from being extremely angry to extremely confident and happy, thinking he would be a king.

**Only for community members: How do you think could Aavi and Venuri improve their current life?**

*Probes:*

- *Is there a way for Aavi and Venuri to get better?*
- *Where could they go to get help?*

**Only for community members: Explain non-specialist approach:**

*To overcome the problems that Aavi and Venuri currently suffer from, they could regularly visit specialised people like psychologists or psychiatrists. These specialised people who have studied the problems that Aavi and Venuri face for many years will use different methods that they have learned in university to help them. Sometimes they prescribe medication to help them. However, the general problem now is that there are not enough specialised people in Sri Lanka who could help them. To overcome this problem, the idea is that the few available specialised people could train and supervise people from the community in helping people like Venuri and Aavi. This method is called “the non-specialist approach”.*

**Q4. Are you aware of any non-specialist approaches in Sri Lanka?**

*Probes:*

- *What do they aim for? Which non-specialist is used? In what setting do they take place?*
- *What are the benefits of these existing programs?*
- *What are the limitations of these existing programs?*

**Q5. Researchers have tested an intervention in Cambodia in which teachers receive training by specialists and educate their students on the topic of mental health. They can also identify students with problems and refer them to specialists, if needed.** **What are your thoughts about this intervention?** *(show graphic B1-B2)*

- *How could this strategy help people like Aavi and Venuri?*
- *Do you think that Aavi and Venuri would not have such problem, if they would have had learned about these things in school?*
- *Do you think that Aavi and Venuri would know how to get better, if they have had learned about these things in school?*

**Q6.** **Imagine there is a research team that thinks about implementing this intervention in Sri Lanka. However, they are not from Sri Lanka, and they do not know how to set up this intervention so that it is acceptable and sustainable in the Sri Lankan context. That is why they ask you to be their local consultant. According to your view, what contextual factors do they need to consider when implementing this intervention in Sri Lankan schools?**

*Probes*

- *Do you think children/young people like/enjoy such classes? Why or why not?*
- *Do you think many parents of children would accept that their children have such classes? Why or why not?*
- *Do you think that there are many teachers that would accept to provide these classes? Why or why not?*

**Q7. What role can technology play in this intervention?**

*Probes:*

- *(How) can technology be used?*
- *How would the use of technology in such interventions be perceived?*

**Q8. Could there be a better alternative to promote mental health/prevent mental illness in Sri Lanka? What and why?**

*Probes:*

- *Are there other settings where educational work on mental health should take place? If so, where and why?*
- *Are there existing programs that would be better in promoting mental health than the proposed intervention? If so, which and why?*

**Q9. In India, researchers have tested a so called “stepped care approach”. In this intervention, a non-specialist is trained to provide low-level treatment (psychoeducation, breathing exercises and if necessary, medication). If this treatment does not help, the patient will be referred to a psychiatrist. What are your thoughts about this intervention?** *(show graphic C1-C2)*

*Probes*

- *Do you think this scenario would be acceptable in Sri Lanka?*
- *Is it helpful to make sure that everyone with a mental disorder can receive the appropriate treatment that they need? Why or why not?*

**Q10. Imagine there is a research team thinks about implementing this intervention. However, they are not from Sri Lanka, and they do not know how to set up this intervention so that it is acceptable and sustainable in the Sri Lankan context. That is why they ask you to be their local consultant. According to your view, what contextual factors do they need to consider when implementing this intervention in Sri Lankan?**

*Probes*

- *How should such an intervention be implemented in SL: which type of non-specialist, which setting?*
- *What are the anticipated difficulties in implementing this intervention on the long-term?*
- *What would be needed to overcome these difficulties?*
- *What other factors could help to implement this intervention?*

**Q11. What role can technology play in this intervention?**

*Probes:*

- *How can technology be used*
- *How would the use of technology in such interventions be perceived?*

**Q12 In Pakistan, researchers have tested an intervention in which one non-specialist receives training and is supervised by a specialist to provide care. What are your thoughts about this intervention?** *(show graphic D1-D2)*

*Probes:*

- *Would this intervention be acceptable to people in Sri Lanka?*
- *Is it helpful to make sure that everyone with a mental disorder can receive the appropriate treatment that they need? Why, why not?*

**Q13. Imagine there is a research team that thinks about implementing this intervention. However, they are not from Sri Lanka, and they do not know how to set up this intervention so that it is acceptable and sustainable in the Sri Lankan context. That is why they ask you to be their local consultant. According to your view, what contextual factors do they need to consider when implementing this intervention in Sri Lankan?**

*Probes*

- *How should such an intervention be implemented in SL: which type of non-specialist, which setting?*
- *What are the anticipated difficulties in implementing this intervention on the long-term?*
- *What would be needed to overcome these difficulties (mentioned above)*
- *What other factors could help to implement this intervention?*

**Q14. What role can technology play in this intervention?**

*Probes:*

- *How can technology be used?*
- *How would the use of technology in such interventions be perceived?*

**Q15. Do you know/Are you aware of a better alternative to treat mental illness in SL, given the scarcity of specialists in Sri Lanka?**

- *What other approaches could be better in helping people with mental disorders in Sri Lanka?*
- *Are there existing programs from other countries, that would be more suitable in treating mental illness than the proposed intervention (without using specialists)? If so, which and why?*

Conclusion (5 min)

● Thank participant


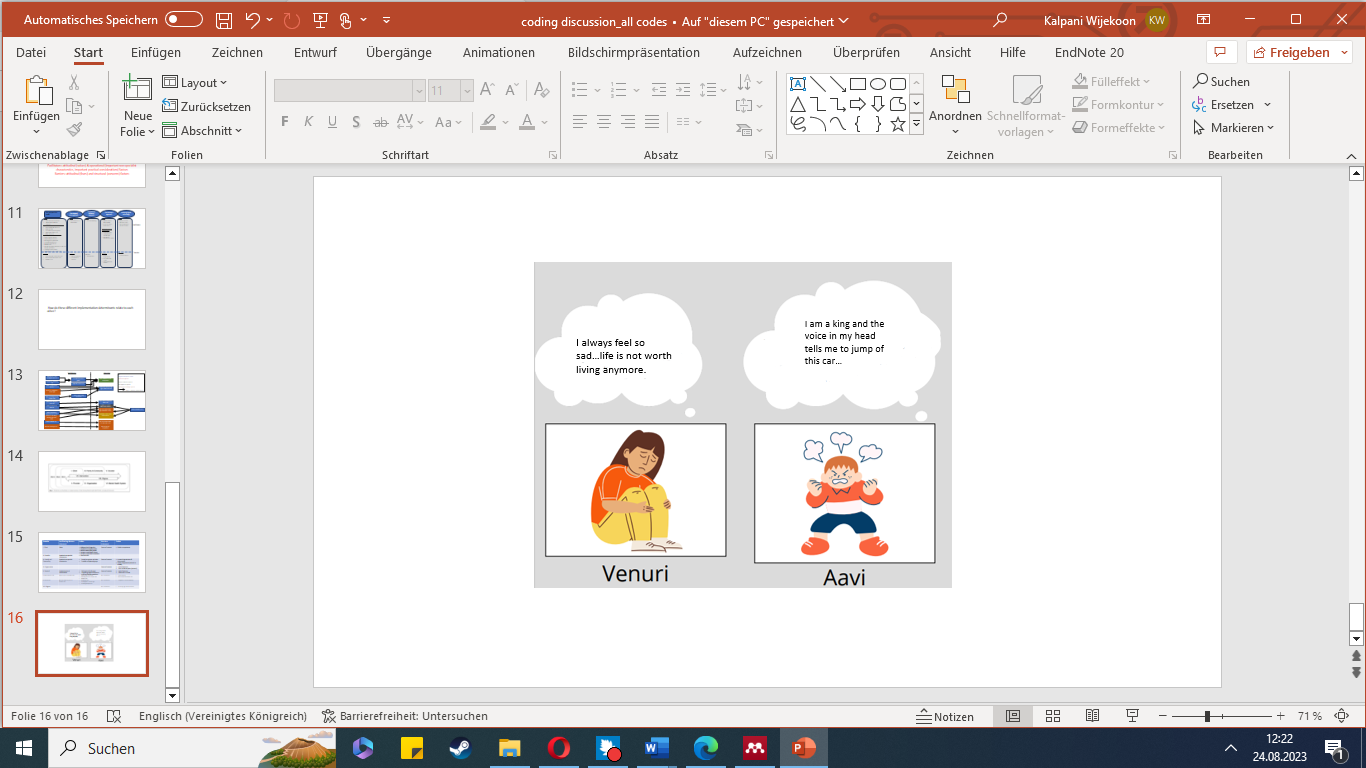

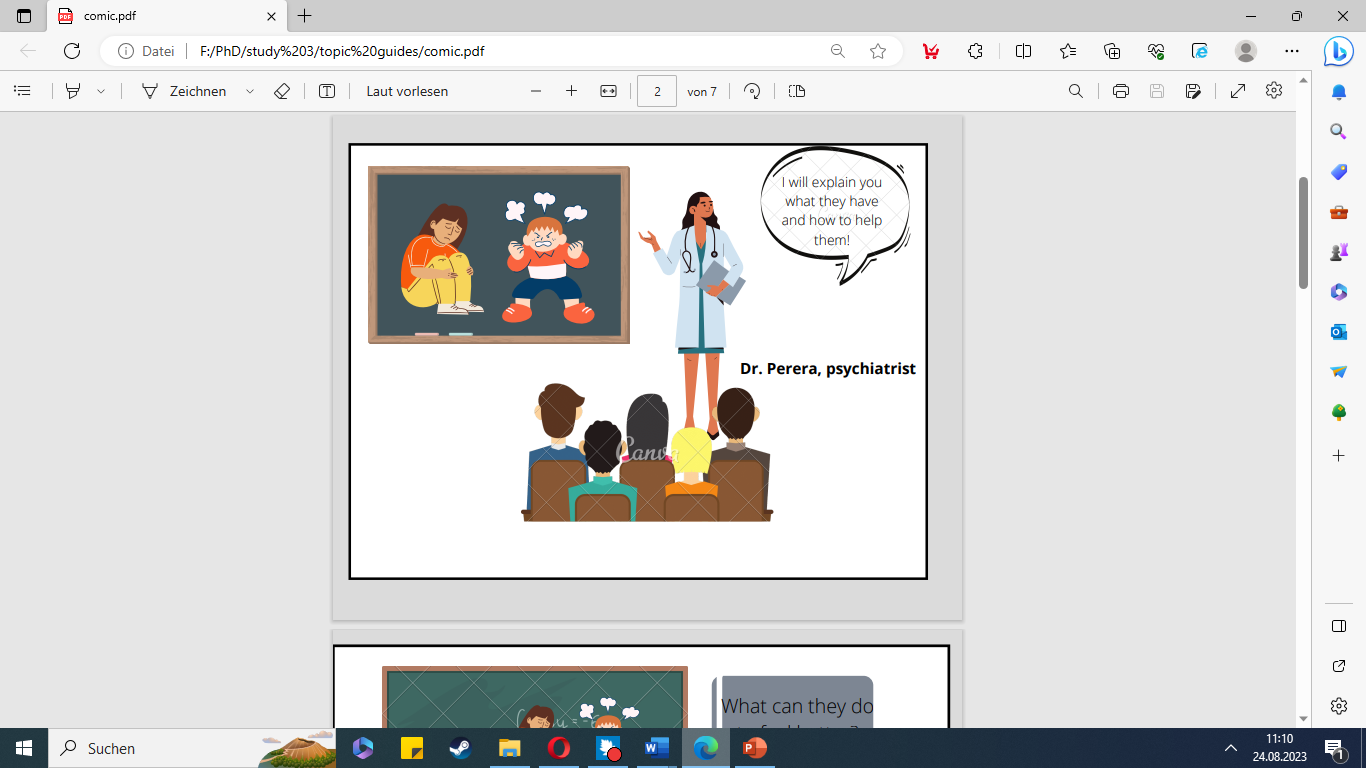

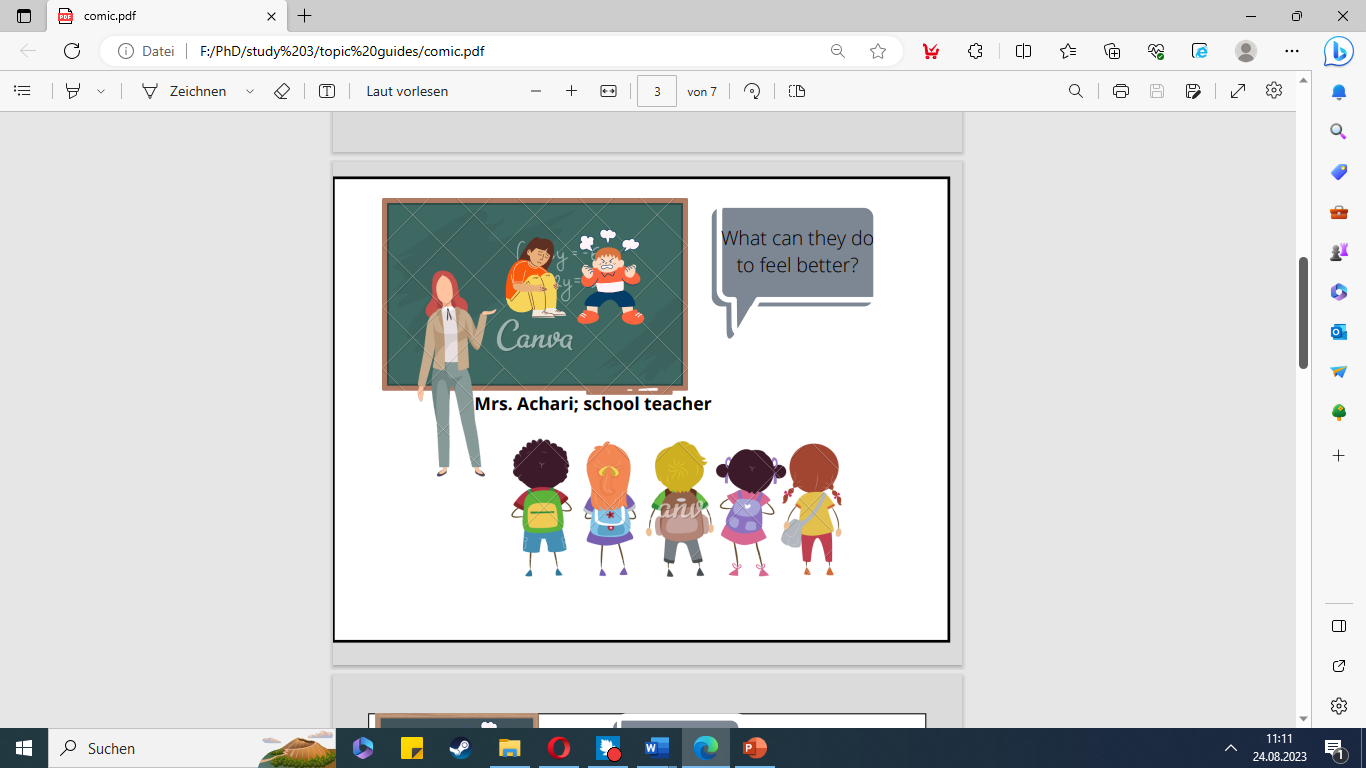


Graphic A Graphic B1 Graphic B2


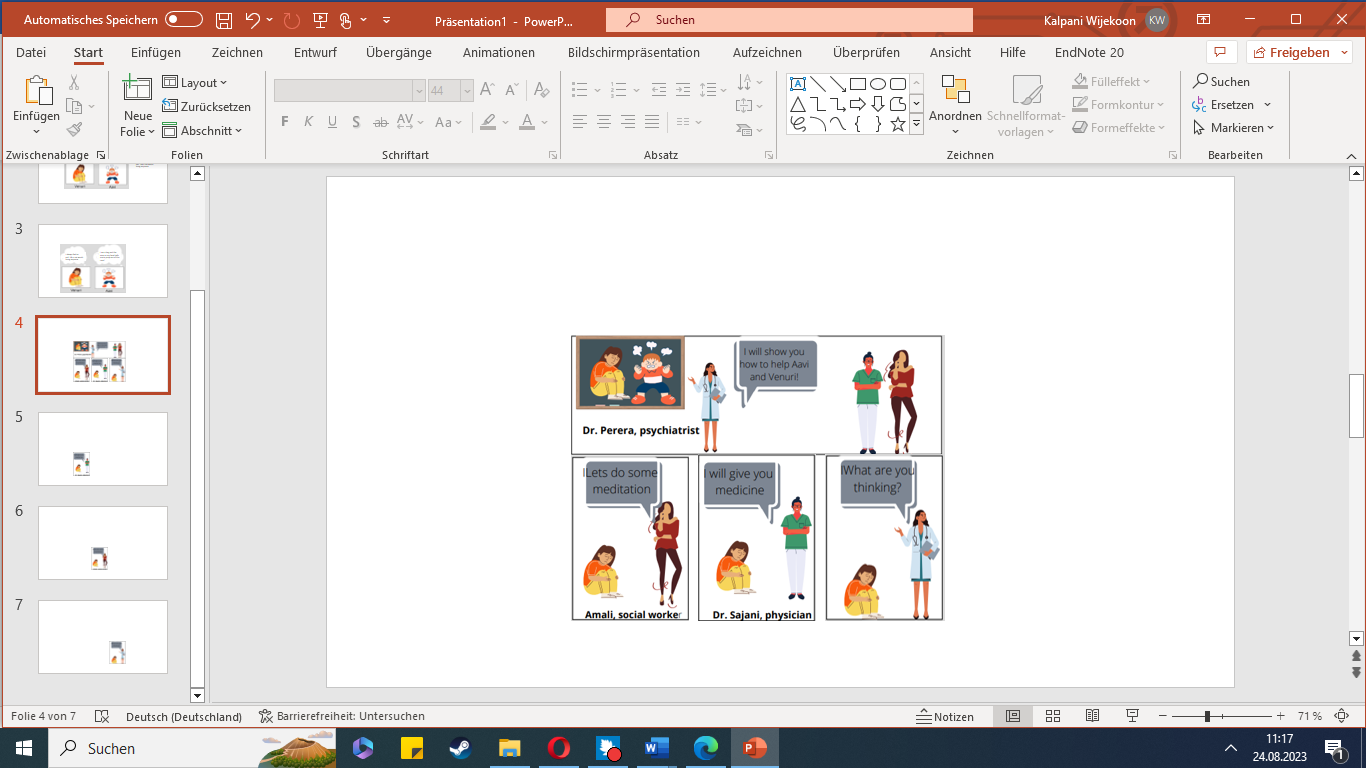

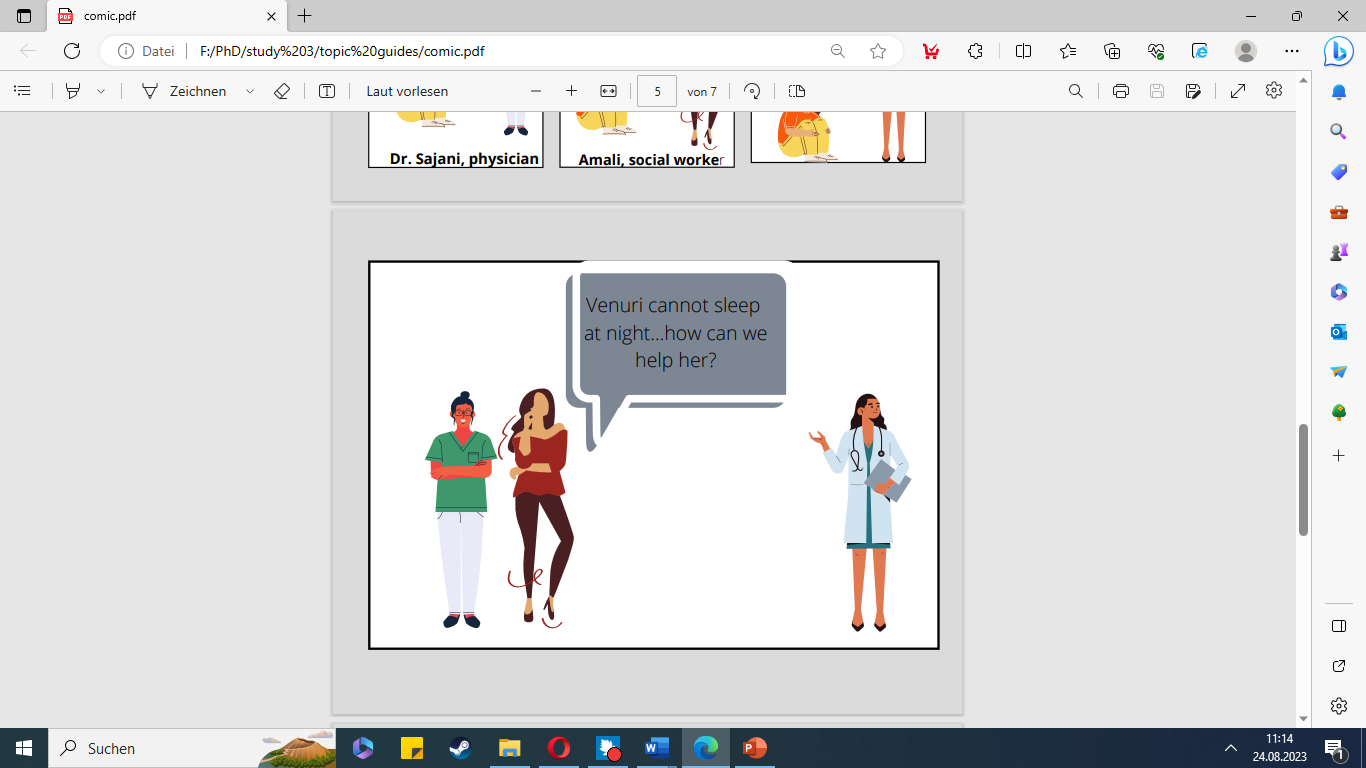

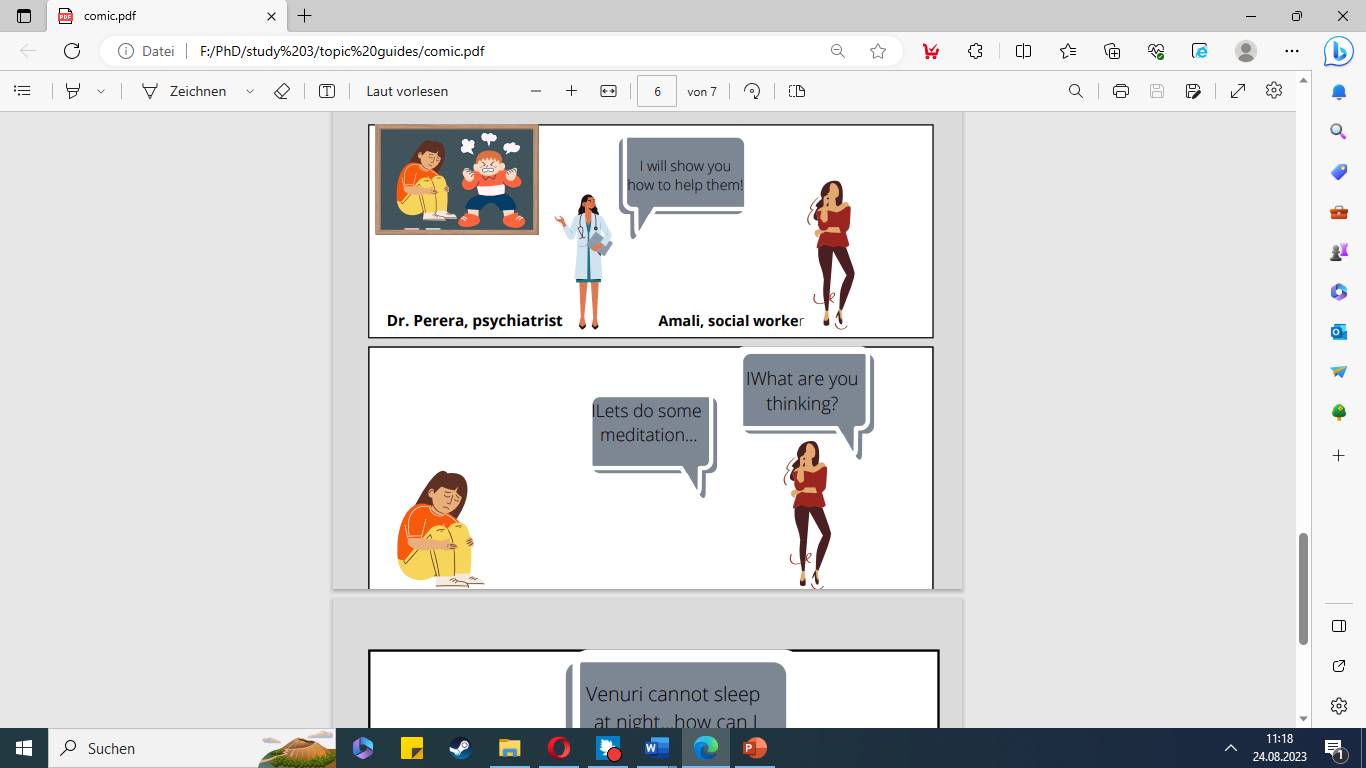


Graphic C1 Graphic C2 Graphic D1


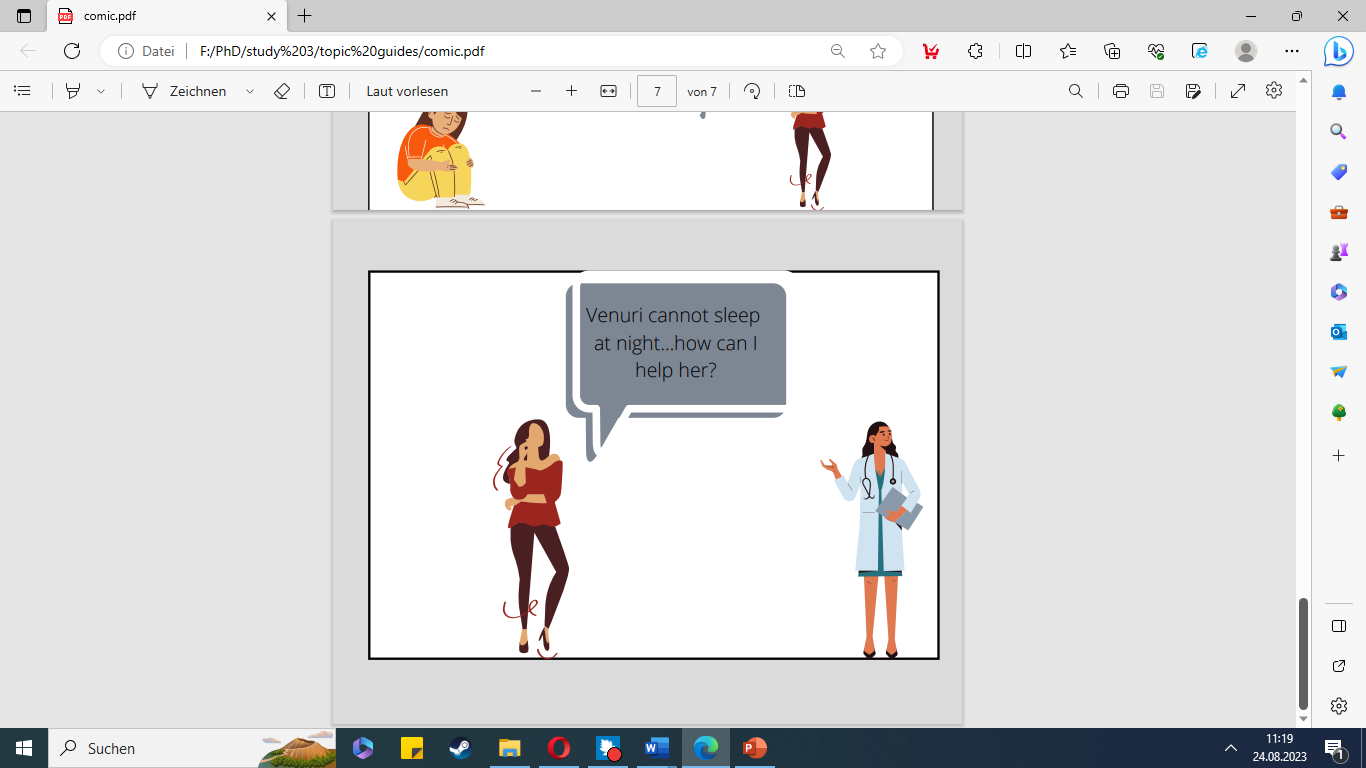


Graphic D2

## Case examples

Short summary of the different non-specialist approaches:

1. The outreach approach: We presented a commonly used outreach intervention in schools based on a previously conducted study in Cambodia (Phoeun et al. 2019). In this intervention, teachers received short-term training to provide mental health training to their students. Additionally, they are able to identify students with mental illness problems and refer them to specialists if needed.
2. The task-sharing approaches: In task-sharing approaches, non-specialists are directly involved in care delivery. In all cases, the non-specialist is trained and supervised by specialists. There are two types of care delivery depending on the involvement of the specialist in care delivery:
   1. The stepped-care approach: We presented an example based on a study conducted in India (Patel et al. 2010). In the first instance, the non-specialists provides the least intensive care in form of psychosocial care components and another, general practitioner non-specialist can provide medication if needed. If this care does not reduce symptoms or in case of relpase, the patient is referred to a specialist who provides more intensive care. Additionally, patients who did not respond to the prior treatment steps and those considered highly suicidal at any point will be referred to a specialist.
   2. The sole-deliverer approach: We presented an example based on a study conducted in Pakistan (Sabir Ali et al. 2003). In this intervention, only one non-specialist provides treatment (supportive, cognitive, and problem-solving sessions) to people with mental illness problems. In case of adverse effects the patients will also be referred to a specialist.

## References

**Patel V, Weiss H, Chowdhary N, Naik S, Pednekar S, Chatterjee S, De Silva M, Bhat B, Araya R, King M, Simon G, Verdeli H and Kirkwood B** (2010) The effectiveness of a lay health worker led intervention for depressive and anxiety disorders in primary care: the MANAS cluster randomized trial in Goa, India. *Lancet* **376**(9758)**,** 2086-2095. <https://doi.org/10.1016/S0140-6736(10)61508-5.The>.

**Phoeun B, Nguyen AJ, Dang MH, Tran NT and Weiss B** (2019) Assessing the Effectiveness of Teachers’ Mental Health Literacy Training in Cambodia: A Randomized Controlled Trial. *VNU Journal of Science: Education Research* **35**. <https://doi.org/10.25073/2588-1159/vnuer.4279>.

**Sabir Ali B, Rahbar MH, Naeem S, Gul A, Mubeen S and Iqbal A** (2003) The effectiveness of counseling on anxiety and depression by minimally trained counselors: A randomized controlled trial. *American Journal of psychotherapy* **57**(3)**,** 324-326. <https://doi.org/10.1176/appi.psychotherapy.2003.57.3.324>.
