# Supplementary material for: Identifying contextual barriers and facilitators in implementing non-specialist interventions for mental health in Sri Lanka: A qualitative study with mental health workers and community members
Source: Glob Ment Health (Camb). 2024 Oct 8;11:e76. doi: 10.1017/gmh.2024.75 (PMC11504943; doi:10.1017/gmh.2024.75)
Supplement: Wijekoon Mudiyanselage et al. supplementary material [file S205442512400075Xsup001.zip › Additional file 4 Research team characteristics and reflexivity.docx]

# Additional file 4: Reflexivity

**Credentials**

KWWM: B.A., M.Sc.; MSDM: B.Sc Psychology; HB: B.Sc, M.Sc., PhD; DF: B.Sc., M.Sc., PhD, Prof.; FJ: B.Sc., M.Sc., PhD

**Occupation during time of study**

KWWM: Public Health Researcher, PhD student; MSDM: Psychology student; HB: Public Health Researcher, DF: Public Health researcher; FJ: Mental Health Researcher

**Gender**

KWWM: female; MSDM: female; HB: female, DF: female, FJ: female

**Interviewer characteristics**

KWWM received training in qualitative research and has experience in, among others, conducting and publishing qualitative research. Moreover, this study is part of her dissertation. MSDM has experience in Psychology, and has already conducted a qualitative study in the form of her Bachelor thesis.

**Relationship with participants**

KWWM: none; MSDM: knew some of the mental health workers, but did not attend any of the interviews with them, and did not perform coding for any of their transcripts; HB: none; DF: none; FJ: none

**Knowledge of participants**

Some of the participants knew that this research was part of the primary researchers PhD work. Additionally, participants were briefed on the study purpose and procedures. Two mental health workers requested to read the articles based on which we discussed the non-specialist interventions (see additional file 2).
