# Supplementary material for: Identifying contextual barriers and facilitators in implementing non-specialist interventions for mental health in Sri Lanka: A qualitative study with mental health workers and community members
Source: Glob Ment Health (Camb). 2024 Oct 8;11:e76. doi: 10.1017/gmh.2024.75 (PMC11504943; doi:10.1017/gmh.2024.75)
Supplement: Wijekoon Mudiyanselage et al. supplementary material [file S205442512400075Xsup001.zip › Additional file 5 COREQ checklist.docx]

# Additional file 5: Consolidated criteria for reporting qualitative studies (COREQ): 32-item checklist

| **No. Item** | **Guide questions/description** | **Reported on Page #** |
| --- | --- | --- |
| **Domain 1: Research team and reﬂexivity** |  |  |
| *Personal Characteristics* |  |  |
| 1. Interviewer/facilitator | Which author/s conducted the interview or focus group? | Page 10 (Line 244-245) |
| 2. Credentials | What were the researcher’s credentials? E.g. PhD, MD | Additional file 4 |
| 3. Occupation | What was their occupation at the time of the study? | Additional file 4 |
| 4. Gender | Was the researcher male or female? | Additional file 4 |
| 5. Experience and training | What experience or training did the researcher have? | Additional file 4 |
| *Relationship with participants* |  |  |
| 6. Relationship established | Was a relationship established prior to study commencement? | Additional file 4 |
| 7. Participant knowledge of the interviewer | What did the participants know about the researcher? e.g. personal goals, reasons for doing the research | Additional file 4 |
| 8. Interviewer characteristics | What characteristics were reported about the inter viewer/facilitator? e.g. Bias, assumptions, reasons and interests in the research topic | Additional file 4 |

| Domain 2: study design |  |  |
| --- | --- | --- |
| *Theoretical framework* |  |  |
| 9. Methodological orientation and Theory | What methodological orientation was stated to underpin the study? e.g. grounded theory, discourse analysis, ethnography, phenomenology, content analysis | p. 11, l.276-278 |
| *Participant selection* |  |  |
| 10. Sampling | How were participants selected? e.g. purposive, convenience, consecutive, snowball | p. 9-10, ll. 233-237 |
| 11. Method of approach | How were participants approached? e.g. face-to-face, telephone, mail, email | p. 9-10 ll-233-237 |
| 12. Sample size | How many participants were in the study? | p. 12, l.295 |
| 13. Non-participation | How many people refused to participate or dropped out? Reasons? | p.10, ll. 240-243; additional file 2 |
| *Setting* |  |  |
| 14. Setting of data collection | Where was the data collected? e.g. home, clinic, workplace | p. 10, l.244 |
| 15. Presence of non-participants | Was anyone else present besides the participants and researchers? | p.10, ll.244-245 |
| 16. Description of sample | What are the important characteristics of the sample? e.g. demographic data, date | p.12 l. 295-297 |
| *Data collection* |  |  |
| 17. Interview guide | Were questions, prompts, guides provided by the authors? Was it pilot tested? | Find both semi-structured interview guides in additional file 3; pilot tested: see p.10, ll. 246-249 |
| 18. Repeat interviews | Were repeat interviews carried out? If yes, how many? | Not feasible |
| 19. Audio/visual recording | Did the research use audio or visual recording to collect the data? | Audio recording, p.10, l. 246 |
| 20. Field notes | Were ﬁeld notes made during and/or after the interview or focus group? | Yes, p. 11, ll.267-268 |
| 21. Duration | What was the duration of the interviews or focus group? | p. 10, l. 246 |
| 22. Data saturation | Was data saturation discussed? | p. 11, ll. 168-169 |
| 23. Transcripts returned | Were transcripts returned to participants for comment and/or correction? | Not feasible |
| Domain 3: analysis and ﬁndings |  |  |
| *Data analysis* |  |  |
| 24. Number of data coders | How many data coders coded the data? | 2 coders for six transcripts (see Fig. 1) |
| 25. Description of the coding tree | Did authors provide a description of the coding tree? | Table 1, additional files 7-10 |
| 26. Derivation of themes | Were themes identiﬁed in advance or derived from the data? | Derived from the data; p. 11, ll.176-181 |
| 27. Software | What software, if applicable, was used to manage the data? | MAXQDA, p.12, l-290 |
| 28. Participant checking | Did participants provide feedback on the ﬁndings? | Not feasible |
| *Reporting* |  |  |
| 29. Quotations presented | Were participant quotations presented to illustrate the themes/ﬁndings? Was each quotation identiﬁed? e.g. participant number | Additional file 7-10; results p.12-19 |
| 30. Data and ﬁndings consistent | Was there consistency between the data presented and the ﬁndings? | Yes (see additional files 7-10) and results section |
| 31. Clarity of major themes | Were major themes clearly presented in the ﬁndings? | Yes, p. 12-13, l.312-318 (each theme is described in detail within the results section and in additional file 9) |
| 32. Clarity of minor themes | Is there a description of diverse cases or discussion of minor themes? | Not applicable |
| **Notes:** This checklist was derived from *Tong A, Sainsbury P, Craig J. Consolidated criteria for reporting qualitative research (COREQ): a 32-item checklist for interviews and focus groups. International Journal for Quality in Health Care. 2007. Volume 19, Number 6: pp. 349 – 357* | | |
