# Supplementary material for: Identifying contextual barriers and facilitators in implementing non-specialist interventions for mental health in Sri Lanka: A qualitative study with mental health workers and community members
Source: Glob Ment Health (Camb). 2024 Oct 8;11:e76. doi: 10.1017/gmh.2024.75 (PMC11504943; doi:10.1017/gmh.2024.75)
Supplement: Wijekoon Mudiyanselage et al. supplementary material [file S205442512400075Xsup001.zip › Additional file 6 Participant characteristics.docx]

# Additional file 6: Participant characteristics

| **Table A5. Demographic sample descriptions** | |
| --- | --- |
| ***Community members (n=11)*** | |
| *Gender^1^* | |
| Female | 36% (n=4) |
| Male | 64% (n=7) |
| *Residency* |  |
| Badulla | 64% (n=7) |
| Colombo | 36% (n=4) |
| *Occupations^2^* | |
| Banker | 18 % (n=2) |
| Teacher and/or school principal | 27% (n=3) |
| Anglican priest | 9% (n=1) |
| IT worker | 9% (n=1) |
| Freelance writer | 9% (n=1) |
| Midwife | 9% (n=1) |
| Police officer | 9% (n=1) |
| Farmer | 9% (n=1) |
| Nanny and household keeper | 9% (n=1) |
| Duration worked in that occupation | 3 months – 32 years |
| ***Mental health workers (n=9)*** | |
| *Gender^1^* | |
| Female | 78% (n=7) |
| Male | 22% (n=3) |
| *Residency* | |
| Badulla | 22% (n=3) |
| Colombo | 78% (n=7) |
| *Occupations^2^* | |
| Clinical psychologist | 44% (n=4) |
| Psychology students | 22% (n=2) |
| Counsellor | 11 % (n=1) |
| Mental health life coach | 11% (n=1) |
| Medical officer of psychiatry | 11% (n=1) |
| Medical officer planner | 11% (n=1) |
| Mental health researcher | 11% (n=1) |
| Duration worked within that occupation (range) | 1 ½ -10 years |
| Notes: ^1^ This assessment is based on assumptions made by the research differentiating only cisgender identities.  ^2^ Percentages for occupations do not add up to 100% given some participants working in more than one occupational field. | |
