# Supplementary material for: Identifying contextual barriers and facilitators in implementing non-specialist interventions for mental health in Sri Lanka: A qualitative study with mental health workers and community members
Source: Glob Ment Health (Camb). 2024 Oct 8;11:e76. doi: 10.1017/gmh.2024.75 (PMC11504943; doi:10.1017/gmh.2024.75)
Supplement: Wijekoon Mudiyanselage et al. supplementary material [file S205442512400075Xsup001.zip › Additional file 7 Mental health beliefs among community members.docx]

# Additional file 7: Coding framework for mental health beliefs of community members

**Understanding the context: What are the mental health beliefs of community members?**

**Summary:**

In our study, most participants (n=5) perceived the symptoms described in the case vignettes as forms of mental burden (code 1.1). Some associated these symptoms with work-related stressors (code 1.2), such as the heightened academic pressures in Sri Lanka, while others linked them to family-related issues (code 1.3), such as a disrupted family background. Participants also noted that these symptoms are often viewed as "normal" emotional states (code 2.1) or associated with being labelled as "crazy" (code 2.2). Furthermore, participants highlighted the prevailing discrimination and stigma faced by individuals with mental health issues in society (code 2.3). Many participants (n=7) believed that talking to trusted individuals (code 3.1) and obtaining specialist care (n=9; code 3.2) were effective ways to alleviate the burden of mental illness. Additionally, gaining education and knowledge about mental health (code 3.3) was seen as a valuable approach. Only participants from Badulla mentioned engaging in religious rituals (code 3.4) as a further option. Others referred to managing these problems independently (code 3.5). Regarding specialist mental healthcare, concerns included unequal access (code 4.1), fear of overmedicalization (code 4.2), and worries about stigma (code 4.3).

**Table A2. Mental health beliefs of community members**

| **Code** | **Definition** | **Example quotes** | **Coding rule** |
| --- | --- | --- | --- |
| **1. Understanding of mental problems** | | | |
| 1.1 Perceived as mental burden | Participant referred symptoms described in the case vignettes to an increased mental burden.  Participants mentioned specific mental illness conditions. | Increased mental burden:  *“So she does think that both these characters that Venuri and Avi have something that is bothering them mentally.” (Nanny & Household help, woman, Badulla)*  *“Mr. [name] opinion is that Venuri seems to be having a mental health problem because of the fact that she's always sad” (Police officer, man, Badulla)*  Specific mental illness conditions:  *“So I think that from what you said, I'm pretty sure that Venuri may have depression and Avi may be having schizophrenic thoughts.” (IT worker, woman, Colombo)*  *„[…] these are signs of depression.” (Teacher, man, Badulla)* | *Participants identify the problems from the case vignettes as any form of mental/psychological/cognitive burden or problem.* |
| 1.2 Work-related reasons | Participants related the described symptoms to the increased pressure or stress related to work (job, school or university). | *“So what he's saying is maybe there is a reason for a mental health condition that is bothering her, but it may be linked to her profession, to her occupation. So he's focused on the occupation but he's saying that maybe we should look at whether she has a mental health issue that is related to occupational stress or whatever that would come with it.” (Farmer, man, Badulla)* | *Participant associate/explain the problems seen in the case vignettes with any type of work.* |
| 1.3 Family-related reasons | Participants associated the described symptoms with a disrupted family background. | *“And she may be coming from a broken family. Her backgrounds may not be good. Very good in a sense not happy and contented.” (School principle, man, Badulla)*  *“a lot of Sri Lankans get the mental problems through family. I think the biggest thing would be family.” (IT worker, woman, Colombo)* | *Participant associate/explain the problems seen in the case vignettes with the family.* |
| 1.4 Other reasons | Participants associated the described symptoms with other reasons.   - Media-related reasons | *“He must be in this kind of situation basically because of the literature. Basically because of the horror films he may have read.” (School principle, man, Badulla)*  *“[…] in the case of Avi she thinks that it's possible, especially in Sri Lankan context, that this is a child who watches a lot of cartoons and because of the fact that they're watching so many cartoons, they have gotten this idea of like I'm like a King kind of complex in their mind.” (Teacher, woman, Badulla)* | *Participants associate/explain the problems seen in the case vignettes with any other reason.* |
| **2. Associations with mentally ill** | | | |
| - 1. Normalisation of symptoms and dismissal | Participants described that mental illness symptoms can easily be overcome and are perceived as normal emotional states. | *“So I feel like everybody goes through like these kind of emotions at some point in their life, and you just got to remember that everything passes. You know, you are just going to be feeling that in this moment only. And like, yeah, I'm going to be feeling like this for the rest of your life. […] You just got to be positive and keep going and look for more opportunities to do the stuff that you like doing.” (Freelance writer, man, Colombo)*  *“Venuri's problem is not bad, I think, compared to the Avi. […] But I feel that both of them, they can get rid of this mental situation nicely.” (Banker, man, Badulla)*  *“All beings, I mean, human beings in the world are mad to a certain extent. Let's say, I am a , great cricket fan, I don't follow cricket now. It was my madness because my [Sinhala term] stayed. I mean, I've been a voracious reader. I read and sometime till 2-3 o'clock I read, in the morning. So that too is kind of a madness I feel, once you clarify and get the explanation.” (Teacher, man, Badulla)* | *Participants dismiss the problems presented in the case vignettes or perceive them as “normal”* |
| - 1. Crazy | Participants explained that a mentally ill person is labeled as crazy by society. | *“Because in a village, if a person goes to doctor to tell about their mental conditions, as I said earlier, the other villagers might think he's a crazy person. That is the word they're using for a mental unfit condition.” (Banker, man, Badulla)*  *„Now, in Sri Lanka, the people are reluctant to go to psychiatrist because there is thing like, you know, they think they are crazy. They don't want other people to know about it, and they want to hide these things. This thing, I think that is also hindrance. You know, why they don't go to a psychiatrist in Sri Lanka.”(Banker, man, Colombo)* | *Any statement mental health problems with being crazy.* |
| - 1. Discrimination and stigma | Participants explained that mentally ill people are isolated from the society | *“[…] first of all, at the beginning I told you here in Sri Lanka, when you have a mental disorder, you are a rundown in the society.” (Banker, man, Colombo)*  *“Because in Sri Lanka, there's a lot of stigmatization around mental health. And people don't want to come forward and speak because they feel like they will get outcast from society.” (Police officer, man, Badulla)*  *“And they get ignored from the society.” (Teacher, man, Badulla)* | *Any statement in discrimination and stigma is associated with people having mental problems.* |
| **3. Ways to deal with mental illness** | | | |
| - 1. Talking | Participants described that talking to trusted people, such as close family members or friends, is one way to deal with mental illness symptoms. | *“she also thinks that if they have someone like a companion or a sibling that they can talk to, then this problem can be rectified to a great extent.” (Nanny & household help, woman, Badulla)*  *“The primary way to get through to them is perhaps is to have someone who they can trust, speak to them so that they can speak about their concerns.” (Teacher, woman, Badulla)* | *Any statement in which the way to deal with mental problems is associated with talking to any other person.* |
| 3.2 Obtaining specialist care | Participants described obtaining help from specialists is one way to deal with mental illness symptoms. | *“[…] but also if she feels that she's overthinking about it or that it's affecting her life so much, she should seek the assistance of a mental health professional.” (Police officer, man, Badulla)*  ***“****So she says that in both cases Venuri and Avi. There are therapy and counseling centers which are located around the island in Sri Lanka. And a lot of the hospitals in base hospitals also provide psychological and psychiatric services. And further, there are little societies and community level services available in some parts of the country that people like this can go to, for like basic treatment levels.“ (Midwife, woman, Badulla)* | *Any statement in which the way to deal with mental problems is associated with obtaining any type of specialist care.* |
| 3.3 Education and knowledge | Participants described that gaining knowledge about mental illness symptoms is one way to deal with mental illness symptoms. | *“I think it should be normalized, right? These kinds of mental conditions are very popular in the community. But the problem is they don't know what the problem they have actually. That is the only problem I can see. So by educating them and by it's being normalizing, it'll be a great opportunity to students and the people to learn what is the condition they have, and they are presently feeling or facing the problems.” (Banker, man, Badulla)*  *“Or she has to read more about these situations.” (School principle, man, Badulla)* | *Any statement in which the way to deal with mental problems is associated with education and knowledge.* |
| 3.4 Religious practices | Participants described that performing religious rituals is one way to deal with mental illness. | *“[…] parents would go to temples they would go to religious leaders and clergy. And they would try to take the help of various religious rituals like tying chanted threads and things like that, and prayers to like kind of get their child cured or to get them some kind of success in life.” (Nanny & Household help, woman, Badulla)*  *“[…] in her experience, she's seen that a lot of people whether it's Buddhist, whether it's Tamil, they always get directed into this area of Kovil and blind believes like religious ceremonies and trying to exercise demons out of people's bodies, and the end result of all of this she has seen is most often suicide because the person has not received the actual help that they need.” (Midwife, woman, Badulla)*  *“The mental things. And in Sri Lanka, many children are taught to worship. And they lie down in front of the parents and adults and elders.” (School principle, man, Badulla)* | *Any statement in which the way to deal with mental problems is associated with religion (i.e., religious practices, connecting to religious leaders etc.)* |
| - 1. Dealing alone | Participant described that mental illness needs to be dealt with alone. | *“And I think she can get rid of this situation by herself […]” (Banker, man, Badulla)*  *“People find their mental -- I mean, people find answers for their mental health by themselves.” (School principle, man, Badulla)* | *Statements in which mental problems need to be dealt with on ones own.* |
| **4. Associations with specialist mental healthcare** | | | |
| 4.1 Unequal access | Participants described that access to mental healthcare is not equal for everyone. | *“I mean, it's hard for an ordinary person to have access to a person like that in a country like Sri Lanka.” (Priest and school principle, man, Colombo)*  *“But then again it's like these people are interning at psychological institutes and stuff like that. And that's why they've even been given that opportunity to go help these children and stuff, you know.” (Freelance writer, man, Colombo)* | *Any statement about unequal access to any mental healthcare services.* |
| 4.2 Fear of (over-) medicalization | Participants described that specialist mental healthcare means (over-)medicalization or institutionalization. | *“Because here, a lot of the times I've heard about people going for therapy and just the doctor prescribing medicine, that's it kind of. And they have even like gotten addicted to the medicine, not really wanted to help themselves kind of. They've just got not exactly addictive, but dependent kind of.” (IT worker, woman, Colombo)*  *“And he too needs counseling, but you know there are types of doctors called psychiatrist. They usually prescribe you medicines. Without medicines, sometimes these kinds of things could be cured, I believe.” (School principle, man, Badulla)*  *“So in Sri Lanka right now with the infrastructure is pretty bad. It's mostly like you channel someone at a hospital or something.” (Freelance writer, man, Colombo)* | *Any statement about inappropriate treatment options for people in Sri Lanka.* |
| 4.3 Fear of stigma | Participants described that people seeking specialist mental healthcare will be stigmatized in society. | *“So what he says is the fear and the stigma of people in Sri Lanka is directly approach a mental health specialist.” (Farmer, man, Badulla)* | *Any statement that specialist MHC is associated with stigma.* |
