# Supplementary material for: Identifying contextual barriers and facilitators in implementing non-specialist interventions for mental health in Sri Lanka: A qualitative study with mental health workers and community members
Source: Glob Ment Health (Camb). 2024 Oct 8;11:e76. doi: 10.1017/gmh.2024.75 (PMC11504943; doi:10.1017/gmh.2024.75)
Supplement: Wijekoon Mudiyanselage et al. supplementary material [file S205442512400075Xsup001.zip › Additional file 8 Knowledge about existing non-specialsit interventions in Sri Lanka.docx]

# **Additional file 8: Coding framework for knowledge about existing non-specialist approaches**

**Understanding the context: What was the knowledge and opinions about existing non-specialist interventions in Sri Lanka?**

Summary: Some participants were unaware (code 1.1), while others were aware (code 1.2) of existing non-specialist approaches in Sri Lanka. Those who were aware mentioned non-specialist involvement in outreach interventions (code 1.2.1), especially in form of prevention or promotion programs in schools. Others, reported on care delivery by trained non-specialists (code 1.2.2) or by non-specialist without sufficient training and supervision (code 1.2.3). Particularly mental health workers unveiled their concerns about the lack of regulations and monitoring in these existing non-specialist interventions (code 2.1.1), while concurrently expressing the benefit of increasing access to mental health interventions (code 2.2.1) through such interventions.

**Table A3. Knowledge and perceptions on existing non-specialist interventions in Sri Lanka.**

| **Code** | **Definition** | **Example Quotes** | **Coding rule** |
| --- | --- | --- | --- |
| **1.Knowledge about existing non-specialist models** | | | |
| 1.1 Unawareness of existing non-specialist interventions | Participants were unaware of existing non-specialist interventions in SL. | *“But no, I haven't heard of specialist training non-specialist and a non-specialist doing the work. No.” (IT worker, woman, Colombo)*  *“I would almost say, non-existent. There might be things that I'm not aware of.”* *(Mental health worker, woman, Colombo)* | *Any statement indicating unawareness of any non-specialist strategy.* |
| 1.2 Awareness of existing non-specialist models | Participants describes how existing non-specialist interventions look like:  i.e., type of intervention (i.e., outreach vs. task-sharing care delivery), training and supervision of non-specialists. | *See examples for specific sub-groups below (example quotes for codes 1.2.1 – 1.2.3)* | *Any statements indicating awareness of any non-specialist strategy.* |
| 1.2.1 Outreach approaches | Participants described that non-specialists work in government or non-government-based outreach models, mostly in form of teacher trainings to increase mental health awareness or workshops at the community level in deprived areas. | *“I've only been exposed to one non-specialist care model in Sri Lanka, which is this model that was conducted through CAFS actually, in collaboration with WHO also known as The MH Gap Study. […]and that it's going to specific low- and middle-income areas in Sri Lanka, specifically in the north, where they have faced a lot of traumas with a very small amount of training, and a very minimal amount of mental health awareness. And at the same time, a lot of stigmas. So they have gone to these specific areas, and essentially conducted awareness sessions, which are a part of the MH Gap where they would essentially talk about mental health, talk about stigma, bridge that gap between misinformation and what is already existing. To educate the general public on how to identify specific issues, and what places you can get in touch with once you do identify these issues” (Mental health worker, woman, Colombo)*  *“In Sri Lanka, recently they recruited near 100 or 200 teachers as counselors/teacher councellors. Now they are in the government schools. And nowadays I'm also engaging -- I develop several for them. They give specific training for these teachers. Counseling teachers under the Ministry of Education, and especially in western province. Western province, they do have a separate training program. Southern province, they do have another program. And somehow, they are providing this training for teachers to identify the psychological problems. And help the children with the symptoms and if needed, do the referrals.” (Mental health worker, woman, Colombo)* | *Any statement about knowledge on any non-specialist outreach intervention.* |
| 1.2.2 Non-specialist care providers with training | Participants reported on primary-deliverer approaches, where lay people receive training to provide care. | *“Actually, at the Colombo Theological Seminary there is a theological training center where they train lay people, not clergy. Lay people to do counseling. That's kind of a certificate course that runs for six months. You know, they work in Christian communities, but they're not really qualified counselors.” (Priest & school principle, man, Colombo)*  *“Nowadays there are courses like caregivers and nursing assistants. It's a short-term course, they receive training for three months or six months and they start to work.” (Mental health worker, woman, Colombo)*  *“There are people that sometimes are called social workers, mental health social workers. I mean, various kinds of names are there but they have to get some training before going for the treatment, for the patient.” (Mental health worker, man, Badulla)* | *Any statement on any type of care intervention involving non-specialists who received training.* |
| 1.2.3 Non-specialists without sufficient training and no supervision. | Participants reported on non-specialist delivered care without sufficient training background or supervision. | *“So recently, one so-called counselor went for the program, like awareness program in public sector to give an awareness to police officers. And she said homosexuality is a disease disorder, which should be treated. And she says she can convert those people to be like other people. So this was a huge issue recently. Apparently, she hasn't obtained proper education on this.” (Mental health worker, woman, Colombo)*  *“There are unethical practitioners, even if you find them in the north or the south. So there are stories that I have heard on like personal accounts as to how counselors work with their clients. I mean, sometimes it could be because of insufficient. Like the insufficient education.” (Mental health worker, woman, Colombo)*  *“[The midwifes] do not receive any form of hard and fast mental health training. Whatever training that they have is essentially for their midwife duties and responsibilities and it just from what I gather it's just touching a very lightly on the mental health component and the communications component. But they have not been issued any specific mental health training nor have they been given any certifications or anything of the sort. […] So she does not have a supervisor who overlooks mental health, whatever services that is provided by her. What happens is when someone shares that they have certain kinds of mental health problems, she would give them counseling based on the basic and minimal experience and exposure that she has had. She would carry on these counseling sessions for two or three times and then she will follow up with these people and see if it has been helpful to them. Have they been able to improve their condition? Do they observe some kind of positive change or progress? If that has not happened the next step is that she directly turns them towards the hospital, the base hospital or the general hospital that is relevant and puts them into counseling in the hospital.” (Midwife, woman, Badulla)* | *Any statement on any type of intervention involving non-specialists who did not receive adequate training and/or supervision.* |
| **2. Benefits and concerns about existing non-specialist approaches** | | | |
| **2.1 Concerns** | | | |
| 2.1.1 No regulations and monitoring | Participants reported the absence of regulations regarding existing non-specialist approaches and governing bodies to control such interventions. | *“So nobody knows how much [the non-specialist] charges and her practice is not monitored by anyone else, and we don't know what she does. And she sometimes bring her clients to home and do some very informal kind of session.” (Mental health worker, woman, Colombo)*  *“Otherwise, another thing is these non-specialists are coming from the various courses in Sri Lanka. So I think it is a little bit hard to have proper monitoring and quality assurance mechanism for them.” (Mental health worker, man, Badulla)* | *Any statement about concerns regarding regulations and monitoring in any non-specialist intervention.* |
| **2.2 Benefits** | | | |
| 2.2.1 More accessible mental health interventions | Participants explained that the existing non-specialist interventions makes mental healthcare more accessible and thus fills the existing MHC gap. | *“And these people, through this whole non-specialist approach, they can actually support. They can intervene and fill in the gaps” (Mental health worker, woman, Colombo)*  *“So as I said pros, like we have a lot of people to reach out now because of this thing.” (Mental health worker, woman, Colombo)* | *Any statement that existing non-specialist interventions help to make mental health interventions more accessible.* |
