# Supplementary material for: Identifying contextual barriers and facilitators in implementing non-specialist interventions for mental health in Sri Lanka: A qualitative study with mental health workers and community members
Source: Glob Ment Health (Camb). 2024 Oct 8;11:e76. doi: 10.1017/gmh.2024.75 (PMC11504943; doi:10.1017/gmh.2024.75)
Supplement: Wijekoon Mudiyanselage et al. supplementary material [file S205442512400075Xsup001.zip › Additional file 9 Barriers and facilitators to implementation of non-specialist approaches.docx]

# Additional file 9: Anticipated implementation determinants for non-specialist mental health interventions in Sri Lanka

| **Theme 1: Concerns about effectiveness, acceptance and feasibility**  **Description: Participants identified various concerns regarding the effectiveness, acceptance and feasibility of implementing non-specialist mental health interventions. This was observed across the general non-specialist model and specific intervention types, including outreach, stepped-care, primary delivery, and non-specialist interventions involving digital technologies.** | | | | |
| --- | --- | --- | --- | --- |
| **Code** | **Description** | | **Quotes** | **Coding rule** |
| ***Type 1: General non-specialist mental health interventions***  ***Definition: The general concept of involving non-specialists in any mental health interventions.*** | | | | |
| 1.1.1 Concern about ineffectiveness | Participants expressed doubts about the effectiveness of utilizing non-specialists in mental health interventions.   - Because of the danger of inadequate training - Because of potential adverse effects especially with regards to non-specialist-delivered treatment in contrast to specialist-delivered treatment | | *“You can't essentially blame them [non-specialist] either because they haven't gotten the intensive training that someone like I have gotten, I had to study for four, five, six years to get to the place that I'm at right now. At least two of those years were in training, where I have been corrected on the way I'm asking a question, I have been corrected on my line of thought, I have been corrected on taking myself out of the equation in a conversation, which is very difficult to do for people in regular, it's not something that everyone should be able to do” (Mental health worker, woman, Colombo)*  *„Honestly, this is a personal belief that I don't think that we can tell people in Sri Lanka, “If you see something, this is how you help.” I don't think people should be taught to help especially in a mental health-related setting.” (Mental health worker, woman, Colombo)*  *„ I don't think that's a solution because they can really -- I mean, this is my personal experience. If they have little knowledge, and that they tend to create more issues […]Because they're not properly trained, as I said. I mean, there will be a few who will be able to take this, you know, small training and to do it properly. But I have seen more who will not be capable of doing it right.”* *(Religious leader, man, Colombo)*  *“Because any person who has a situation, I mean, the mental situation will want some help, but they will be reluctant to go to somebody and ask, especially when you know that person is not a professional in that area.” (Religious leader & school principle, man, Colombo)*  *“She believes that going to a mental health doctor in itself would give that person more help because the doctor will be more controlled than all of these [non-specialist care] strategies. They’ll be able to directly administer medications or if required, various intravenous medications that will help the person immediately. And they would be in a position that would allow them to have more control over the patient’s conditions than training other people. [Despite constant supervision by a specialist] […] She still believes that it's more effective and safe also to go to a specialists because she says even though people have been given training and they're being supervised, there is a chance that they may have missed certain sections of the training or they may not have understood or maybe they're doing it wrong or they’ll make a mistake, or maybe they're just not as competent as the doctors is. So she anyway believes it's safer and more effective for people to go directly to a mental health specialist.” (Nanny & household help, woman, Badulla)*  *“Yeah. But I still feel like -- because the specialist is kind of more trained and stuff. And in that kind of visits, you have to kind of go visit them. But I feel like if I have to go visit my social worker as well, it's like I could just go to my specialist kind of.” (IT worker, woman, Colombo)*  *“Yeah. Barriers are basically the people who will look at qualifications and be like, yeah, who is this person? She's not even being trained properly.” (Freelance writer, man, Colombo)* | Any statement in which participants expressed doubts in the effectiveness of any mental health interventions in which non-specialist are involved. |
| 1.1.2 Concern about increased stigma and discrimination | Participant expresses the concern that implementing such interventions would create more stigma and discrimination for the those involved in the implementation process (i.e., also the non-specialist) | | *“Yes. So even the individual coming who sort of try implementing this would be called a mad guy.” (School principle, man, Badulla)*  ***“****Somebody, a social worker who try to promote this kind of very positive attitude to the society would also be called a mad guy.” (School principle, man, Badulla)* | Any statements in which participant expressed that any mental health intervention involving non-specialist would create/increase any form of stigma or discrimination. |
| 1.1.3 Concern about privacy | Participants were concerned about the privacy of potential service users, because…   - non-specialists would not warrant adequate privacy protection and divulge personal information | | *„not always beneficial for the students or persons who need the service. The problem is the privacy. They are not going to keep the privacy of the person. It is like gossip. They are always not going to keep that a secret, it should be a secret because it is an illness. They sometimes go and tell the other person, “That person has this and that person has this.” (Mental health worker, man, Badulla)*  *“But sometimes some teachers are doing the correct thing, the only thing is privacy. Yes.” (Mental health worker, man, Badulla)*  *“Yes, that is going to be a problem because, for a non-medical person, they are not going to give us the exact picture. It's again going to be a privacy-related problem.” (Mental health worker, man, Badulla)*  *“I mean, if you are not professional, you will go and speak to another person. Now, there will be a problem for the person. So that professionalism has to be there, I think“ (Religious leader & school principle, man, Colombo)*  *“I have seen teachers, not that I'm blaming/saying anything bad about any teacher. But I have seen teachers how much ever we tell them not to speak about their children in their classes, in the staff room, and on corridors not to do that. They go on doing that. And sometimes they come and tell us, especially to the school management, that they are trying to help the children. And then on the other hand, we hear that they have gone and spoken to someone else about the same child. So, I don't know whether we will be able to stop that, because that is in that person's character. So it'll be difficult to change that. I'm talking about Asia here. […] (Religious leader & school principle, man, Colombo)*  *“I think that might be one, because that -- if it was me in this situation, then I kind of would go to specialists for wanting that privacy.” (IT worker, woman, Colombo)* | Any statement in which participants expressed their concerns about privacy issues in any mental health interventions involving non-specialists. |
| 1.1.4 Concern about the current economic situation | Participants expressed their concerns about the current economic crisis, which may cause:   - “brain drain” leading to a reduction of available specialist mental health workers in the country who could supervise or train non-specialists - No interest and engagement of community members in mental health interventions due to other difficulties that they need to face | | *“Those opportunities are very less, we don't take people and train them. But that has to be there because we have-- not many specialties are there. Even the ones that are practicing are looking for opportunities abroad, and now they're all migrating because of the crisis here in Sri Lanka. So that is a big blowout for us, even the medical sector. Already, I think half of the surgeon’s community has migrated.” (Mental health worker, woman, Colombo)*  *“Actually, the limitation of Sri Lanka is currently, again, the human resource problem. So as we are aware that there are some economical crises going on. And so a lot of train professionals are having good jobs in other countries. So they are migrating in a very rapid rate. Currently the main problem is that we are losing the best specialized consultants and doctors and other staff trained in psychiatry.” (Mental health worker, man, Badulla)*  *“Then given the current economic situation in the country and all of that if there is no method to how we are implementing it then of course it will not be successful.” (Teacher, woman, Badulla)*  *“But what she's seeing given the current situation in the country, she's not pretty sure that people will be interested in following like taking and getting involved in these kinds of things.” (Teacher, woman, Badulla)*  *“Many are leaving the country, because if the husband is kept in Jaffna, wife is working in Galle. Leaving the country. So then those are political things which will not help your research and your interview. But you know, let's say the scarcity of doctors, counselors, right? So people don't take up. Many people are getting qualified in the field of psychology. Now it is offered to them as the first-degree subject. But still they don't come back to the society. They do some other job.” (School principle, man, Badulla)* | Any statement about the current economic situation being a barrier to the implementation of any mental health interventions involving non-specialists. |
| **Type 2: The collaboration stepped-care approach**  **Definition:** **This intervention comprises a collaborative care type involving three interventions providers. First the non-specialist provides low-level psychosocial prevention intervention (i.e., breathing exercises) and a non-specialist medical doctor is responsible for providing medication and additional support if required while both are being supervised by a specialist. If these steps do not lead to any progress with regards to the mental health problems, a specialist provides treatment.** | | | | |
| 1.2.1 Concern about being too overwhelming | Participants were concerned that the collaborative stepped-care approach would be too overwhelming for service-receivers. | *“She thinks this because in the previous approach, there are three people involved in the collaboration. So she sees that as a complex communication system” (Midwife, woman, Badulla)*  *“Maybe because when you go to a specialist, you feel like it's a one-on-one thing. And you share those personal details with that specific person. So maybe they might be reluctant for it to be a whole team.”(IT worker, woman, Colombo)*  *“But I don't know, maybe it might be a bit overwhelming. It would be easier if like one person was overlooking the entire thing, maybe. Is what I feel. […] just having to go see so many different people.” (Freelance writer, man, Colombo)* | | Any statement relating to the complexity or overload associated with the collaborative stepped-care approach. |
| 1.2.2 Concern about the lack of human resources | Participants were concerned about the lack of resources to develop a collaborative care team, especially because…   - Lack of resources in the medical sectors (i.e., difficult to involve a general medical doctor as a non-specialist) - Lack of volunteers who could work as non-specialists | *„What you're talking about is more generic, like a GP level. Because I come from the UK, that would make sense because we have the GP there, but here, we don't have GPs.[…] A lot of people, even people with less money go to a private GP. […] So it's very, very difficult from a top-down approach to identify people through the medical system. (Mental health worker, woman, Colombo)*  *“I think that that intention [of the stepped-care intervention] is very good but the problem is we do not find a such number of volunteers and it will not be sustainable […]”(Mental health worker, man, Badulla)*  *“Our clinics are very heavy, we small number of doctors. The psychiatric, yes, of course, need a lot of time to spend with one patient. The clinics are very heavy and only one consultant.” (Mental health worker, man, Badulla)*  *“As I already said, there's a shortage of staff.” (Mental health worker, man, Badulla)*  *And given the political and economic situation in Sri Lanka, we have a lot of doctors leaving. We have like a very small pool of mental health professionals who are back in the country and struggling with it. (Mental health worker, woman, colombo)*  *“I'm talking about rural setting, where those hospitals are understaffed. And there are so many patients who are going to those hospitals. And I don't think that in Sri Lanka and the context, they have enough time to handle them.” (Religious leader & school principle, man, Colombo)*  *“[…] even in the rural areas, there are good hospitals. But they are understaffed. […] There's only one doctor for the entire hospital. Those are small rural hospital run by the government. And so this one doctor actually cannot really take care of people who need counseling and that kind of mental support in addition to his other work. So I don't think here in Sri Lanka it'll work, looking at the current situation.” (Religious leader & school principle, man, Colombo)*  *“I'm talking about rural setting, where those hospitals are understaffed. And there are so many patients who are going to those hospitals. And I don't think that in Sri Lanka and the context, they have enough time to handle them.” (Religious leader & school principle, man, Colombo)* | | Any statement relating to the concerns about the lack of human resources or difficulty to engage the required human workforces for the collaborative stepped-care intervention. |
| ***Type 3: The sole-provider care approach***  ***Definition:*** ***This intervention comprises one non-specialist who provides the low-intensity treatment alone, particularly in resource-poor areas where no specialists are available.*** | | | | |
| 1.3.1 Concern about incompetency to deliver treatment alone | Participants did not believe that a non-specialist would be competent enough to provide treatment alone, in contrast to providing treatment in a collaborative care team. | *“In this case, the only person is getting the knowledge, getting experience to do the intervention. This is just going to work for a small group of people.” (Mental health worker, woman, Colombo)*  *“But I don't think that they will be professional enough to go and talk to people with mental problems; and if there are problem, to fully counsel them alone.” (Religious leader & school principle, man, Colombo)*  *“[the sole-provider approach] is not a very successful thing. I think that they don't have much knowledge. […] If it is a group or if it is a community, that will be all right. But a person, I don't think that they can handle the situation like this.” (Banker, man, Badulla)* | | Any statement relating to the concern about the effectiveness of interventions in which non-specialist provides treatment alone (sole-provider approach) in contrast to the non-specialist being involved in a collaborative care team. |
| ***Type 4: The school-based educational outreach approach***  ***Definition: This intervention is a school-based outreach intervention type in which teachers receive basic mental health education to teach students on mental health and raise general mental health awareness and to be able to identify, refer and deal with children with mental health problems.*** | | | | |
| 1.4.1 Concern about increasing discrimination in school settings | Participants were concerned that providing a school-based outreach interventions in which teachers could identify students with mental health problems would lead to discrimination of those students. | *„So even though a teacher identifies, “Okay, this child is struggling with concentration, is struggling with emotional regulation, I have to refer them to a psychologist.” That may not change the way that they treat the child, but they might still be discriminatory towards a child within a classroom setting, even though they have known that there is something wrong because there is an internal bias that is impacting that.” (Mental health worker, woman, Colombo)*  *“[…] also maybe also stigma because when other people hear this student has, I don't know, depression they might feel bad.” (Mental health worker, man, Badulla)*  *“And the stigma problem is still there in certain areas. And some teachers themselves they have stigma. They might ask the teacher what is the problem with that child? Why the child came to you? So maybe that child is neighbor, they're very curious to know what is happening inside the counseling room.” (Mental health worker, woman, Colombo)*  *“Of course, it could help them but if it gets highlighted then these students like Venuri and Avi who have mental health issues will now be stigmatized by the rest of the students who now start to think, “Oh, these people are crazy. That is why they can relate to what the teacher is saying.” And that they will kind of get outcasted from the general student population in the school.” (Nanny & household help, woman, Badulla)* | | Any statement about the potential increase of discrimination in school settings as a result of implementing the school-based educational outreach approach. |
| 1.4.2 Concerns about potential disagreement from parents | Participants were concerned that parents of school children would not accept the implementation of such interventions, due to:   - Stigma around the mental health topic - Competitive academic structures | *“You also hear children who come second in the class and don't come first and they go home and their parents beat them. There is a real hunger or a real obsession with this idea of success. So tying whatever you are going to do, to that idea of success, will get the parents to immediately engage. Because if they believe, ‘Oh, this will make my child earn more money in the future? I’m there, 100%.’ But if you say, ‘Oh, this will make your child happy. Who gives a shit. Let’s go home, you have to do your homework.’” (Mental health worker, woman, Colombo)*  *“Because I feel like the parents themselves are like normally, "You can't teach my kids this and that." (Mental health worker, woman, Colombo)*  *„first of all, at the beginning I told you here in Sri Lanka, when you have a mental disorder, you are a rundown in the society. You know, that's why people are reluctant to go to psychiatrists. So then then these teachers, when they meet up with parents and if they discuss these things, you know, these individual children's behavior and things like that, then some parents may not like it if there have children some sort of a disorder.“ (Banker, man, Colombo)*  *“But when you take the parents in a large picture, then there could be issues. That's what I fear in Sri Lanka. Because most of them although they have disorders, although they take medicine, they don't, say that they're taking medicine. They try to hide these things in Sri Lanka. They're not open.” (Banker, man, Colombo)*  *“But when it comes to our parents, they are reluctant to try this. Some parents are -- you get the lucky kind where they'll be like, okay, you need to talk to someone. But because of the culture, a lot of the old people, they are very reluctant to seek help because of, I guess, reputation and people talking. That's a huge problem here actually.“ (IT worker, woman, Colombo)*  ***“****I mean, of course the only problems I can see are some parents. But, you know, that's the way some parents are just like that. I guess they're just looking for anything to complain about.” (Freelance writer, man, Colombo)* | | Any statement about potential disagreement from parents when implementing the educational school-based outreach intervention. |
| 1.4.3 Concern about the strict education system | Participants were concerned about the strict education system which may not support the implementation of such mental health programs in schools. | *„And some principals, they don't understand why this is important and why this teacher should not be given other tasks” (Mental health worker, woman, Colombo)*  *“So I don't think if you are trying to do something immediately with them, there will be lot of persistence from the system itself; from school itself. They won't support you to do that.” (Mental health worker, woman, Colombo)*  *“In Sri Lanka education system, they have organized this education system for few subjects. In Sri Lanka, they don't have -- what do you call? They don't have much opportunities to select the subjects. And they don't have much time to select subjects, to do as what they want. But that is the difficulty” (Banker, man, Badulla)* | | Any statement about difficulties to implement the educational school-based outreach intervention due to the strict education system. |
| 1.4.4 Concerns about the high workload of teachers | Participants were concerned about the high workload of teachers who act as non-specialists in such interventions. | *“And, how will those teachers manage that workload? Because they already have that classroom, they already have their workload, but if they have to also deal with student referrals, how will that be compensated?” (Mental health worker, woman, Colombo)*  *“Actually the main difficulty will be the engagement of the teachers. Because now the teachers will be busy and they're exhausted. And because our schools are not the ideally optimize one. You may be aware that some schools, some classes are around 50 students in there. In our country, there are popular schools. So these popular schools are having children up to 50.” (Mental health worker, man, Badulla)*  *“In my school, which is a small one, average size is 20. In the city schools, it is over 50 sometimes. So it's very difficult.“ (School principle, man, Badulla)* | | Any concerns about the high workload for teachers when implementing the school-based educational outreach intervention. |
| ***Type 5: Non-specialist mental health interventions involving digital technologies***  ***Definition: Any involvement of any type of digital technology in any non-specialist mental health interventions.*** | | | |  |
| 1.5.1 Concerns about the poor digital infrastructure | Participants were concerned about the poor digital infrastructure in Sri Lanka:   - Lack of resources when service receivers are required to use their own technological devices, especially in rural areas - Lack of resources specifically in schools - Connectivity issues | *“Saying that let’s say the app would require you one phone per participant, that will become problematic because maybe each family only has one phone to share between two parents and whatever number of three, four, or five children maybe. So bringing those kinds of things into awareness, that may be a large number of people who need to use one phone, maybe like multiple accounts, and how would you then data protect each account so that there's no issue?” (Mental health worker, woman, Colombo)*  *“[…] it's too early to go through technology, unfortunately, because if you look at the majority of schools, the public schools, the government schools, where you will receive the biggest reach to a population of students or teachers, they do not have a lot of technology. A lot of government schools in Sri Lanka, do not even use email. They still work through public lead, like the mailing system, where you mail letters, even the Minister of Health doesn't.” (Mental health worker, woman, Colombo)*  *“In terms of technology, I don't think Sri Lanka is ready for it if I'm being honest.” (Mental health worker, woman, Colombo)*  ***“*** *the only thing is with Sri Lanka I'm not too sure of how we can do that in terms of technology only because of the economic condition here and the lack of technology in general.” (Mental health worker, woman, Colombo)*  *„[…] There may be some limitation because we are actually third world country. We are not having a good technological support now because, as I said, we're in economic crises.[…] Only poverty because they can't afford the devices. ” (Mental health worker, man, Colombo)*  *“If you have three kids at home who are going to school, parents find it very difficult to provide devices to them. They have only one device at home. So if all three kids have classes at the same time, there's a huge fight inside the house who to be given the device. I know personally some parents find it's very difficult to like providing them these devices, mobile devices, laptops, right?” (Mental health worker, woman, Colombo)*  *“There are some issues relating to this connectivity. I mean, in Ratnapura we have connection problems and most of the services they don't work properly.” (Mental health worker, woman, Colombo)*  *„But again, with Sri Lanka it will depend on the demographics that you're looking at. So children in Colombo or some of the suburbs may have access to technology, but children in the rural areas may not. But I believe they would still need that equal access to knowledge on mental health.“ (Mental health worker, woman, Colombo)*  *“but what he says is that the economic condition of the country, even though there are people who are interested in dealing with technology and maybe they even have the talent and the skill to carry it out, there is a lack of equipment and lack of resources that would allow technology to play that seamless role that it has to play. So he says that he sees that there is a big deficit in procuring the resources which are required.” (Police officer, man, Badulla)*  *“So he says that certain schools in Sri Lanka may not have the technical facilities, technological facilities to support this kind of program […]”(Farmer, man, Badulla)*  *“So according to him, the challenge would be that certain areas in Sri Lanka would not be able to post these kinds of programs because they lack technological and other resource facilities to do so.” (Farmer, man, Badulla)*  *“So what she's saying is compared to other countries, Sri Lanka is lacking in technology. And even if we look at hospitals, we may not necessarily have all of the technical equipment or the resources in terms of technology that we need. So to implement something like this in Sri Lanka, maybe will not be accessible by people who live in extremely rural areas. And she used what just happened as an example the other lines just dropped off while she was talking to us on the call. So in areas like Badulla, Bandarawela or even more remote areas like Moneragala, like really, really rural areas where people don't have access to a good network connection. It may be difficult for them to access a technical kind of role in this.” (Teacher, woman, Badulla)*  *“Because like if you're talking like -- as long as you got the infrastructure to get it done wherever you go.” (Freelance writer, man, Colombo)* | | Any statement about the poor digital infrastructure that may impose a barrier to involving any form of digital technologies in non-specialist mental health interventions. |
| **Theme 2: Perceived Benefits in reducing the mental healthcare gap**  **Description:** **Participants identified various benefits in non-specialists’ mental health interventions. This was observed across the general non-specialist model and specific intervention types, including outreach, stepped-care, primary delivery, and non-specialist interventions involving digital technologies.** | | | | |
| ***Type 1: General non-specialist mental health interventions***  ***Definition: The general concept of involving non-specialists in any mental health interventions.*** | | | | |
| 2.1.1 Beneficial increasing access to mental health interventions | Participants perceived that involving non-specialists in mental health interventions may help to increase the access to mental health interventions in the country by:   - Increasing the human workforces who could provide treatment for those in need - Making care more accessible and affordable - Generally increasing awareness by sharing specialised knowledge with the community | *“For one because it reduces stigma, and therefore it allows people to start that conversation about mental health. It could be something as simple as within a family. If one parent is educated on the importance of mental health awareness, that will trickle down to their children, and that will trickle down to how they live life on their own, it will impact the way they deal with their friends, their peers, how they deal with issues that they have to go through it. So, when one person in one community or one family unit has been educated, it has a giant impact on how that trickles down to the people in their life. So, we have seen a lot of advantages.” (Mental health worker, woman, Colombo)*  *“But in psychiatry because of the sensitive issue of the patient and also the stigma, and also other background problems like the patients are not getting good income, so they can't afford and they cannot go to tertiary care center. But actually, if they are kept in the society without treatment, it'll still be a problem to the society. So it is a main benefit.”* (Mental health worker, man, Badulla)  *“pros I can say like people can access these counselors easily. So that is because we don't have any counselors or psychological -- I would say psychologist in the government sector. Not at all. In hospital setting, nothing.”* *(Mental health worker, woman, Colombo)*  *“We have only about 20 psychiatrics. And I would say we have 14 licensed clinical psychologists, out of which I don't know how many are in the country. So I only know about three or four who are actively practicing. So you can see the gap there, and the people who are reaching out to support. But if you go to a psychiatric in hospital, the lines that come in for treatment or take medicines is huge. People start lining up at 11:00 AM and dispensation will start at 1:00 PM or 2:00 PM. Sometimes even at 10:00 AM in the morning. They're there because they want to get in the clinic. […] So given that it is supervised and done right, yeah, I think it would be very helpful for countries like India and Sri Lanka. And given the political and economic situation in Sri Lanka, we have a lot of doctors leaving. We have like a very small pool of mental health professionals who are back in the country and struggling with it.” (Mental health worker, woman, Colombo*  *“He believes that that method might be successful because it has that top down approach where information is trickling down from specialists to other people who are connected to the community. So he thinks that it will be possible for people to get more awareness and education about their conditions.” (Farmer, man, Badulla)*  *“But if this kind of method is used, then he believes there is a chance that people will go and get the help that they need because it's not necessarily that they're walking up to a psychiatrist” (Farmer, man, Badulla)*  *“So your suggestion is very good. In Sri Lanka, we don't have much people [specialists]. And we do have to promote these non-specialized communities, and it'll help this kind of people to get rid of these problems.” (Banker, man, Badulla)* | | Statements in which participants see the benefit in increasing the access to any form of mental health interventions. |
| 2.1.2 Valuable because non-specialists are considered more comfortable to talk to in contrast to specialists | Participants perceived non-specialists as more comfortable than specialists:   - Because obtaining help for mental health problems from a non-specialist is associated with less stigma than obtaining help from a specialist - Because non-specialists are perceived as “less scary” than specialists | *“Having somebody from who you can get the right kind of support, but who is not a mental health doctor? Because if you go to a mental health doctor, there is the stigma or the taboo that you're mad, it's completely unacceptable in a way for a lot of people.”* *(Mental health worker, woman, Colombo)*  *“I think so. Because they don't see that person as like professional. Like obviously they're professional. But they don't see them as like the title, right? The psychologist, the psychiatrist. It makes it seem more, I guess, serious. So maybe this way they have someone to talk to as a start.” (Mental health worker, woman, Colombo)*  *“If that's there, then I would feel more comfortable if they're a non-specialist.”* *(IT worker, woman, Colombo)*  *“These cases, they would refer the person [non-specialist] further. (…) Because they don't see that person as like professional. Like obviously they're professional. But they don't see them as like the title, right? The psychologist, the psychiatrist. It makes it seem more, I guess, serious. So maybe this way they have someone to talk to as a start.”(Mental health worker, woman, Colombo)*  *“So what he says is the fear and the stigma of people in Sri Lanka is directly approach a mental health specialist. But if this kind of method is used, then he believes there is a chance that people will go and get the help that they need because it's not necessarily that they're walking up to a psychiatrist.” (Farmer, man, Badulla)*  *“**People might be more comfortable visiting these people instead of actually going to like a hospital or like something.” (Freelance writer, man, Colombo)*  *“And it won't be like overwhelming and scary meeting an actual doctor, or like going to a hospital, or something like that.” (Freelance writer, man, Colombo)*  *“The other thing is, I feel that these mental conditions if they go to a doctor, these people thinks that they have a mental problem. So they do have a shy to go to doctors because they might think that they are full patients. So your suggestion is very good. In Sri Lanka, we don't have much people. And we do have to promote these non-specialized communities, and it'll help this kind of people to get rid of these problems.” (Banker, man, Badulla)*  *“The benefits in the sense, as I said, that if they go to a hospital or if they go to a psychologist, that's a different story. So they think that they are not in good condition. They're not good in mental condition. The social people might think they are crazy people. But the benefit is that, that kind of communities they can go to any time. No one knows about that, then they can go and tell the story.” (Banker, man, Badulla)* | | Statements in which participants express that any non-specialist mental health interventions are perceived as more comfortable in contrast to specialist mental health interventions. |
| ***Type 2: The collaborative stepped-care approach***  ***Definition: This intervention comprises a collaborative care type involving three interventions providers. First the non-specialist provides low-level psychosocial prevention intervention (i.e., breathing exercises) and a non-specialist medical doctor is responsible for providing medication and additional support if required while both are being supervised by a specialist. If these steps do not lead to any progress with regards to the mental health problems, a specialist provides treatment.*** | | | | |
| 2.2.1 Benefit of increasing trust in the effectiveness of treatment | Participants perceived the stepped-care approach as valuable as it would ensure the effectiveness and safety of the care:   - due to the potential case that the specialist can directly care for the service user - due to the collaborative nature of care provision - specifically due to the involvement of a general practitioner as a non-specialised mental health worker | *“Stepped Care.[…] Because they are dealing with so many people. They are dealing with doctors, psychiatrists, and everyone. So they can get experience, knowledge, aspects of the thinking and everything. They can implement that. […] When it comes to Stepped Care, it's going to work for a big crowd, many populations.” (Mental health worker, woman, Colombo)*  *“So I think to avoid mishaps happening, which happens a lot; or misdiagnose happening again, which happens quite a lot, I would say it's best if the lower level care is given supporter while being supervised and then needs to be transferred to someone who is more experienced.” (Mental health worker, woman, Colombo)*  *“And I agree that there should be a psychiatrist, a medical doctor. Maybe they could add a psychologist, but because psychologists look more into the therapy aspect of it, like talk therapy, and intervention space of that. And the psychiatrist may lean towards medication. Right.” (Mental health worker, woman, Colombo)*  *“So she thinks that this method is good and she thinks that because a normal physician has also been trained and a normal social worker has also been trained maybe people can gain more knowledge out of it. It is what she thinks.“ (Nanny & household help, woman, Badulla)*  *“So she thinks that this method is okay compared to the others [primary delivery approach] also because it also guarantees that this person has access to a specialist data, specialist needs to step in. So there they have that kind of trust factor that comes in that you know, it's not just a non-specialist but a specialist is also there. They have access to that person's services and intelligence and information all that stuff. So from her point of view, this strategy is a good one”* *(Teacher, woman, Badulla)*  *“The second thing is, the non-specialized person with a mental health doctor, that will be a great opportunity because the doctor will understand the health condition. And other person, she can talk to the person and get the details. And that combination will be great.” (Banker, man, Badulla)*  *“That is also very good because before it get worse, the mental condition, they can approach this non-community person and non-specialized person and the doctor, the normal doctor. But that non-specialized person and the doctor, they already got the specialized training from another specialist. So they might be aware of these problems. That will be a great thing.**“ (Banker, man, Badulla)*  *“As to my knowledge, the previous one is very good. Non-specialized person with a doctor. That is great. This is also good. […]But I think the most important thing is she should have like the previous scenario, it's a good doctor and non-specialist person.“ (Banker, man, Badulla)*  *„The second thing is, the non-specialized person with a doctor, that will be a great opportunity because the doctor will understand the health condition. And other person, she can talk to the person and get the details. And that combination will be great.“ (Banker, man, Badulla)* | | Statement in which participants express that using non-specialists in collaborative stepped-care interventions would increase the general trust towards this form of care. |
| ***Type 3: The sole-provider care approach***  ***Definition:***  ***This intervention comprises one non-specialist who provides the low-intensity treatment alone, particularly in resource-poor areas where no specialists are available.*** | | | | |
| 2.3.1 Beneficial for establishing a close bond with the care provider | The primary-care deliverer type was perceived as valuable as it would allow to create a better bond between the non-specialist and the service receiver due to a simplified and direct communication pathway. | *“whereas this particular method, she sees it as a more direct and simpler communication method. So what she says is, there are just this is one person who's the point of contact for the person who needs the services. So the grassroots person is directly getting all of their supervision and advisory from the specialist. And then they're giving it to their point of contact, who is the client. So the client is constantly maintaining communication with them only, which allows them to have clearer, more simple communication channels. And it will empower the grassroots person to actually provide their clients with more holistic and a more consistent set of answers and solutions to their problem, as opposed to this client going to have to go to like three different levels to get like their treatment in three different parts.” (Midwife, woman, Badulla)*  *“Maybe because when you go to a specialist, you feel like it's a one-on-one thing. And you share those personal details with that specific person.” (IT worker, woman, Colombo)*  *“[the sole-provider type is preferred]* *Because the number of people are removed from the equation and it's just that one direct line you know […].” (Teacher, woman, Badulla)*  *“I think this is the best, honestly. Because like I said, the patient can develop a strong relationship and bond and feel comfortable opening up with this person.” (Freelance writer, man, Colombo)* | | Statements in which participants express that using non-specialist as the sole provider of care (in contrast to a collaborative care approach) would increase the bond with the service user. |
| ***Type 4: The school-based educational outreach approach***  ***Definition: This intervention is a school-based outreach intervention type in which teachers receive basic mental health education to teach students on mental health and raise general mental health awareness and to be able to identify, refer and deal with children with mental health problems.*** | | | | |
| 2.4.1 Beneficial for increasing the mental health literacy | Participants believed that this intervention would help to increase the overall mental health literacy by:   - Educating children on mental health and thus increasing overall mental health awareness - Educating teachers and sensitising them on how to deal with children having mental health problems | *“I'm always thinking that we have to give a workshop to teachers, we have to give training to teachers because all the students come from different environments because their behaviors, their activities are different from everyone. So, the teacher must know the steps, the teacher must know the background scenes and everything, then only they can handle the children. Otherwise, if they don't know the correct way to treat children, then it's not going to work for their future also” (Mental health worker, woman, Colombo)*  *“Especially when teachers have been trained to identify these certain signs and symptoms, we see that there is a change in the way that they deal with them.” (Mental health worker, woman, Colombo)*  ***„****I think general it is great, because it is vital that children are educated on mental health at a very young age.” (Mental health worker, woman, Colombo)*  ***“****I mean, I think everyone should have some kind of knowledge on mental health. It's like how people have knowledge on basics on, I would say, like physical illnesses. I would say similar knowledge everyone should have it.” (Mental health worker, woman, Colombo)*  *“Especially, we have gone through this pandemic, Covid and all that. Nobody comes and tell that they have a mental problem. But, you know, many people have that. So that's kind of an enlightenment, you know, kind of workshops in rural settings.” (Religious leader & school principle, man, Colombo)*  *“Then I think that would be very good. Because I would say a lot of the teachers in Sri Lanka also need to know about like awareness about these things. And it's funny because a lot of the times in Sri Lanka, a lot of the reasons for depression comes from the education system and how teachers and grading” (IT worker, woman, Colombo)*  *“But of course, if there are students in the school who don't know that they have a mental health problem, then the teacher comes and talks and explains things like that, it could help them understand the situation.” (Nanny & household help, woman, Badulla)*  *“So what he says is that he thinks that this approach is a good thing. And he says it has two-fold. The fact that teachers are trained, it's a good thing. And the fact that the children are getting awareness is also a good thing, he believes. Because in Sri Lanka, he says that he sees mental health problems from the childhood level. So he believes that this kind of approach cannot just benefit the teachers to be educating children but for the children themselves to get a better understanding about their psychology.” (Police officer, man, Badulla)*  ***“****So what he's saying is that because the number of people who would voluntarily go and reach out for mental health assistance in Sri Lanka, providing education and awareness to students at a very young age will open up their minds to the concept of mental health and they’ll be able to understand and therefore in the long run that would pave the way for reduced mental health issues in the population.” (Farmer, man, Badulla)*  *“So this would be really good. Bring a lot of awareness to them. […] Because they probably have no idea why they're even feeling all of these things. And just letting people know that, okay, this is a mental state that you're in and can be overcome. And just people talking to you about it will help them a lot for sure.” (Freelance writer, man, Colombo)*  *“Yeah, it's a good strategy because that is the primary thing. That is the basic things the people, the children are getting from school. So from the beginning, if they can get the details, can get educated from the teachers, that will be a great opportunity. When they have a problem, they can face it without any difficulty because in their basic life, in their primary education, they would get more detail from the teachers. It's a great thing, I think. It will be accepted by the government or the education system. That's great.“ (Banker, man, Badulla)*  *“So by educating them and by it's being normalizing, it'll be a great opportunity to students and the people to learn what is the condition they have, and they are presently feeling or facing the problems.” (Banker, man, Badulla)*  *“The first thing is very important, because the primary education should be introduced to schools through the teachers. And by educating the people, the children these mental conditions will be normalized and they will understand the real situation. He or she is not a crazy person. It's just a mental condition. It can be treated and it's a normal thing. And from the patient perspective, also, they will understand this is a situation. I should have the medication. And I should have the consultancy specialized person that will be great to introduce as a mental health condition subject in Sri Lanka education system” (Banker, man, Badulla)* | | Statements in which participants see the benefit in increasing the mental health literacy when implementing the school-based educational outreach intervention. |
| 2.4.2 Beneficial reducing the mental illness burden | Participants see the benefits of this school-based educational outreach approach in reducing the overall mental illness burden, because…   - Intervening in young age through educating and raising mental health awareness can help to prevent illness development in the future. - There is an increased mental distress in school students, due to the COVID pandemic, increased academic pressure, and high rates of drug addiction among youth. | *“It's very good, it's a needed thing. There are a lot of people from students who have a lot of problems. In Sri Lanka, depression is there, and harassments are there, drug addicts are there, the poor people are there. So, it is a must. So we need to get teachers, we just have the [inaudible 00:08:33] to do counseling because they always know the students. Some teachers got the training and graduated to do such things. So, they are very good.” (Mental health worker, woman, Colombo)*  *“Especially, we can pick mental health cases in the early childhood, we can get some good intervention.” (Mental health worker, man, Badulla)*  *“So we can prevent a lot of children developing psychological issues in future.” (Mental health worker, woman, Colombo)*  *“But then again, if there are too many children who need support, especially after this Covid pandemic, we have seen lot of different children in our schooling system. They look very fine, but then they're not settled. So actually, this is a time that we need so many counselors in our schooling system, but we don't have the luxury for that.” (Religious leader & school principle, man, Colombo)*  *“There are a lot of people from students who have a lot of problems. In Sri Lanka, depression is there, and harassments are there, drug addicts are there, the poor people are there.” (Mental health worker, man, Badulla)*  *„It has to be in the schools, because that is the beginning. If we are to find whether there are any concerns with the child in early stages, that has to be done in the school.” (Religious leader & school principle, man, Colombo)*  *“So a lot of the suicide rates and everything here is because kids a lot of kids in schools, I know local schools. So there's international schools and local schools. Local schools have had suicides because of teachers and their parents; them not being able to tell their parents that they passed or that they failed. And because of that, they think that the means of taking their life is easier than coming to their parents with these grades. And a lot of times the teachers also, yeah, they put a lot of pressure in it as well. So I feel like if they're more aware, it would be a big help because then they can teach it to the students as well.” (IT worker, woman, Colombo)*  *“He believes that it's actually quite useful because right now they are receiving intel that there is an increase especially in drug abuse among students in Sri Lanka.” (Police officer, man, Badulla)* | | Statements in which participants believe that implementing the school-based outreach intervention would lead to a decreased mental illness burden. |
| ***Type 5: Non-specialist mental health interventions involving digital technologies***  ***Definition: Any involvement of any type of digital technology in any non-specialist mental health interventions.*** | | | | |
| 2.5.1 Beneficial for making the intervention more attractive | Participants believed that the benefit in involving digital technology in any non-specialist mental health interventions is that it makes such interventions more attractive, because it would generally raise the interest in such interventions through for example the usage of visualisation tools (i.e., videos, power points), or usage of social media platforms. | *“I think it can be accepted likely, because kids like to be on the phone and that's interesting for them.” (Mental health worker, woman, Colombo)*  *“Everybody's on TikTok, even everybody that's illiterate is on TikTok. TikTok has amazing penetration in Sri Lanka, everyone is on TikTok. There is good engagement with the user interface, but it's about literacy again, I would say” (Mental health worker, woman, Colombo)*  *“Yeah. Because like, say, group of people, you show them short film like thing to educate them. It should work, definitely. Yeah. Also, if you have, say, social media. I mean, if you could create a website or something like that for you to go online and take advice and things like that. It should work, I think. Especially, for these young children, they are very text heavy and all.” (Banker, man, Colombo)*  *“Because now, you know, people are drawn to technology. Right. That is one thing. But then I'm saying this because we live in city areas. But in the rural setting also, they welcome this knowledge because they have all heard about it.” (Religious leader & school principle, man, Colombo)*  *“So, you know, the moment you tell those people there is this, you know, technology that we use to assess you. So through that we will be able to get you, people will like it. Because people know, even though they don't know how to use it, they know how advanced the technology is. Even in the rural setting, they know that. So that will be kind of, you know, a good thing actually.” (Religious leader & school principle, man, Colombo)*  *“Yeah, they would accept it. So what I was saying is that even our parents now, even they are old, they also see the internet and they also go through social media. So they are also little bit more aware of these things.” (IT worker, woman, Colombo)*  *“I think you can show a lot of videos, for sure. That would be more interesting to the kids than just someone there talking, explaining it. Would be just another lesson for them. So definitely technology can be included to become more interactive and for them to relate more to situations.” (IT worker, woman, Colombo)*  *“It definitely can play a huge role because obviously of all the information we can find on the internet and just showing people examples of it, like is a lot better than just telling them about it. […]* *I'm sure like even the people in the rural areas, it would be a lot cooler if you had like a digital presentation for them. They'd be pretty amazed by it also” (Freelance writer, man, Colombo)* | | Statements in which participants expressed that involving digital technologies in any non-specialist mental health interventions would make such interventions more attractive for the users. |
| 2.5.2 Beneficial for facilitating access to services | Participants believed that involving technology in non-specialist mental health interventions can increase the general access to services by addressing the poor infrastructure and the discomfort of physical meetings with mental health providers through:   - Providing online training to non-specialists - Providing remote supervision meetings - Providing remote mental health education services for the public on e.g., (social) media platform - Providing remote care (asynchronous or synchronous) | *“One of the things that I've been working on or designing is that everything should be run on Zoom calls or something like that. So when I'm talking about it the resource person should have access to monthly or weekly Zoom calls, so that they can flag up the issues that they are not equipped to handle, or that they have a question about.” (Mental health worker, woman, Colombo)*  *“We could do this training online too if someone wants to join from home via Zoom or something like that. We can help them out with a lot of presentations and YouTube videos or TED Talks, or whatever it is. We can use them and give them better training.” (Mental health worker, woman, Colombo)*  *“The supervisor can monitor online, so that opportunity is there even over the phone” (Mental health worker, woman, Colombo)*  *“And then, we can create so many interventions, we can create so many assessments through computer or something, and we can share with them so they can participate in those things like that.” (Mental health worker, woman, Colombo)*  *“As I said, social media platforms and other platforms are also useful to deliver the message to people in a short period. (Mental health worker, woman, Colombo)*  *“So, if they can introduce some application, some app to contact such a person will be beneficial. SMS system or hot number, hotline number to contact the social worker or that person. If you give that number to the community, they have a problem, they will give you a call or leave a message.” (Mental health worker, man, Badulla)*  *“Actually, the first one is for training purpose. It's better if you can use technology because of the physical meetings it takes a bit -- it's okay. Physical meetings are very interesting, but to reduce the traveling times and also to catch the very feasible times for the first integration […]And also we can use this technology to monitor how they offer their services. For example, if they are counseling. Or when a teacher is having some discussion with the child regarding a case of their mental health. With the consent of all the parties, we can project into this from somewhere else.” (Mental health worker, man, Badulla)*  *“So we need more organizations, more people providing free mental health service, which is accessible. So, for example, even if you take [NGO name], we have clients who are working even in their middle ages. Like in their 20s, they cannot pay clinic. They cannot pay to be seen, even if they want to go to a psychiatric clinic. It doesn't make sense. It's not possible for them to get a day off and go and stay on the clinic from morning and then see psychiatrist. Go to the clinic, get a medicine. It doesn't work. It's not effective that way. So what we provide in terms -- I mean, the whole online counseling service we also started providing from with the whole Covid lockdowns. There were clients who would take the lunch hour, go to a conference hall, open the laptops, do their sessions, they get back to work. It's more accessible and it's free of charge.” (Mental health worker, woman, Colombo)*  *“He believes that technology has a very important role to play whether it's education or awareness or providing assistance” (Police officer, man, Badulla)*  *“And I would kind of want the social worker to -- I don't know. Maybe I can call them, maybe I can just like video call them, like kind of more freely than the specialist.” (IT worker, woman, Colombo)*  *“So she thinks that technology can have a positive impact, especially in terms of making sure that different teaching communities get like the training and development and resources that they need. So she feels like technology and come in handy on that respect.” (Midwife, woman, Badulla)*  *“She believes that the biggest role that technology can play in this particular approach is helping to update and up skill the care providers who are enrolled in this particular approach. So give them access to online courses to online resources, make them more tech savvy so that they can grow and develop their knowledge and she feels that that is very important.” (Midwife, woman, Badulla)*  *“So she believes that it will help with communication in this sense like for example if we are going to get the help of a licensed professionals in mental health, we can use technology to connect the grassroots people directly to them so that absorbing the information that they can have becomes a more efficient process. So communication.” (Midwife, woman, Badulla)*  *“She thinks it's good to use technology because anyway, given the trainings is anyway beneficial to people and they will be able to gain a lot of knowledge. So she thinks it's a good idea to give this training to people using technology.” (Nanny & household help, woman, Badulla)*  *“And I would kind of want the social worker to -- I don't know. Maybe I can call them, maybe I can just like video call them, like kind of more freely than the specialist. If that make sense.“ (IT worker, woman, Colombo)*  *“I feel like communication is just like a big thing. Because I don't know that sense of like that person will be there kind of even after hours. I don't know if that's a thing, but I do know some people here actually who WhatsApp the therapist. I don't know if that's like allowed. But I know they do. I know even odd hours they can send that therapist a message if they need something more. And also, I know therapist who also WhatsApp people off their hours and just ask them, you know, are you here? Are you doing that thing I asked you to do? Or how are you doing? Are you okay? Stuff like that.” (IT worker, woman, Colombo)*  *„So, if there are teachers who are interested in this kind of program, then they will be able to use technology to join and get that training and get that information.” (Teacher, woman, Badulla)*  *“Now a lot of people have turned into online more as well. So I think with the online thing, a lot of people are reaching out psychologists now. They feel comfortable talking online than coming to a clinic physically. “(Mental health worker, woman, Colombo)* | | Statements in which the involvement of digital technologies in any non-specialist mental health interventions would facilitate the access to such interventions or other required services (i.e., for the non-specialist). |
| 2.5.3 Beneficial for facilitating care coordination | Participants see the benefit in involving digital technology in terms of care coordination, specifically for:   - Maintaining and sharing data - Scheduling appointments with clients | *“[…] and even the documentation part you can update the documents in a drive or something so that it's live and it's visible to your supervisors also have access to it so that they tell. So that that part you can do.” (Mental health worker, woman, Colombo)*  *“we usually document the thing, then we usually maintain a personal file. So that could be enough. At the end of the session, if you see anything alarming. If you have proper concerns, you can just mention them, write them down, and then send them to your supervisor so that you get a better idea of them.” (Mental health worker, woman, Colombo)*  *“You have apps where you can make appointments online and see, consult your psychologist online. So that's happening. So maintaining that or improving those might help.” (Mental health worker, woman, Colombo)*  *„Having like a centralized way how you can maintain notes, or have client details, or have diagnosis, or where even the therapist and practitioners have ease of access to whatever they need. Or technological platform may make it easier, because even in Sri Lanka use these stacks of files which you have to hunt out for each patient. So, which makes it more complicated and a lot of writing. And a lot of, I will say, paperwork that needs to be done.” (Mental health worker, woman, Colombo)*  *“Of course, you can do some sessions with the supervisor, and then keep things online or over the phone. But I think it's better if they’re talking and sorting things out on the same day. Rather than taking two or three days apart because sometimes you might also forget what you did and how you did it. Documentation is very important. The trainee has to document everything, including the client’s details and the like to maintain the personnel files properly, when you're talking to your supervisor or the specialist and there are things that you don't remember, you can simply open the documents and discuss them. “(Mental health worker, woman, Colombo)* | | Statements in which the involvement of digital technologies in non-specialist mental health interventions would facilitate the coordination of care in any way. |
| 2.5.4 Beneficial for facilitating mental health awareness programs | Participants believed that technology can help to facilitate mental health awareness programs by visualising that others have mental health problems too (i.e., through videos) | *„when it comes to for example, if it's an addiction awareness program, or addiction awareness intervention, then we play a video for the awareness, it's more effective for them. If they see a video or something, if they see real cases, real experiences, when it comes from the video or audio, it will be very effective for them.” (Mental health worker, woman, Colombo)*  *„I think the only step that you can do through technology is just like ways to improve awareness, maybe even like short clips on how to-- short clips on what is mental health? Why is it important? What can go wrong if you don't pay attention to your mental health? Global statistics of what are the things that are going wrong because of mental health issues and things like that?” (Mental health worker, woman, Colombo)*  *“Technology. Again, it's just shows people that there is so many other people in the world going through things like this everywhere and not alone. And all the people that -- you can show all the people that have overcome all of these things and stuff like that. And that would make them feel like, okay, there is hope for me. And just videos and stuff like that, make content. You can go through a lot more content in those formats.” (Freelance writer, man, Colombo)* | | Statements in which participants expressed that digital technologies can specifically facilitate the delivery of non-specialist mental health awareness programs. |
| ***Theme 3: Important non-specialist characteristics to increase acceptance***  ***Participants identified important characteristics that a non-specialist should present in order to make such interventions more acceptable*** | | | | |
| **Type 1: General non-specialist mental health interventions**  **Definition: The general concept of involving non-specialists in any mental health interventions.** | | | | |
| 3.1.1 Importance of good social recognition and status | Participants emphasized the importance of selecting people with good social recognition and status as non-specialists, as this increases the trust towards them. | *“So, when a community person is doing psychological things and the people go to meet him the entire village will know that person is going there because he or she has to be accepted by the community.” (Mental health worker, man, Badulla)*  *“In my local area here, in my temple, it has this lady […] I had identified that those are the kinds of people that I want to recruit [as non-specialists] because they're the people that anyway if there's a problem within the community people tend to turn to […] She's a single lady and she's middle-aged, but she likes people. She likes the temple. She likes to sweep it, keep it clean, feed the dogs, and watch the dogs, she's involved in the temple activities.[…] Because of that, she knows the entire community and this entire area she knows like the back of her hand. So those are the kinds of people we have to find, but in different communities, that person might be different.” (Mental health worker, woman, Colombo)*  *“I think like now, as I told you earlier, we have midwives here who goes and checks about these pregnant ladies and their babies. A person like that would be ideal. […] And that would be definitely ideal. They have built trust among people”(Banker, man, Colombo)*  *“Teacher and students. […] Because they are more respected in Sri Lanka” (Banker, man, Colombo)*  *“That is, of course, after the social worker build up her personality or his personality. And he has to get the confidence of the community” (Banker, man, Colombo)*  *“And people have confidence in them. When they come, they know that they are in safe hands. But they are not qualified doctors or midwives. If the community has trust on this person, the local person that you appoint and train, whatever, then of course they'll even come to them and talk. Again, that person has to build that personality in the village.“ (Religious leader & school principle)*  ***“****So she says that because clergy and religious leaders have such prominence in Sri Lankan societies.” (Nanny & household help, woman, Badulla)*  *“He believes that it would be good to appoint people who have some form of social recognition that people will feel confident in approaching.” (Police officer, man, Badulla)*  “He also believes that it's good to have policemen and women involved because again, they can help people, people accept them. There's a lot of social acceptance. So yes.” (Police officer, man, Badulla)  *“Now, the challenge here is that unless this person, the non-specialist caretaker has been given the position of social acceptance and maybe even authority to a certain extent, the chances of people going to them and talking to them and asking for help is going to be very, very low because in Sri Lanka that that acceptance and recognition for mental health is not there.” (Police officer, man, Colombo)*  *“So it's important in his opinion to always choose a person who has that level of social acceptance or otherwise put them in a position where they will be readily accepted by the people if this is to work.” (Police officer, man, Badulla)*  *“So he says of course, this would be another good strategy but only if the training is provided to people who would be readily accepted by the public and the society in Sri Lanka. […] Providing this training to just anybody in the community he thinks it will not work because people will not feel that…the recognition, the qualifications, they will not feel like they can be trusted.” (Farmer, man, Badulla)*  *“So what he's saying is that if it is possible for us to like approach people who are in various levels of social recognition. He is not giving a specific position or designation, but people in levels of social recognition it might work well with the communities.” (Farmer, man, Badulla)*  *“Let's say he has to be very impartial and socially accepted person.[…] The person who helps the others, and with them and our need. So who should have a kind of a figure, right? So I mean figure in a sense. One who is not cheating people. The alpha man and the manliness and people will approve.” (School principle, man, Badulla)*  *“midwifes. Because they're constantly in contact with people in their communities and they're always going into the community it will be easier for them to come into contact with people and offer them services.” (Teacher, woman, Badulla)*  *“[…] maybe religious leaders can also be trained because people always trust and follow religious leaders in Sri Lanka and they have like this kind of close rapport with their communities.” (Teacher, woman, Badulla)* | | Statements in which participants state that a good social position/status/recognition is required for a non-specialist to be accepted by the community. |
| 3.1.2 Suitable occupational groups | Participants identified midwives, teachers, social workers, religious leaders and female police officers as suitable occupational groups for being non-specialist mental health workers, because of…   - The increased trust and acceptance within the society - The close rapport with the community - Increased interest and motivation in being a non-specialist | **Midwifes:**  *“And I think already Sri Lanka, I think you are aware of this, our midwives. And midwives are very good source because that is the only government officer who can go to the bedroom of a woman and sit on the bed and talk to her. Right? Because she can go, nobody can say no to her because that is her duty. To go and talk to the woman and look into the children's health and all. […] They know what are issues and they know dynamics in the village and all. And they may be already have identified hot spots in the village; maybe substance abuse and other issues. So they might be able to do a better job, rather than totally new person from outside come and do it.” (Mental health worker, woman, Colombo)*  ***„****I think like now, as I told you earlier, we have midwives here who goes and checks about these pregnant ladies and their babies. A person like that would be ideal.[…] Because like they have some sort of medical background also, in that if you could educate them on these. Provided they have time to do it. And that would be definitely ideal. They have built trust among people; midwives in these villages especially. So it'll be ideal if you could. I mean, provided they have time.[…] Midwives are also respected.“ (Banker, man, Colombo)*  *„So she says that the advantages of this particular grassroots level program is that especially in her profession, as a midwife. She already is engaged in a role where she's constantly communicating and maintaining relationships with the people in that she's providing services to. And because of that, there is always a very personal sort of mutual understanding relationship that has that kind of comfort zone, which allows these people to come out and communicate openly and freely with all about whatever mental health related conditions that they might be facing. So that's definitely an advantage in her opinion“ (Midwife, woman, Badulla)*  ***Teachers:***  *“He thinks teachers are a good option because they can move easily with students and also because they are well accepted everywhere.” (Police officer, man, Badulla)*  *“Because teachers are the ones who flow. They work very closely with the student. So even the behavioral changes, even if it's a slight change, the class teacher or the subject teacher could recognize that.” (Mental health worker, woman, Colombo)*  *„Teachers are the basis for everything because students are learning more from the school. So, if the teacher knows psychology and counseling, if the teacher knows how to deal with kids and everything, if the teacher is emotionally intelligent and she gets good proper training, then it will work out, in my opinion. Because she's the one who is going to teach right and wrong to the students. Yeah, that would be fine. That will work out, in my opinion“ (Mental health worker, woman, Colombo)*  *„So we need to get teachers, we just have the […] to do counseling because they always know the students.” (Mental health worker, man, Badulla)*  *“These specific teachers are very committed.[…] And teachers are the first encounters, so they can identify the problems early as possible. And they can refer these children to psychiatric clinics and psychologists once before the children develop.” (Mental health worker, woman, Colombo)*  *“I think it's a very good approach, because teachers do come across cases like this. If they're educated to handle cases, then it'll be good thing if you could implement this. Because teachers, they teach for about, say, in a class in Sri Lanka, we have about 35 to 40.  If she or he goes to maybe four or five classes, he is covering number of students. So I think this is a good approach.” (Banker, man, Colombo)*  *“Because her point of focus being that apart from parents and apart from their households, children spent the most amount of their time in schools and therefore teachers are in an ideal position to help children out and to disseminate information among children.[…] In addition, she also thinks that the teacher needs to play an extremely important and very prominent role because after five years of age according to her experience, children really form deep connections with their teachers and sometimes they tend to listen to the advice and counsel given by their teachers even more than they listen to their parents. So if the teacher can play a prominent role, she believes that this will be successful.” (midwife, woman, Badulla)*  *“He thinks teachers are a good option because they can move easily with students and also because they are well accepted everywhere.” (Police officer, man, Badulla)*  *“I mean, that's amazing actually, because when we were in school, it was a long time ago, but we had nothing like that. We didn't even have like counselors or like anything in school. Like, literally no one. If you already talk to someone, it's just another teacher. Like there is no special person.“ (Freelance writer, man, Colombo)*  ***“****She thinks they will be okay [that teachers work as non-specialist]. Yes.” (Teacher, woman, Badulla)*  ***Social workers:***  *In that perspective, she believes that this approach can operate relatively smoothly and that like, for example, social workers and grassroots level workers will be very willing to learn how to listen to what something a psychology professionals or a counselor or therapist or a doctor would tell them they would readily listen. (Midwife, woman, Badulla)*  *“And then the social workers, there are social activists who would like to play this role, so we can get help from them too.” (Mental health worker, woman, Colombo)*  *„There are social workers who are dying to do something good for society, we can take them.” (Mental health worker, woman, Colombo)*  ***„****So social workers for sure. And I think people working in like NGOs and do like charity work, I would say. Who deal with like one of the populations.“ (Mental health worker, woman, Colombo)*  *“In that perspective, she believes that this approach can operate relatively smoothly and that like, for example, social workers and grassroots level workers will be very willing to learn how to listen to what something a psychology professionals or a counselor or therapist or a doctor would tell them they would readily listen.” (Midwife, woman, Colombo)*  *“but I think a social worker should be someone who like is a normal person of the community” (IT worker, woman, Colombo)*  ***Religious leaders:***  *“So she says that because clergy and religious leaders have such prominence in Sri Lankan societies. And because people will automatically go and ask them to chant threads or carry out religious rituals to help their children who have mental afflictions. It's possible that if training is given, maybe these clergy people will be able to help these people more not that these people will go and ask for mental health assistance, but maybe while they're doing the ritual or whatever, they will be able to help them in one way or the other.” (Nanny & household help, woman, Badulla)*  *„But definitely, the temple would know quite a lot of people. The temple would have the understanding about the community that maybe the GP lacks[…]But I know that in my local temple where my children go to Sunday school, there are people inside the temple, the lead congregation person or some layperson who is always the heart of the community.“ (Mental health worker, woman, Colombo)*  ***“****I feel religion is quite high, like important here. So I feel like, yeah, definitely people like priests, monks and stuff, if they are educated more on like how to deal with mental illness and leave little bit of the religion behind, yeah. I feel like people would trust that more; religious people more like priests and monks or something.” (Mental health worker, woman, Colombo)*  *“Maybe Buddhist monk, because majority is Buddhist in Sri Lanka. So Buddhist monk would be. All right.” (Banker, man, Colombo)*  *“But yes, religious personality. I know Anglican clergy who are serving in rural areas. They do that. They visit people. If there is a situation where they need the support of the clergy, they go and sit there and speak to the family or to an individual. They do that.“ (Religious leader & school principle, man, Colombo)*  *“In addition to that she also believes that maybe religious leaders can also be trained because people always trust and follow religious leaders in Sri Lanka and they have like this kind of close rapport with their communities.” (Teacher, woman, Badulla)*  *“It's normally in Sri Lanka, most of the people are religious, right? They have a religion. And when they have a problem, always they're supposed to go to a priest or a father or a monk. They'll express their experience with them. So they'll get advices; most of them are getting advices from these kind of religious people. So if we can educate them as instructors or advisors, that will be also all right.” (Banker, man, Colombo)*  *“I know I can give you an example. When I was serving at [name] Church, I did that in [village]. They appointed a group of lay people to visit houses, especially where there are elderly people, to go and talk to them, spend some time with them, that kind of thing. But they were not trained actually. We had that checking there.” (Religious leader & school principle, man, Colombo)*  ***Police officers:***  *„[…] but police officers, especially the lady officers, because they also deal with a lot of disturbing things in their jobs. They move with every aspect of society, every social class, and all that. I think they also have a bigger scope into society and what goes around. They have that experience and exposure, so we could train them too.” (Mental health worker, woman, Colombo)*  *“He also believes that it's good to have policemen and women involved because again, they can help people, people accept them. There's a lot of social acceptance.” (Police officer, man, Badulla)*  *“[…]so he's speaking about the Sri Lanka police departments. What he believes is that it's important to have a well-tuned methodology of selecting officers who are capable of taking this training on. So they'll have to implement first a system of shortlisting candidates who are suitable to take on this training and who are willing to participate as well. And then if we give them this knowledge, they will absorb it well and they will carry it forward and they will help in the implementation of such programs.” (Police officer, man, Badulla)* | | Specific occupational groups that are considered as suitable for being non-specialists. |
| 3.1.3 Suitable personal characteristics | Participants identified what they perceive as important personal characteristics for non-specialists:   - Good soft skills (i.e., communication) - Good coping and self-awareness skills - Being similar to the client in terms of demographics (i.e., age) or past experiences (i.e., relating to the mental health problems). | *“I feel like teachers [non-specialists] also need to go through training that requires them to be introspective and identify their personal biases and deal with them. […] I think that's important because at least as a psychologist, we have to work on that a lot. We have to deal with people with a lot of problems, and most of the time, we deal with people who are on the receiving end of problems, but also, we deal with the person who is the problem sometimes. And that's very difficult because we have our own triggers, we have our own trauma, we have things that happen to us, and we have to put those aside and not have those biases against our client, even though our client may be the wrong person in that situation. Your client can be the abuser in a relationship, your client can be the enabler of a toxic relationship. Your client can be in a type of relationship that maybe you don't agree with, but you have to put that aside and create the best possible plan and approach for that person regardless of what sins they may have committed as a part of that problem.” (Mental health worker, woman, Colombo)*  *“But in terms of grooming young people, I would say people who you could do a personality check on. And do like shortlisting people with better coping strategies. I would say interview a couple of people who are interested, number one, to do the support. And then based on doing personality checks or doing like I would even say a mental health assessment to see if they are the right client to provide that support.” (Mental health worker, woman, Colombo)*  *„She already is engaged in a role where she's constantly communicating and maintaining relationships with the people in that she's providing services to. And because of that, there is always a very personal sort of mutual understanding relationship that has that kind of comfort zone, which allows these people to come out and communicate openly and freely with all about whatever mental health related conditions that they might be facing.” (Midwife, woman, Badulla)*  *“It should be the person that the patient feels is relatable to, I think. I mean, a lot of them are actually older. So I would really wouldn’t want to maybe talk to an older social worker and we might want to talk to a younger one.” (IT worker, woman, Colombo)*  *“I would prefer if the social worker and I will could talk freely. Something like that.” (IT worker, woman, Colombo)*  *“I see some -- you know, I have that ability to speak to children with this limited understanding they have. But some really don't have that.” (Religious leader & school principle, man, Colombo)*  *“So like I said, they don't really have to have a knowledge, but they just have to have the empathy and like how to talk to the people, because of the state they're in. Those kinds of soft skills.[…]* Or they have to have a compassionate person.” *(Freelance writer, man, Colombo)*  *“Or they have to have a compassionate person.“ (Freelance writer, man, Colombo)*  *“I feel like a non-specialist person, just because of all the things I've had to listen to with like all my friends, you know. I just feel like I've had a lot of these experiences before. I don't know, I guess I'm matured a bit faster than most of my other friends and went through quite a bit of things early on. So now that I see them going through the same things, like it's just easy for me to advise them on how to move forward with things.” (Freelance writer, man, Colombo)* | | Specific personal characteristics that are considered important for a non-specialist. |
| 3.1.4 Higher academic status | Participants believed that people with higher academic statuses would be more trusted as non-specialists. Particularly a general medical background was preferred by the participants. | *“I think someone should at least have master's, because I don't think that's just enough. I don't think I'm ready to give counseling to anyone.” (Mental health worker, woman, Colombo)*  *“If you have medical background, that would be ideal. But I don't think in Sri Lanka you could find people who have medical background” (Banker, man, Colombo)*  *“And also anyone who is educated can be trained like well educated because she thinks in order for someone to understand the situation they need to have like a good education as well.” (Teacher, woman, Badulla)* | | Statements relating to a (specific) educational status for non-specialists. |
| ***Theme 4: Suggestions to facilitate reach, acceptance and feasibility***  ***Participants identified important practical considerations on different levels for the successful implementation of general non-specialist mental health interventions and specific sub-types.*** | | | | |
| **Type 1: General non-specialist mental health interventions**  Definition: The general concept of involving non-specialists in any mental health interventions. | | | | |
| 4.1.1 Advertising interventions | Participants emphasized the importance to advertise non-specialist interventions through:   - Word of mouth starting from specific groups (mothers, religious community) - Social media | **Word of mouth:**  *“At the same time, I would say, in any society, if you can advertise through to the moms, you create a wave of change that does change society, because if the moms have the knowledge and the skills, if the moms have good mental health, the rest do tend to follow. So, it might be a really good place to start.” (Mental health life coach, woman, Colombo)*  *“[…] it could prove to be effective even if it goes through word of mouth. Like, oh, there's counselor down the road, and she's very good at listening to my problems. And why don't you go and give it a shot? So it may help.”* *(Mental health worker, woman, Colombo)*  *“And if that is helpful, then that person will go back to their community and then word of mouth, “This particular priest is really good. This person did this with me and it helped.” That kind of like snowballing can happen is what she's saying.” (Nanny & household help, woman, Badulla)*  *“[…] use places of religious importance to kind of distribute this idea about non-specialist mental health among people to initiate mental health programs and education awareness programs using places of religious importance as the distribution center” (Farmer, man, Badulla)*  **Social media:**  *“Other than that, people are more into social media these days, when it comes to advertisements, we choose Facebook, WhatsApp, or Instagram which will be reached so easily.” (Mental health worker, woman, Colombo)*  *“What she has noticed is that there are organizations available in Sri Lanka like Sumithriayo which are advertising on social media and in the form of posters on vehicles and stuff like that.” (Teacher, woman, Badulla)*  *“She also says that in terms of television programs, she has noticed that in certain television channels in the morning of us there are programs where these organizations like Sumithriyao etcetera, etcetera, they come and they carry out like talk show sessions and stuff to create awareness and educate people” (Teacher, woman, Badulla)* | | Statements in which participants express how /that non-specialist mental health interventions should be advertised. |
| 4.1.2 Community recruitment | Participants believed that to increase access to such interventions, non-specialists need to approach the service users or recruit from the community. | *“So he believes that there is a lacking factor in Sri Lanka because the mental healthcare people are waiting these people who want the non-specialists, they are waiting for people to come to them and then they assist them but they don't reach out by themselves.” (Police officer, man, Badulla)*  *“[…] the chances of people going to them and talking to them and asking for help is going to be very, very low because in Sri Lanka that that acceptance and recognition for mental health is not there” (Police officer, man, Badulla)*  *“What needs to happen is that locally trained person needs to go to those people.” (Religious leader & school principle, man, Colombo)*  *“So actually, around school, this is another. I think this has already gathered audience. If you can reach mothers who come to school to drop their children, sometimes they stay around the school until the child finishes their classes in school. Proportionately huge number actually. If you can use this waiting time. They just chat and group and those things. There are so many other issues they go into like extra-marital relationships and so many issues, because they have a lot of time with no purpose until their children come out from the school. All the way to school. Sometimes even they don't stay throughout. They might stay one hour near school and they come home and then go and pick the child again. So if you can reach those mothers, especially parents during that time through a mechanism, that would also really be a very helpful.”(Mental health worker, woman, Colombo)*  ***“****I think going over to that person, especially if that person -- I mean, if only the patient like the social worker. And if they're okay with it, I think it would be the best for the social worker to go to a place that the patient is more familiar with. And they create like sort of like unprofessional bond before.” (IT worker, woman, Colombo)*  *“Because I wouldn't feel more comfortable if they came over. I know some people who won't. So that definitely depends on the person.“ (IT worker, woman, Colombo)* | | Statements in which participants explain that/how community recruitment should take place. |
| 4.1.3 Ensuring privacy protection and gaining trust | Participants emphasized the importance of ensuring the privacy protection in such mental health interventions, by for example   - Ensuring the privacy, by for example “hiding” the care setting - Ensuring data security | *“But you could tell, you could instruct teachers and then they can, you know, be without divulging information to parents. They could look after and solve their students’ problems.” (Banker, man, Colombo)*  *“If it is in the community also the people will not go into that office or clinic because that person is particular, they know that he or she is doing psychological or mental health issues, dealing with mental health issues. So, when a person goes to meet that person, everyone knows that he's going there because he or she has some mental health illness. […] In the hospital setup, some other counters that help other women, there are such things in a small place in the hospital. If it is situated in the hospital, the patient, the person will go, they can go there, they know that they are going to the hospital no one knows” (Mental health worker, man, Badulla)*  *“The advantage is if they can manage the secrecy, if they can manage the patient with the secrecy, that will be a great thing.” (Banker, man, Badulla)*  *“We don't have to use the word the mental condition. If we can name the two people, like doctor and non-specialized person, we can name with another name. I mean, I'm trying to emphasize the fact that if it is not a mental hospital or mental clinic, that will be a great help to students and the patient. They have to use another word for this this condition.” (Banker, man, Badulla)*  *“It [the non-specialist care setting] should be a normal clinic. Normal dispensary. In Sri Lanka, it should be a normal dispensary or clinic. With that mental condition, other patients can. Also if they can go to the same place, that will be a great thing for the people who are having these mental conditions.” (Banker, man, Badulla)*  *“[…] but like I would feel more comfortable only if that privacy is there.” (IT worker, woman, Colombo)* | | Statements in which participants express that/how privacy of the service user should be warranted to gain the trust towards such interventions. |
| 4.1.4 Payment and incentives | Participants emphasized the importance of regulating the payment for such services and providing incentives for the non-specialists:   - Affordable services for service receivers - Fair payment or incentives for non-specialists to increase retainment of non-specialists and trust in service users | *“And I'm guessing if this person is not medically trained, or in that way trained, it would be either free or it will be financially more accessible. Both of those things combined can be very attractive” (Mental health worker, woman, Colombo)*  *“And some people don't like to just spend that energy on training, because they would rather think, “Okay, I can go somewhere and a session or something and earn. At this time, I can earn, why would I spend it on training someone?” And when you train someone, of course, even later, there are five patients. So, if there's someone you're training, you will have to give at least two patients to the newcomers. So then, of course, that also gets divided. So if people are paying for that session, their income also gets reduced. So these concerns might also be there.” (Mental health worker, woman, Colombo)*  *“Here in Sri Lanka, insurance doesn't cover anything related to mental health. Counseling is not covered. Counseling is pricey here in Sri Lanka, it’s expensive. In this Stepped approach, if they have to spend more money, two or three times, they might not go for it. But if it's sponsored, yes, they will.” (Mental health worker, woman, Colombo)*  *„If I had a doctor who is doing surgery on me for free, I would be very worried. Because I’ll be like, “Why are you doing this? This is such an important procedure, I could die. You need to get paid because I need to make sure you are trained and you're doing a good job because every job needs to be paid for.” Having volunteers do something that is so emotionally distressing. That could easily even be a trigger for this volunteer which will again impact their performance when dealing with someone who is in such a mentally distressed state at that moment.“ (Mental health worker, woman, Colombo)*  *“I think that a lot of the people here won't want to pay. So like therapy here is pretty expensive as well. So that's why actually a lot of people I know don't also get therapy, because it might be more expensive. Because there's such a little like supplier, so that they have to charge high prices. And so if they're recommended to another specialist -- I mean, a non-specialist, like in this intervention and especially they're not trained, so they would definitely question why they have to keep paying that amount to the specialist.” (IT worker, woman, Colombo)*  *“And that should be connected to organizations that pay these professionals and make sure that they are also well taken care of to do their jobs right because if you are having volunteers who are barely trained, barely educated in psychology to help mentally distressed people. If I was in that position, even I would be so burnt out, and I’ll be so angry because I'm like, “Why am I putting myself through this when I don't know enough about it? When I'm not even getting compensation for doing this?” (Mental health worker, woman, Colombo)*  *“If it is paid workers that will be better, but they have a responsibility also. Volunteers, it is difficult to find this because of the current situation and all that, a very difficult time. Volunteers, if they’re paid, will be beneficial for them also.” (Mental health worker, man, Badulla)*  *“And if we have selection criteria and again, we have a method of paying them a salary or allowances whatever thing that is going to be an ideal scenario.” (Mental health worker, man, Badulla)*  *“And also the training must be coupled with encouragement and also maybe having some financial benefits, if that will attract people to our training.[…] So we have to offer them a good training, good remuneration and other benefits. We can optimize this condition to good level. […] Maybe not money, but any encouragement; certificate or something like that will be okay.” (Mental health worker, woman, Colombo)*  *“But one thing that we may need to take into consideration is like how we are paying these people. Because they are paid very less for the services that they provided, especially psychologists, psych counselors, in Sri Lanka. I know I can speak only for Sri Lanka. Whereas when you come out to European countries or countries outside Sri Lanka, you are paid actually much better. So people tend to opt for providing the services elsewhere, given the current inflation and all of that. So I think even at the village, wherever they are, I think they need to be compensated rightly for the services that they are providing.” (Mental health worker, woman, Colombo)*  ***„****Because like what I feel is now in Sri Lanka we are lacking jobs, you know? So maybe not for the thing that they're going to do. They will come to get employment. And then some may do a good job. Some may not. That's what I told you that to find professionals or people who are working to, I mean, educate them and give them the necessary education to handle these cases.“ (Banker, man, Colombo)* | | Statements in which participants mention payment regulations for such interventions. |
| 4.1.5 Collaboration | Participants emphasized the importance of collaboration with specific organisations or stakeholders in order to facilitate the implementation process and also to gain trust within the society:   - Collaboration with and approval from the government or with governmental institutes - Collaboration with widely accepted non-governmental organizations (i.e., WHO) - Collaboration or involvement of religious leaders | ***Governmental organizations:***  *“So you have to get the government approval and you have to merge this to the main system, mainstream. Then normally you can do it. Otherwise, you won't able to. Even for an NGO to work in this area, they need approval from the government that is special. […]But I think whatever you do, you have to go through the health system. That's what I feel. The health system in Sri Lanka is very strong and very difficult to penetrate. They don't allow.”” (Mental health worker, woman, Colombo)*  *“Technically, you would be looking at partnering up with the government clinic.” (Mental health worker, woman, Colombo)*  *“And also the government. There must be policy mission from the government for some institutions offering mental health service.” (Mental health worker, man, Badulla)*  *“But in Sri Lanka, people are very much divided by the politics. If this plan is branded as a UNP or vote tour, so they should trust.” (Teacher, man, Badulla)*  ***“****You have to touch the higher level of education people, the people who are ruling the education system in Sri Lanka. So then you have to touch them, and you have to insist them to introduce that kind of subjects to their syllabus also. Then that will not be a problem again.” (Banker, man, Badulla)*  *“I'm going to start from the top, I mean the health ministry people, they need to understand the psychological problems that are there, and they need to invest as early as possible.” (Mental health worker, man, Badulla)*  ***Non-governmental organizations:***  *“[…] connect with one of these NGOs who are working really strongly with the community. So I think Care International, Plan International, World Vision. There are so many NGOs who are very strongly bonded with the community. They have the trust. So through them you can reach. And there's one NGO called Family Rehabilitation Center in Sri Lanka. So they work in Northeast. So I think approaching one of those will be easier” (Mental health worker, woman, Colombo)*  *“But if you go through the ministry; health ministry and mental health directorate, that might work. So how people do is through World Bank, or go through WHO. They convince the ministry with the funds and then ministry take it up. But always psychiatrists have more control and other medical functions will have some sort of control over already. That is the issue. So you can't go to the village and appoint these people without the government approval.” (Mental health worker, woman, Colombo)*  *“So actually health ministry is concern about the people who've been already diagnosed, right? But if you want to work with non-diagnosed like community people. I think you have shortcut too like to approach them. So they're very concerned about who are already in the system, who have already been diagnosed, and who are already taking the services. If they have not been to any hospital or any clinic, then you can do anything with them, but still with some precautions. But that is if they have been identified and been treated in a hospital or have come to a clinic, then they think they are -- these people are their product or they are property, I would say“ (Mental health worker, woman, Colombo)*  *„You have to touch the higher level of education people, the people who are ruling the education system in Sri Lanka. So then you have to touch them, and you have to insist them to introduce that kind of subjects to their syllabus also. Then that will not be a problem again.” (Banker, man, Badulla)*  ***Religious institutes:***  *“Yeah. Now the clergy in Sri Lanka has a big say in the society. So this clergy, the Christian clergy, and almost all these types of people, should then be aware of the outcomes of this. And it has to be social cohesion, and a social discussion. With that, I think this would be possible. If not, it's not going to be. […] about two years back, Sri Lankan education authorities introduce a book to sort of talk about reproductive health, reproduction. And that should be taught. It's my personal knowledge, personal belief. But you know what happened? All the monks’ professors and all those ones, I mean, they have ideas they went against and that book did not go to schools.”(School principle, man, Badulla)*  *“[…] and also maybe use places of religious importance to kind of distribute this idea about mental health among people to initiate mental health programs and education awareness programs using places of religious importance as the distribution center.” (Farmer, man, Badulla)* | | Statements in which participants mention the importance of collaboration with specific groups when implementing any non-specialist mental health intervention. |
| 4.1.6 Tailoring to cultural norms | Participants emphasized the importance of developing interventions that are tailored to the specific cultural norms of specific communities:   - Considering different religious norms - Considering different languages - Considering different social norms and structures | ***Religious norms:***  *“And they need a clinician who understands the Islamic community and the struggles that they have, or the Christian community, or even the Buddhist community, or people who live in this area or that area like they have very specific family-related problems. So that's my only thing. […]” (Mental health worker, woman, Colombo)*  ***Languages:***  *“I'm writing Tamil content and everything to deliver to my area people because they can’t understand English because mental health promotion and mental health workshops are mainly in English in Sri Lanka.” (Mental health worker, woman, Colombo)*  *“First of all, the language” (mental health worker, woman, Colombo)*  *“finding people, making sure everything is trilingual” (Mental health worker, woman, Colombo)*  *“And also, if this program goes in English, that language barrier would be there for rural areas. So that is going to be a challenging factor as well, then you would have to speak in both Sinhala and English or have a translator, or someone for them to make it clear for them.” (Mental health worker, woman, Colombo)*  ***“****They will trust. However, if you don't speak in Sinhala in that area, in rural area, they will not understand English. So nobody will come.“ (Religious leader & school principle, man, Colombo)*  *“And also there's like a language barrier because a lot of social workers depends on if they speak English or not. And I guess people like me would want to speak to someone who speaks English. Something like that.” (IT worker, woman, Colombo)*  *“let's say they are monolingual, they only speak Sinhala or Tamil. They don't have insights because they don't know a second language.” (Teacher, man, Badulla)*  ***Social norms and structures:***  *“Again, it's important for us to pay attention to the cultures of South Asian families, their family structures, how men are perceived, how women are perceived, the gender roles that have such a big influence on people's lives, and how we should deal with those things.[…] it's a very complicated and complex situation, that can only be understood by someone who lives here, to be honest, as well.” (Mental health worker, woman, Colombo)*  *“Because again, there is a way to talk about these things, there’s a way to navigate through these issues. And it has to be connected to a culture that is so deeply rooted within the people.” (Mental health worker, woman, Colombo)* | | Statements in which participants refer to tailoring any non-specialist mental health interventions to cultural /social norms. |
| 4.1.7 Providing means of transportation | Participants emphasized the importance to provide transportation possibilities for non-specialists. | *“we observe during the Covid and during last crisis period, people don't come to clinic because of the transport issues. And they had no money for that and they had other priorities. So they stop their medication and that trigger another episode, sometime then they need to hospitalize. So we need a very good supportive mechanism for them to like resolve or recover soon.” (Mental health worker, woman, Colombo)*  *“That person maybe I think you could provide him with a small bike or something like that.” (Banker, man, Colombo)*  *“In Sri Lanka, midwives are given scooter bikes, like small bikes for them to visit houses. So something like that. I mean, if you could organize, then it'll be very successful. Otherwise, here the transportation is bit difficult in Sri Lanka. […] And also you have to go to interior places. All of them are not in the main roads, because of that.” (Banker, man, Colombo)* | | Statements in which participants mention the importance of transportation when implementing such interventions. |
| ***Type 4: The school-based educational outreach intervention type***  ***Definition: This intervention is a school-based outreach intervention type in which teachers receive basic mental health education to teach students on mental health and raise general mental health awareness and to be able to identify, refer and deal with children with mental health problems.*** | | | |  |
| 4.4.1 Involving parents | Participants believed in the benefits of involving the parents in such interventions, in form of small workshops and seminars. | *“if the schools can cover at least the foundation level for the parents of their students, like have a seminar at least twice a month. Maybe one seminar for the term and encourage them more and educate them on what's going on with the kids and how do you handle the temperament and what to deal with and all that.” (Mental health worker, woman, Colombo)*  *“But parenting is a very big impact on that. We can't do one aspect, we have to focus on everything. And the parent’s things and environment are the main major impact on the student's life and their thinking and their behavior.” (Mental health worker, woman, Colombo)*  *“The other thing is they need to give awareness to the parents” (Mental health worker, man, Badulla)*  *“So even the parents were very happy because we conduct a session for parents too. And they were also happy because, one, they were aware of the struggles that the children were going through.” (Mental health worker, woman, Colombo)*  ***„****You can educate the parents. I mean, educating is something else. But then again if they are also involved in this, then there will be issue. You can educate them. That is good.  You know, then maybe now some parents may not be aware of these things now. So educating them is good.“ (Banker, man, Colombo)* | | Statements in which participants express the need to involve parents in educational outreach interventions (with specific examples) |
| 4.4.2 Avoiding the illness angle | Participants expressed the importance of avoiding the illness-related angle in such outreach interventions to decrease the associated stigma with mental illness:  In educational session for students:   - using a more open-minded approach instead of focusing on illness labelling - discussion relatable problems of students   When involving parents:   - focussing on academic success as a consequence of good mental health when involving parents | ***For students:***  *“I think, when we use the term mental health, it sounds like a more medicalized thing. Whereas I think, going back to things like your rights, and well-being, what are the signs to look for? It is mental health but breaking it down into more friendly or less medical-sounding topics. Because if you go to school, and the teachers run a program about mental health, already, the children are coming with stigma, with taboo, with-- because in Sri Lanka, a lot of the language around the colloquial language, around mental health in Sri Lanka is also very damaging. So, the children would also kind of come to it with that perception.” (Mental health worker, woman, Colombo)*  *“You might like address something that you're like really going through, like conflicts with parents or body image or self-image or things like that. So then it's going to like kind of technically all right. This is something I am going through, so this is easier. And look, there's something that I can do about it. So it'll depend on how you do it.” (Mental health worker, woman, Colombo)*  *“I mean, if you teach them what depression is, you wouldn't want them to Google it, self-diagnose themselves. You would also recommend them to learn it in a way that they want to go next. So I think it would be great if it we teach like basic coping strategies, or how do you reach out to support if you can't reach out to the teacher directly, or things like that.” (Mental health worker, woman, Colombo)*  ***For parents:***  *„I think particularly if you're going to watch more the government schools, for parents to be engaged or to commit their child properly to something like this, you would need to tie it into the future financial or educational success.” (Mental health worker, woman, Colombo)* | | Statements in which participants refer to the importance of avoiding the illness angle in educational outreach interventions (with specific examples) |
| 4.4.3 Considering differences in schools | Participants emphasised the importance of considering differences between schools:   - (International) private schools have more funding and thus more mental health programs compared to governmental schools. - Popular schools have more students whereas unpopular schools don’t have enough students. - Greater illness burden in local schools - More mental health programs in private / international schools | *“So there are vast differences between government schools. So, if you're looking at smaller, less privileged government schools, then I would say they're about the same from Western Province to out of Western Province. You get the big government schools, which are very famous in the country, there's a lot of competition, and a lot of very wealthy or very influential people send their children to those schools. So then, those schools would be very different. And those schools would have people with different perceptions”(Mental health worker, woman, Colombo)*  *“So in private schools, we hire qualified people especially those who are with at least a master's degree in psychology and counseling. At the same time to work in special units. We have in our local university system, a Bachelor of Education in Special Education. So those are generally qualified people to work with children in schools. But I don't see them in government public schools. […] They can't afford to have them” (Religious leader & school principle, man, Colombo)*  *“In private schools, they take the initiative because, you know, they have money, to be honest. And now in public schools, it is not happening. There are no counselors.” (Religious leader & school principle, man, Colombo)*  *“I feel like international schools will benefit from it, but they already have tons of those. They already have funding and international backing and all.” (Mental health worker, woman, Colombo)*  *“So these popular schools are having children up to 50. And on the other hand, there are unpopular school. They can't even find these students. So because of this high number in the so-called popular school, there may be problems in implementing this kind of program by the teacher.” (Mental health worker, woman, Colombo)*  *“So there's international schools and local schools. Local schools have had suicides because of teachers and their parents; them not being able to tell their parents that they passed or that they failed. And because of that, they think that the means of taking their life is easier than coming to their parents with these grades.” (IT worker, woman, Colombo)*  *“But a lot of the international schools have had a lot of psychology teachers and stuff.” (IT worker, woman, Colombo)* | | Statements in which participants explain any differences between schools that need to be considered when implementing the educational outreach intervention. |
