# Supplementary material for: Identifying contextual barriers and facilitators in implementing non-specialist interventions for mental health in Sri Lanka: A qualitative study with mental health workers and community members
Source: Glob Ment Health (Camb). 2024 Oct 8;11:e76. doi: 10.1017/gmh.2024.75 (PMC11504943; doi:10.1017/gmh.2024.75)
Supplement: Wijekoon Mudiyanselage et al. supplementary material [file S205442512400075Xsup001.zip › Additional file 10 Other strategies to decrease the mental healthcare gap in Sri Lanka.docx]

# **Additional file 10: Coding framework for other ideas to reduce the mental healthcare gap in Sri Lanka**

| **Code** | **Definition** | **Example Quotes** | **Coding rule** |
| --- | --- | --- | --- |
| 1.1 Outreach in work settings | Participants emphasized the importance to offer outreach interventions in work settings. They specifically focussed on:   - Raising awareness - Handling work-related stress - Reaching the whole community (i.e., parents of children) | *“[…]So that is also somewhere you could promote mental health awareness. You could go to banks, insurance companies, not only schools, higher education institutes, and all corporate offices. [… ]From the organizations, if there are ways to arrange workshops and seminars, and educate on how to handle your colleagues or how to handle the work stress and things like that. Those things should be promoted. (Mental health worker, woman, Colombo)*  *“it’s difficult to catch the parents [in school outreach interventions] because it is the only way we can create awareness from their working places. So, we have to go and do the programs in workplaces. That is one way.” (Mental health worker, man, Badulla)*  *“He believes that this [the educational outreach intervention] will definitely help if we take it outside into other institutions because there is a lot of mental health issues in Sri Lanka the way that he sees it. And those need to be addressed. There's a lot of stress, a lot of mental distress as well. And even in the police station, he said that because there's so much work and they are sometimes overwhelmed with the amount of work that they have to handle. The implementation of a system like this will assist those people to do their jobs better as well and be healthy. So he believes that this can be implemented in institutions outside the education system.” (Police officer, man, Badulla)* | *Any example of outreach interventions in work-settings* |
| 1.2 Outreach in media | Participants emphasized the importance to implement outreach interventions in form of awareness and education on the media:   - Sri Lankan teledramas - Social media - Newspaper | *“There is a real reliance on these teledramas […] And so, these are things that people are hooked on. […]* *A lot of the negative social attitudes come from these programs. So lots of gender-based stuff, lots of relationship abuse, lots of child-parent abuse, a lot of these ideas of what is good, and what is bad, a lot of homophobia. When you watch these programs they promote, I don't know, whether consciously and actively, but even unconsciously, they heavily promote these very, very toxic ideas and concepts. So having a teledrama, I've always thought that counted the things that encouraged good mental health, good practices within families, good practices within relationships, good communication skills, and kind of use that kind of vehicle as a way to get ideas and teach skills to people. That will be amazing. And it would work very, very well, if it had a good cost and good writers, but obviously, that's a big budget.” (Mental health worker, woman, Colombo)*  *“[…] through media, especially through media, if we can get this through to a national TV channel, or get it through to one of the main Sri Lanka newspapers like Lakbima or The Daily Mirror or like something that everyone has access to” (Mental health worker, woman, Colombo)*  *“We can give them awareness through videos and articles, through Facebook or WhatsApp, and we can write articles. We can give some two, three minutes video about that. So people will get to know because everyone is watching videos these days. Everyone is on YouTube, Facebook, and everything. Yeah, it's a very good way to reach them.” (Mental health worker, woman, Colombo)* | *Any example of outreach interventions in media* |
