# Supplementary figures and images for: Identifying contextual barriers and facilitators in implementing non-specialist interventions for mental health in Sri Lanka: A qualitative study with mental health workers and community members
Source: Glob Ment Health (Camb). 2024 Oct 8;11:e76. doi: 10.1017/gmh.2024.75 (PMC11504943; doi:10.1017/gmh.2024.75)

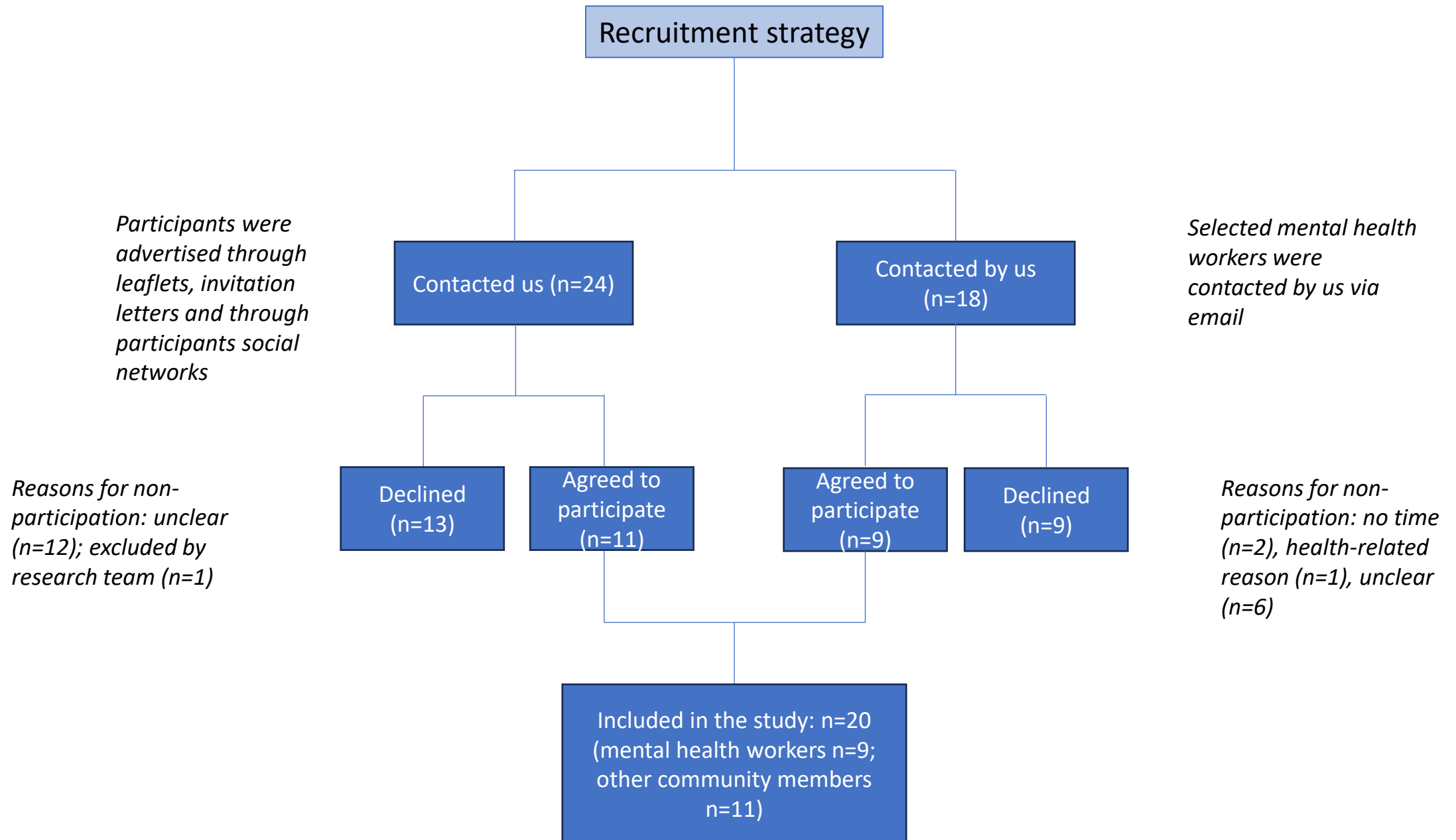

Supplement: Wijekoon Mudiyanselage et al. supplementary material [file S205442512400075Xsup001.zip › Additional file 2 Recruitment process.pdf]
