# Supplementary material for: Identifying contextual barriers and facilitators in implementing non-specialist interventions for mental health in Sri Lanka: A qualitative study with mental health workers and community members
Source: Glob Ment Health (Camb). 2024 Oct 8;11:e76. doi: 10.1017/gmh.2024.75 (PMC11504943; doi:10.1017/gmh.2024.75)
Supplement: Peer review history

# Editor report: Recommendation: Identifying contextual barriers and facilitators in implementing non-specialist interventions for mental health in Sri Lanka: A qualitative study with mental health workers and community members — R0/PR2

**DOI:** 10.1017/gmh.2024.75.pr2

**Affiliations:** Psychiatry , Africa Mental Health Research and, Kenya

This study makes a significant contribution by enriching the Global data pool in addition to doing useful to Sri Lanka. The literature review, methodology and discussion are satisfactory. I appreciate it is qualitative study. However, they should state the general objective and specific aims more clearly than it is currently.

# Editor report: Decision: Identifying contextual barriers and facilitators in implementing non-specialist interventions for mental health in Sri Lanka: A qualitative study with mental health workers and community members — R0/PR3

**DOI:** 10.1017/gmh.2024.75.pr3

**Affiliations:** London School of Hygiene & Tropical Medicine, United Kingdom of Great Britain and Northern Ireland

No accompanying comment.

# Editor report: Recommendation: Identifying contextual barriers and facilitators in implementing non-specialist interventions for mental health in Sri Lanka: A qualitative study with mental health workers and community members — R1/PR5

**DOI:** 10.1017/gmh.2024.75.pr5

**Affiliations:** Psychiatry , Africa Mental Health Research and, Kenya

I note that the authors have undertaken extensive revisions of the paper which make it more focused in addressing the key issues. 

# Editor report: Decision: Identifying contextual barriers and facilitators in implementing non-specialist interventions for mental health in Sri Lanka: A qualitative study with mental health workers and community members — R1/PR6

**DOI:** 10.1017/gmh.2024.75.pr6

**Affiliations:** London School of Hygiene & Tropical Medicine, United Kingdom of Great Britain and Northern Ireland

No accompanying comment.
